# Supplementary material for: Multigenerational paternal obesity enhances the susceptibility to male subfertility in offspring via Wt1 N6-methyladenosine modification
Source: Nat Commun. 2024 Feb 14;15:1353. doi: 10.1038/s41467-024-45675-4 (PMC10866985; doi:10.1038/s41467-024-45675-4)
Supplement: Supplementary file 1 — Supplementary Materials [file 41467_2024_45675_MOESM1_ESM.pdf]

Supplementary Figure 1

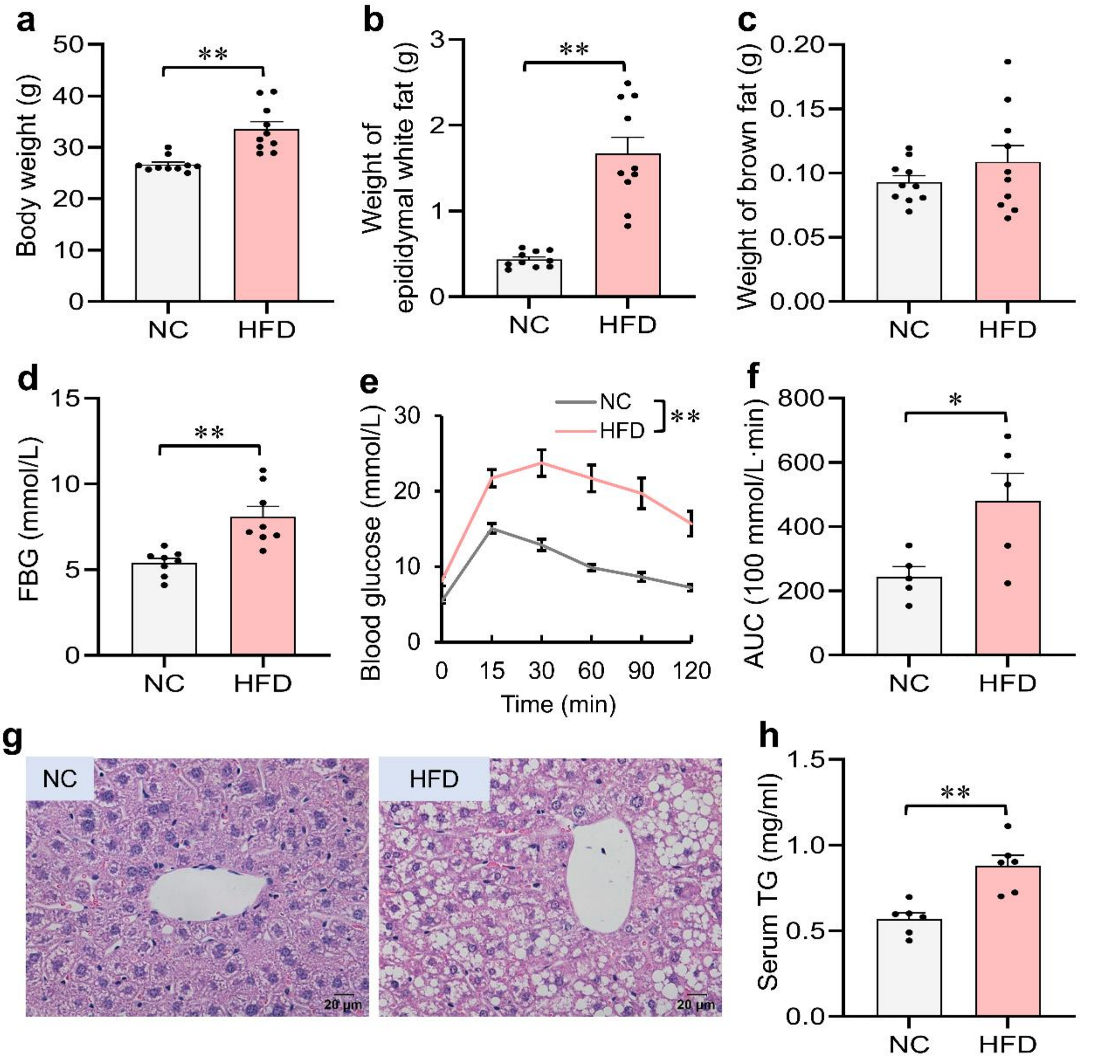

Supplementary Fig.1. HFD induces obesity phenotype in mice. F0 generation male mice were fed NC or HFD from 5 weeks to 15 weeks old. (**a**) Body weight of mice. *n*=10 mice, *t*=-4.59, *P*=0.0008. (**b**) The weight of epididymal white fat. *n*=10 mice, DOF=19, *t*=-6.45, *P*<0.0001. (**c**) The weight of brown fat. *n*=10 mice, DOF=19, *t*=-1.15, *P*=0.2745. (**d**) Fasting blood glucose (FBG). *n*=8 mice, DOF=15, *t*=-4.07, *P*=0.0025. (**e**) Glucose tolerance test (GTT) was performed. *n*=8 mice, *P*<0.0001 (**f**) AUC for GTT. *n*=5 mice, DOF=9, *t*=-2.57, *P*=0.0332. (**g**) Liver H&E staining. (**h**) Serum triglyceride (TG) was detected by ELISA. *n*=6 mice, DOF=11, *t*=-4.33,

P=0.0024. **P* < 0.05; ***P* < 0.01 vs NC. In regard to Supplementary Fig.1a-d, f, h, statistical significance was evaluated by two-tailed *t* test. Repeated-measures *ANOVA* was applied to analyze the GTT data. Data are presented as *mean* ± *SEM*. Source data are provided with this paper.

Supplementary Figure 2

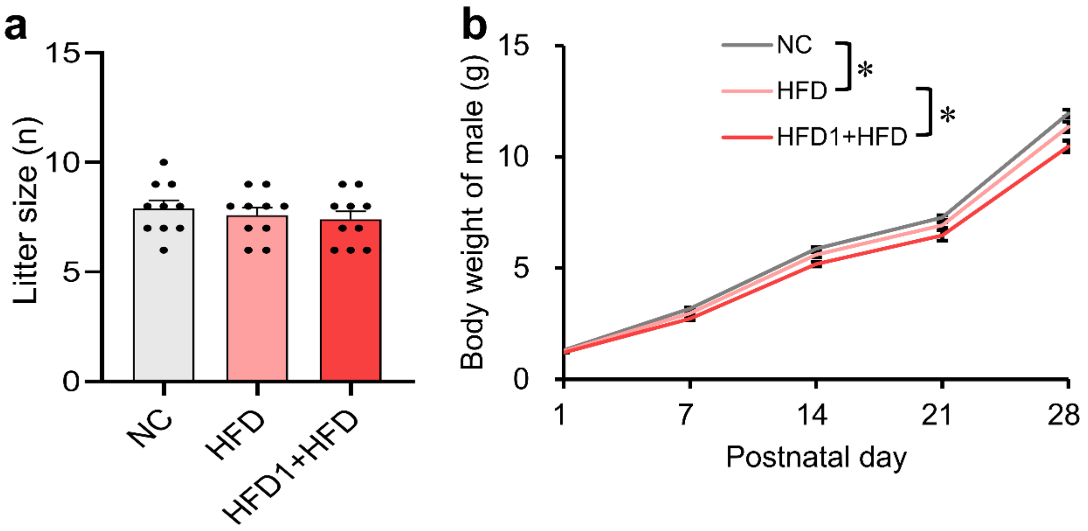

Supplementary Fig.2. The effect of multigenerational paternal HFD on litter size and body weight in offspring. F0 generation male mice were fed NC or HFD from 5 weeks to 15 weeks old, and then mated with NC-fed female mice to breed F1 generation. Similarly, a subset of the males in F1 generation were continued to be treated with HFD for 10 weeks, and mated with normal female to breed F2 generation. (**a**) Litter size. *n*=10 mice, DOF=29, *F*=0.48, *P*=0.6246. Statistical significance was evaluated by two-sided one-way *ANOVA*. (**b**) Body weight of male. *n* = 10 mice, *F*=31.45， *P*＜0.0001. Two-way *ANOVA* was applied to analyze mouse body weight data. **P* < 0.05. Data are presented as *mean* ± *SEM*. Source data are provided with this paper.

Supplementary Figure 3

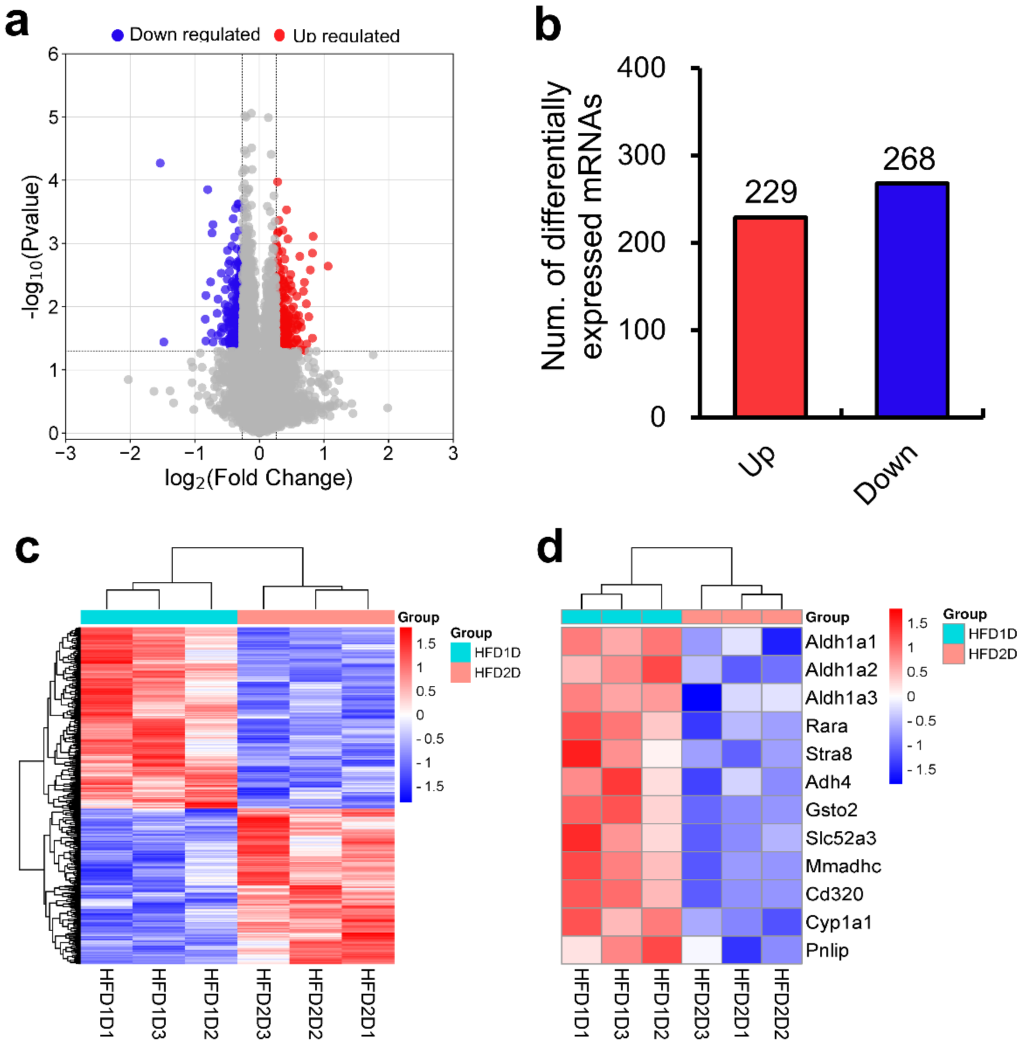

Supplementary Fig.3. Multigenerational paternal HFD gradually exacerbates environmental stress-induced testicular dysregulated expression of mRNAs in offspring. F0 generation male mice were fed NC or HFD from 5 weeks to 15 weeks old, and then mated with NC-fed female mice to breed F1 generation. Similarly, a subset of the males in F1 generation were continued to be treated with HFD for 10 weeks, and mated with normal female to breed F2 generation. Male mice of F1 and F2 generations were exposed to $CdCl_2$ (0 or 100 mg/L) by drinking water for 10 weeks, and named HFD1D or HFD2D group respectively. Testes of HFD1D and HFD2D groups were

collected for RNA-seq. (**a**) The distribution of differentially expressed mRNAs in testes were presented. (**b**) The number of up-regulated and down-regulated mRNAs was shown. (**c**) Heatmap of differentially expressed mRNAs in testes was presented. (**d**) Heatmap of retinol metabolic process-related mRNAs in testes was presented.

Supplementary Figure 4

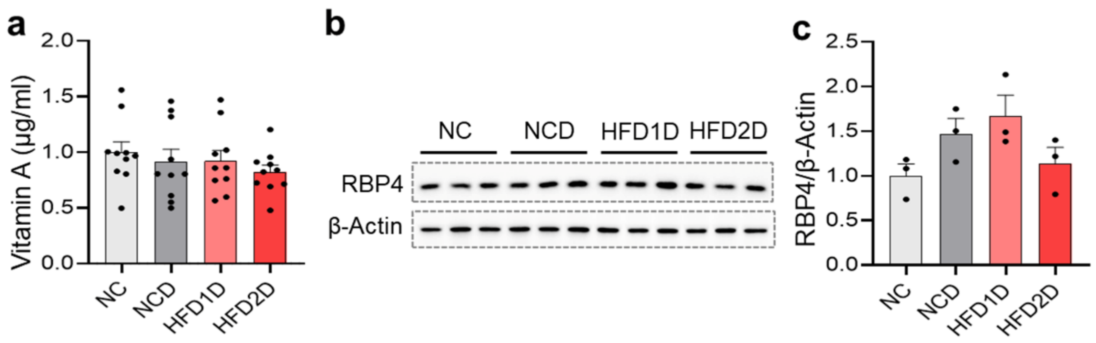

Supplementary Fig.4. The effect of multigenerational paternal HFD on the level of vitamin A and RBP4 in offspring. F0 generation male mice were fed NC or HFD from 5 weeks to 15 weeks old, and then mated with NC-fed female mice to breed F1 generation. Similarly, a subset of the males in F1 generation were continued to be treated with HFD for 10 weeks, and mated with normal female to breed F2 generation. Male mice of F1 and F2 generations were exposed to $CdCl_2$ (0 or 100 mg/L) by drinking water for 10 weeks, and named NC, NCD, HFD1D or HFD2D group respectively. (**a**) Serum vitamin A was measured by ELISA. *n*=10 mice, DOF=39, *F*=0.47, *P*=0.7031. (**b** and **c**) Testicular RBP4 protein expression was measured by immunoblotting. *n*=3 mice, DOF=11, *F*=2.76, *P*=0.1113. Statistical significance was evaluated by two-sided one-way *ANOVA* with post hoc *LSD* tests. Data are presented as *mean* ± *SEM*. Source data are provided with this paper.

Supplementary Figure 5

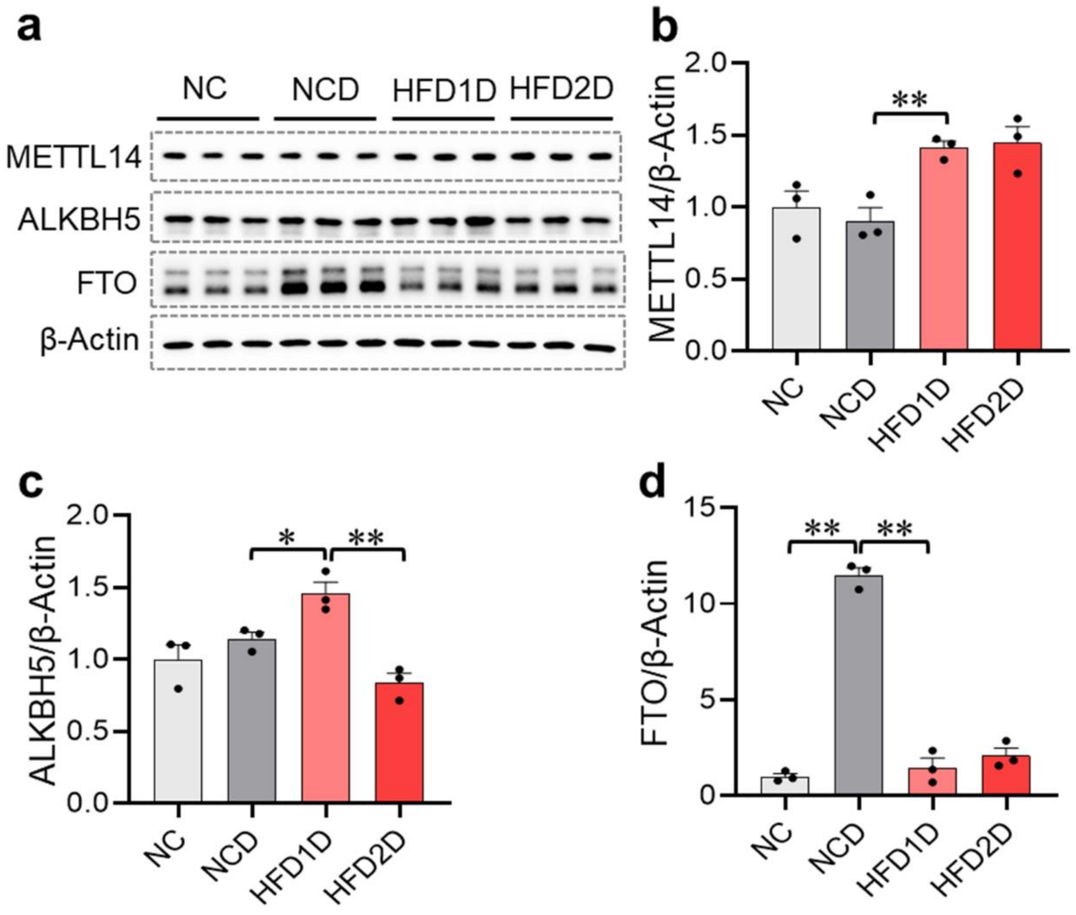

Supplementary Fig.5. Effect of multigenerational paternal HFD on testicular METTL14, ALKBH5 and FTO expression in offspring. F0 generation male mice were fed NC or HFD from 5 weeks to 15 weeks old, and then mated with NC-fed female mice to breed F1 generation. Similarly, a subset of the males in F1 generation were continued to be treated with HFD for 10 weeks, and mated with normal female to breed F2 generation. Male mice of F1 and F2 generations were exposed to $CdCl_2$ (0 or 100 mg/L) by drinking water for 10 weeks, and named NC, NCD, HFD1D or HFD2D group respectively. (**a-d**) Testicular METTL14, ALKBH5 and FTO proteins expression were measured by immunoblotting. n=3 mice, DOF=11, F=8.85 and P=0.0064 for METTL14; F=12.19 and P=0.0021 for ALKBH5; F=182.34 and $P<0.001$ for FTO.

*P < 0.05; **P < 0.01. Statistical significance was evaluated by two-sided one-way *ANOVA* with post hoc *LSD* tests. Data are presented as *mean* ± *SEM*. Source data are provided with this paper.

Supplementary Figure 6

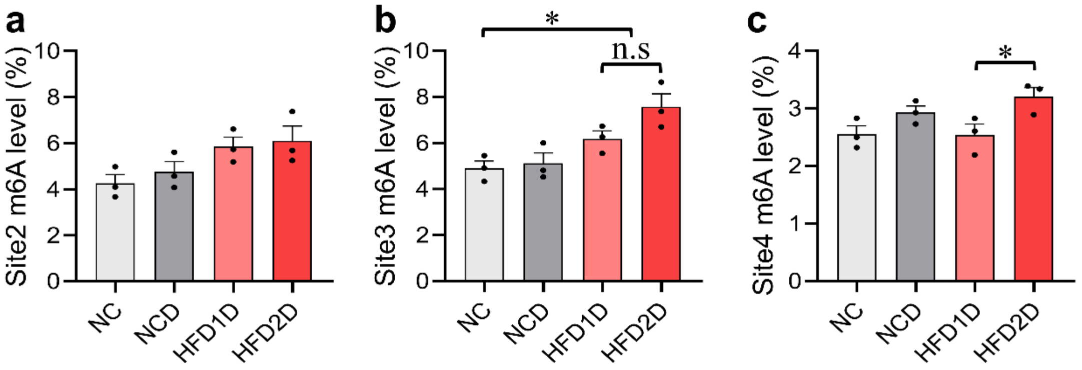

Supplementary Fig.6. Effect of multigenerational paternal HFD on testicular *Wt1* Site2-4 m6A modification in offspring. F0 generation male mice were fed NC or HFD from 5 weeks to 15 weeks old, and then mated with NC-fed female mice to breed F1 generation. Similarly, a subset of the males in F1 generation were continued to be treated with HFD for 10 weeks, and mated with normal female to breed F2 generation. Male mice of F1 and F2 generations were exposed to $CdCl_2$ (0 or 100 mg/L) by drinking water for 10 weeks, and named NC, NCD, HFD1D or HFD2D group respectively. (**a-c**) Testicular *Wt1* site 2, 3 and 4 m6A levels were detected by MeRIP-qPCR. *n*=3 mice, DOF=11, *F*=3.29 and *P*=0.0791 for Site2; *F*=7.97 and *P*=0.0087for Site3; *F*=4.35 and *P*=0.0428 for Site4. n.s., not significant. *P < 0.05. Statistical significance was evaluated by two-sided one-way *ANOVA* with post hoc *LSD* tests. Data are presented as *mean* ± *SEM*. Source data are provided with this paper.

Supplementary Figure 7

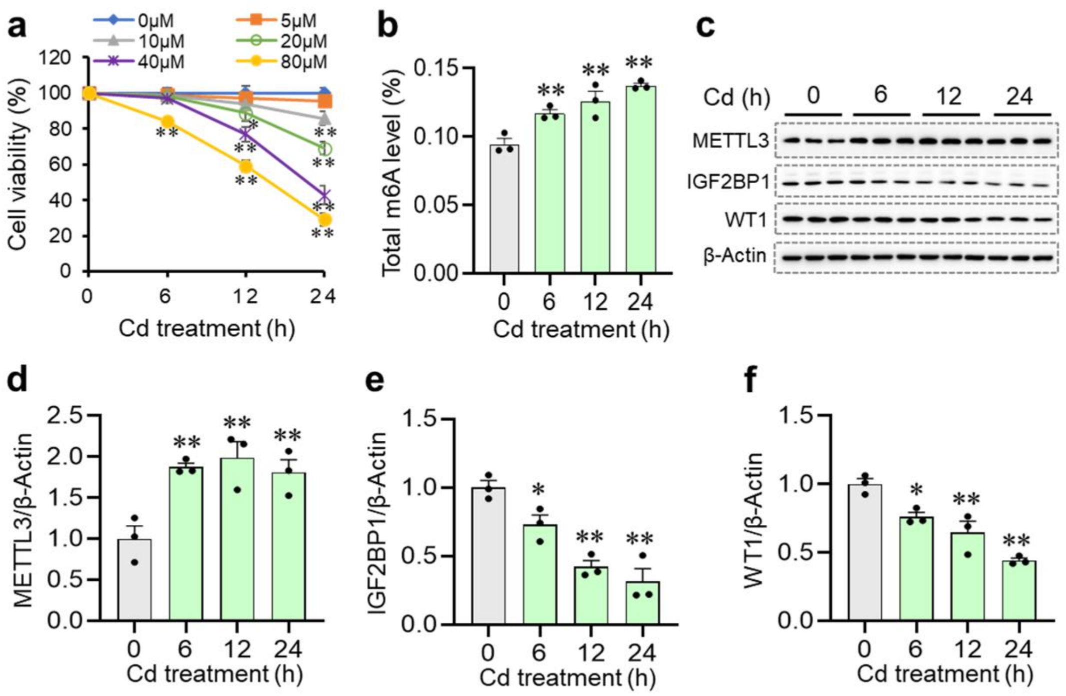

Supplementary Fig.7. Cd increases the level of m6A modification in Sertoli cell. (**a**) Cell viability of TM4 was detected after exposure to different concentrations of $CdCl_2$ for different time points. (**b**–**f**) Treatment of TM4 cells with $CdCl_2$ (20 μM) for 0, 6, 12 or 24 h. (**b**) Cellular total RNA m6A levels were measured. n=3 biologically independent samples, DOF=11, F=17.14, P=0.0007. (**c-f**) Cellular METTL3, IGF2BP1 and WT1 protein levels were detected via immunoblotting. n=3 biologically independent samples, DOF=11, F=9.05 and P=0.0060 for METTL3; F=20.86 and P=0.0004 for IGF2BP1; F=21.68 and P=0.0003 for WT1. *$P < 0.05$; **$P < 0.01$ *vs* 0 h. Statistical significance was evaluated by two-sided one-way *ANOVA* with post hoc *LSD* tests. Data are presented as *mean* ± *SEM*. Source data are provided with this paper.

Supplementary Figure 8

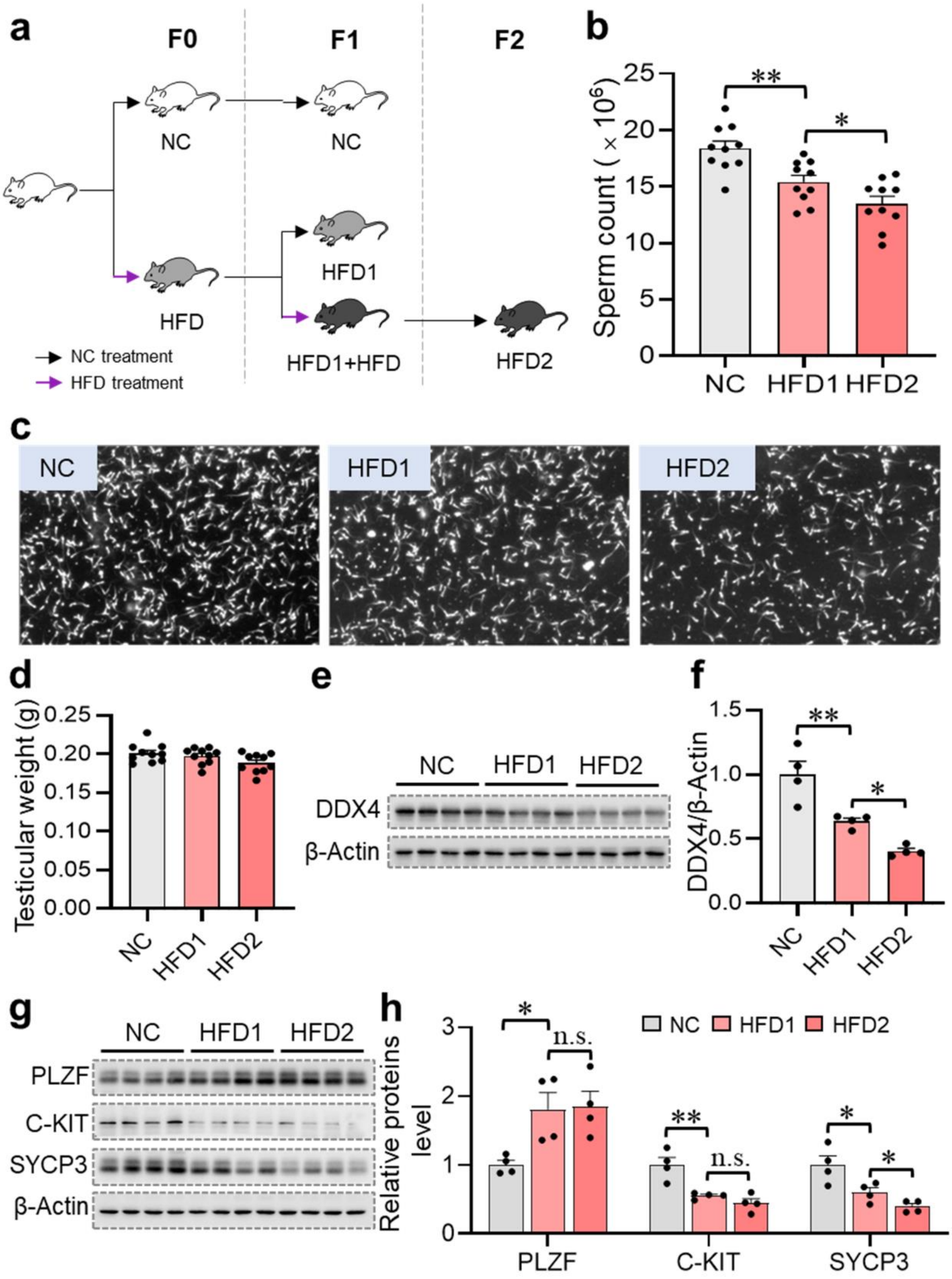

Supplementary Fig.8. The effects of paternal single- or double-generation HFD exposure on testicular development and spermatogenesis in offspring mice. F0 generation male mice were fed NC or HFD from 5 weeks to 15 weeks old, and then

mated with NC-fed female mice to breed F1 generation. Similarly, F1 generation male mice whose fathers were exposed to HFD continued to be fed HFD for 10 weeks, and mated with normal female to breed F2 generation. Male mice of F1 and F2 generations named NC, HFD1 or HFD2 group respectively. All mice were euthanized at 15 weeks of age. (**a**) Experimental design flowchart. The black arrow indicated NC treatment. The purple arrow indicated HFD treatment. (**b** and **c**) Epididymal sperm counts were measured. n=10 mice, DOF=29, F=15.19, $P<0.0001$. (**d**) Testicular weight. n=10 mice, DOF=29, F=3.09 P=0.0618. (**e** and **f**) Testicular DDX4 protein expression was detected by immunoblotting. n=4 mice, DOF=11, F=22.85, P=0.0003. (**g** and **h**) Testicular PLZF, C-KIT and SYCP3 protein expression were detected by immunoblotting. n=4 mice, DOF=11, F=6.20 and P=0.0203 for PLZF; F=15.91 and P=0.0011 for C-KIT; F=11.60 and P=0.0032 for SYCP3. n.s., not significant. *$P < 0.05$; **$P < 0.01$. Statistical significance was evaluated by two-sided one-way *ANOVA* with post hoc *LSD* tests. Data are presented as *mean* ± *SEM*. Source data are provided with this paper.

Supplementary Figure 9

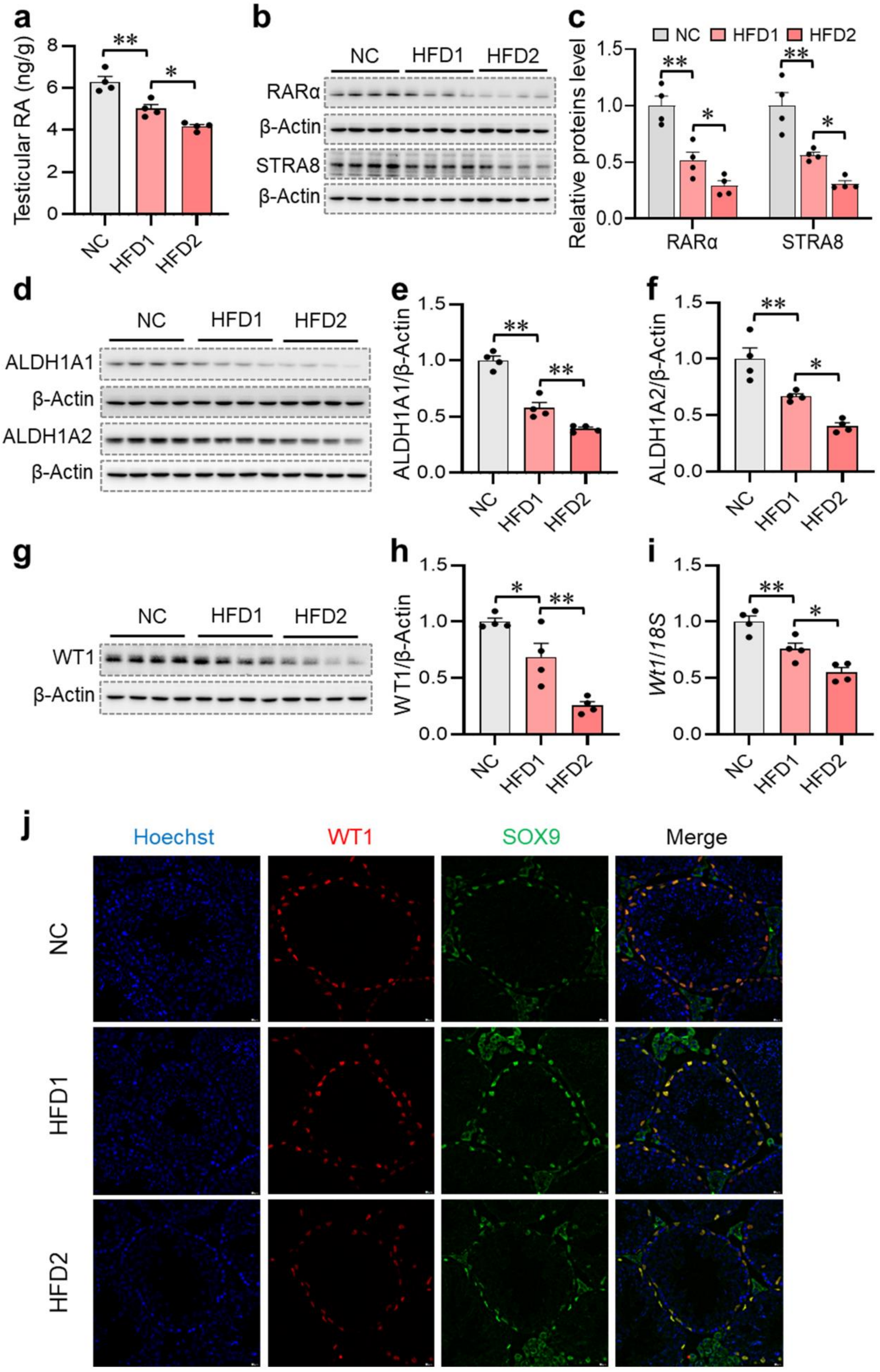

Supplementary Fig.9. The effects of paternal single- or double-generation HFD exposure on testicular retinoic acid synthesis and WT1 expression in offspring mice. F0 generation male mice were fed NC or HFD from 5 weeks to 15 weeks old, and then mated with NC-fed female mice to breed F1 generation. Similarly, F1 generation male mice whose fathers were exposed to HFD continued to be fed HFD for 10 weeks, and mated with normal female to breed F2 generation. Male mice of F1 and F2 generations named NC, HFD1 or HFD2 group respectively. All mice were euthanized at 15 weeks of age. (**a**) Testicular retinoic acid level was detected by ELISA. n=4 mice, DOF=11, F=30.91, $P<0.0001$. (**b** and **c**) Testicular RARα and STRA8 protein expression were measured by immunoblotting. n=4 mice., DOF=11, F=26.55 and P=0.0002 for RARα; F=24.69 and P=0.0002 for STRA8. (**d**-**f**) Testicular ALDH1A1 and ALDH1A2 protein expression were measured by immunoblotting. n=4 mice, DOF=11, F=73.44 and $P<0.0001$ for ALDH1A1; F=24.17 and P=0.0002 for ALDH1A2. (**g** and **h**) Testicular WT1 protein expression was measured by immunoblotting. n=4 mice, DOF=11, F=23.93, P=0.0002. (**i**) Testicular *Wt1* mRNA level was tested using RT-qPCR. n=4 mice, DOF=11, F=20.85, P=0.0004. (**j**) Representative testicular images of immunofluorescent staining for WT1 and SOX9. Scale bar, 20 μm. Hoechst33258 were used to tag the nuclei. $*P < 0.05$; $**P < 0.01$. Statistical significance was evaluated by two-sided one-way *ANOVA* with post hoc *LSD* tests. Data are presented as *mean* ± *SEM*. Source data are provided with this paper.

Supplementary Figure 10

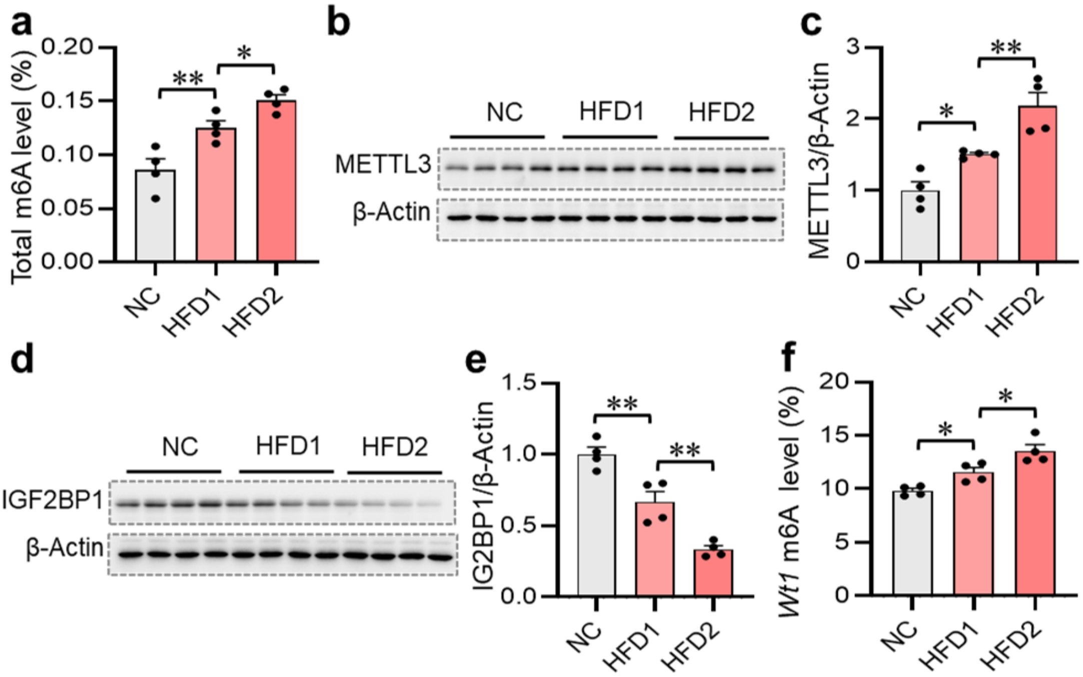

Supplementary Fig.10. The effects of paternal single- or double-generation HFD exposure on testicular METTL3 expression and *Wt1* m6A level in offspring mice. F0 generation male mice were fed NC or HFD from 5 weeks to 15 weeks old, and then mated with NC-fed female mice to breed F1 generation. Similarly, F1 generation male mice whose fathers were exposed to HFD continued to be fed HFD for 10 weeks, and mated with normal female to breed F2 generation. Male mice of F1 and F2 generations named NC, HFD1 or HFD2 group respectively. All mice were euthanized at 15 weeks of age. (**a**) Testicular total RNA m6A level was measured. n=4 mice, DOF=11, F=18.14, P=0.0007. (**b** and **c**) Testicular METTL3 protein expression was detected by immunoblotting. n=4 mice, DOF=11, F=20.14, P=0.0005. (**d** and **e**) Testicular IGF2BP1 protein expression was detected by immunoblotting. n=4 mice, DOF=11, F=38.67, P<0.0001. (**f**) *Wt1* site1 m6A level was measured by MeRIP-qPCR. n=4 mice, DOF=11, F=17.14, P=0.0009. *P < 0.05. Statistical significance was evaluated

by two-sided one-way *ANOVA* with post hoc *LSD* tests. Data are presented as *mean* ± *SEM*. Source data are provided with this paper.

Supplementary Figure 11

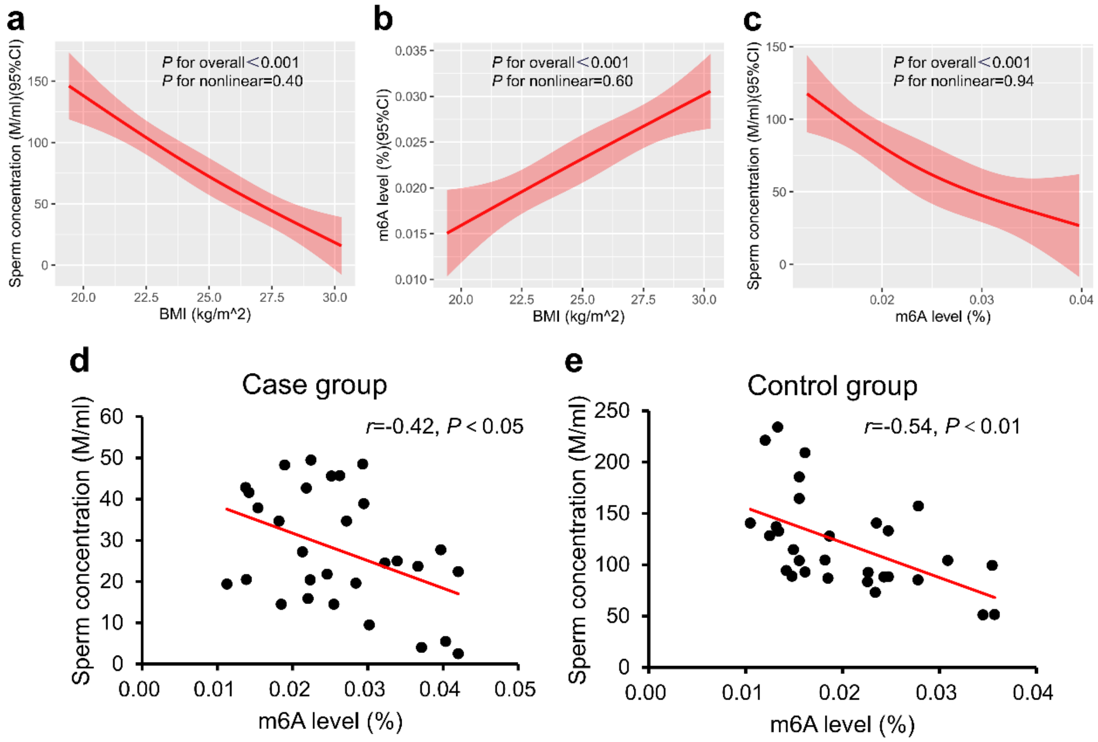

Supplementary Fig.11. The association among sperm concentration, BMI and sperm m6A level were analyzed. After removal of smoking or alcohol drinking donors, 30 pairs of cases with overweight/obesity and corresponding controls were obtained by matching age. (**a-c**) Restricted cubic splines were used to describe the nonlinear correlation among sperm concentration, BMI and sperm m6A level. (**d** and **e**) The linear correlation analysis of sperm concentration and sperm m6A level was performed in both the case and control groups, respectively.

Supplementary Table 1

Primers for real-time RT-PCR.

Genes	Sequences	Species
18S	**F:** GTAACCCGTTGAACCCCATT **R:** CCATCCAATCGGTAGTAGCG	M
Plzf	**F:** GTTGGGGGTCAGCTAGAAAGC **R:** TTCCTTCTCTGCTCTGCAAGG	M
C-kit	**F:** CCTCTGGGAGCTCTTCTCCT **R:** TGTTGGCCTTTTCAAGGGGT	M
Smc3	**F:** GCTCTGAATGATGAAATCCGTC **R:** GCGTTTCCTCAGATTCTCATTC	M
Acrv1	**F:** GAATCAAGTGAGCATGCTGTAG **R:** CTGAAGTGTGTTCTGACAAGTG	M
Lzumo3	**F:** TCTCACATCGGAGGAAAATGAA **R:** CCAGTTTGCATTTCTGACTCAG	M
Aldh1a1	**F:** TCAGAGCAGGAGCCAGAA **R:** ACACCATACCCCAAACTCCA	M
Aldh1a2	**F:** CCGTGTTCTCCAACGTCACT **R:** CCGTAAGCCAAACTCACCCA	M
Aldh1a3	**F:** CGGAGAGTGCGAACCAGTTA **R:** TCCACTCTTGGATTCGTGCC	M
Rdh10	**F:** AGACACGGGCATGTTCAGAG **R:** CACACGACGGCTTCAAAAGG	M
Cyp26a1	**F:** TCTGGGACCTGTACTGTGTGA **R:** AAGCCGTATTTCCTGCGCTT	M
Cyp26b1	**F:** TGCCCATACCCCATCGCC **R:** GGCTGCGAGGTGATCGAAGA	M
Cyp26c1	**F:** CCTCTTCTCACTGCCCTTGG **R:** CTGGCTGTGGTGAAAAAGGC	M
Wt1	**F:** CCCAGCTTGAATGCATGACC **R:** CTGACCGGACAAGAGTTGGG	M
Mettl3	**F:** GAGCTAGGATGTCGGACACG **R:** GCCGCTTCTGGGTTCCTTA	M
Mettl14	**F:** GCTTGCGAAAGTGGGGTTAC **R:** AATGAAGTCCCCGTCTGTGC	M
Wtap	**F:** CATTTTGTGGCTGCGAGACC **R:** ACTCATCCCGTGCCATAACT	M
Alkbh5	**F:** ATTGCCACCCAGCTATGCTT **R:** AGACCGCCGGTTTTCTTCTT	M
Fto	**F:** AGAACCTGGTGGACAGGTCA **R:** CTGGTGTCTCGATGTCCCAA	M
Ythdf1	**F:** GCCCTACCTTTCTGGACAGT **R:** AGATACGGGATGGGAGGGTC	M
Ythdf2	**F:** AGGATCCGAGAGCCATGTCG **R:** TATTCGGCCTTGCCTGTGG	M
Ythdf3	**F:** CAGAGACCTAAAGGGCAAGGA **R:** GGAAATCCAATGGACGGAGC	M
Ythdc1	**F:** CGGGAGGAGAAAGATGGGGA **R:** TGTCGCTTGGTGTCAGTAGAC	M
Ythdc2	**F:** TGACCAGTACGGAAAGAGCC **R:** GGTCATCATTGCATGAGCTGT	M
Igf2bp1	**F:** GCTGTGGTCAACGTCACCTA **R:** CCATTCTCAGGACCTTGCGT	M
Igf2bp2	**F:** ATTTCGGCCCATTCCCACAT **R:** GGACCTTCTGCTGGAGCAAT	M
Igf2bp3	**F:** TTCCTGGTGAAGACGGGCTA **R:** CATTGTAAGTGGGGCGGGAT	M

Supplementary Table 2

Primer sequence of *Wt1* for MeRIP-PCR.

Genes	Sequences	Species
Wt1-1253	**F:** CCCAGCTTGAATGCATGACC **R:** CTGACCGGACAAGAGTTGGG	M
Wt1-1548	**F:** CACGGCACAGGGTATGAGAG **R:** GTTGGGGCCACTCCAGATAC	M
Wt1-1682	**F:** CAGCGAAAGTTTTCCCGGTC **R:** ATGTTGTGATGGCGGACCAA	M
Wt1-2714	**F:** TCCGGTCAGCATCTGAAACC **R:** ATGAGTCCTGGTGTGGGTCT	M

Supplementary Table 3

Chemical reagents and antibodies

Chemical reagents and antibodies	Source	Identifier
$CdCl_2$	Sigma-Aldrich	202908
STM2457	Med Chem Express	HY-134836
Actinomycin D	Med Chem Express	HY-17559
MTT	Med Chem Express	HY-15924
Lipofectamin®3000	Invitrogen	L3000-015
Rabbit polyclonal anti-DDX4	Abcam	ab13840
Mouse polyclonal anti-PLZF	Santa Cruz Biotechnologies	sc-28319
Mouse polyclonal anti-C-KIT	Santa Cruz Biotechnologies	sc-365504
Mouse polyclonal anti- SYCP3	Santa Cruz Biotechnologies	sc-74569
Rabbit polyclonal anti-RBP4	Abcam	ab188230
Rabbit polyclonal anti-RARα	Cell Signaling Technology	62294S
Rabbit polyclonal anti-STRA8	Abcam	ab49602
Rabbit polyclonal anti-ALDH1A1	Abcam	ab52492
Rabbit polyclonal anti-ALDH1A2	Cell Signaling Technology	83805S
Rabbit polyclonal anti-WT1	Abcam	ab89901
Rabbit polyclonal anti-METTL3	Abcam	ab195352
Rabbit polyclonal anti-METTL14	Cell Signaling Technology	51104S
Rabbit polyclonal anti-ALKBH5	Abcam	ab195377
Mouse polyclonal anti-FTO	Abcam	ab92821
Rabbit polyclonal anti-YTHDF1	Proteintech	17479-1-AP
Rabbit polyclonal anti-YTHDF2	Proteintech	24744-1-AP
Rabbit polyclonal anti-IGF2BP1	Proteintech	22803-1-AP
Rabbit polyclonal anti-β-Actin	Sigma-Aldrich	A1978
