# Supplementary material for: Multigenerational paternal obesity enhances the susceptibility to male subfertility in offspring via Wt1 N6-methyladenosine modification
Source: Nat Commun. 2024 Feb 14;15:1353. doi: 10.1038/s41467-024-45675-4 (PMC10866985; doi:10.1038/s41467-024-45675-4)
Supplement: Supplementary file 3 — Reporting Summary [file 41467_2024_45675_MOESM3_ESM.pdf]

nature portfolio

Corresponding author(s): Hua Wang

Last updated by author(s): Dec 25, 2023

Reporting Summary

Nature Portfolio wishes to improve the reproducibility of the work that we publish. This form provides structure for consistency and transparency in reporting. For further information on Nature Portfolio policies, see our Editorial Policies and the Editorial Policy Checklist.

Statistics

For all statistical analyses, confirm that the following items are present in the figure legend, table legend, main text, or Methods section.

n/a	Confirmed	
☐	☒	The exact sample size (*n*) for each experimental group/condition, given as a discrete number and unit of measurement
☐	☒	A statement on whether measurements were taken from distinct samples or whether the same sample was measured repeatedly
☐	☒	The statistical test(s) used AND whether they are one- or two-sided<br>*Only common tests should be described solely by name; describe more complex techniques in the Methods section.*
☒	☐	A description of all covariates tested
☐	☒	A description of any assumptions or corrections, such as tests of normality and adjustment for multiple comparisons
☐	☒	A full description of the statistical parameters including central tendency (e.g. means) or other basic estimates (e.g. regression coefficient) AND variation (e.g. standard deviation) or associated estimates of uncertainty (e.g. confidence intervals)
☐	☒	For null hypothesis testing, the test statistic (e.g. *F*, *t*, *r*) with confidence intervals, effect sizes, degrees of freedom and *P* value noted<br>*Give P values as exact values whenever suitable.*
☒	☐	For Bayesian analysis, information on the choice of priors and Markov chain Monte Carlo settings
☒	☐	For hierarchical and complex designs, identification of the appropriate level for tests and full reporting of outcomes
☐	☒	Estimates of effect sizes (e.g. Cohen's *d*, Pearson's *r*), indicating how they were calculated

Our web collection on statistics for biologists contains articles on many of the points above.

Software and code

Policy information about availability of computer code

Data collection	Agilent Feature Extraction software (version 11.0.1.1)<br>StringTie software<br>The ENCORI database are available at https://starbase.sysu.edu.cn/index.php.<br>The ChEA3 database are available at https://maayanlab.cloud/chea3/.<br>R (Version 3.6.0) https://www.r-project.org/
Data analysis	All statistical analyses were conducted by SPSS23.0 software.<br>The data were graphically presented by GraphPad Prism 8.0 or R (Version 3.6.0) processing.<br>The protein signal was quantified by Image J software (Version 1.8.0, https://imagej.nih.gov/ij/).

For manuscripts utilizing custom algorithms or software that are central to the research but not yet described in published literature, software must be made available to editors and reviewers. We strongly encourage code deposition in a community repository (e.g. GitHub). See the Nature Portfolio guidelines for submitting code & software for further information.

Data

Policy information about availability of data

All manuscripts must include a data availability statement. This statement should provide the following information, where applicable:

- Accession codes, unique identifiers, or web links for publicly available datasets
- A description of any restrictions on data availability
- For clinical datasets or third party data, please ensure that the statement adheres to our policy

The m6A-mRNA&lncRNA Epitranscriptomic Microarray data and RNA sequencing used in this study data have been deposited in Gene Expression Omnibus database under accession code GSE241195 (https://www.ncbi.nlm.nih.gov/geo/query/acc.cgi?acc=GSE241195) and GSE241413 (https://www.ncbi.nlm.nih.gov/geo/query/acc.cgi?acc=GSE241413), respectively. The ChEA3 database are available at https://maayanlab.cloud/chea3/. The ENCORI database are available at https://starbase.sysu.edu.cn/index.php. The mRNA m6A site was predicted by the online tool SRAMP at http://www.cuilab.cn/sramp/. Source data are provided with this paper.

Research involving human participants, their data, or biological material

Policy information about studies with human participants or human data. See also policy information about sex, gender (identity/presentation), and sexual orientation and race, ethnicity and racism.

Reporting on sex and gender	In the last 45 years (1973–2018), global semen quality has decreased by more than 62%. Globally, the number of obese and overweight people are on the rise. However, the effect of overweight/obesity on sperm m6A levels remain unknown. A case-control study containing 30 pairs were obtained to explore the effects. Our results suggest that elevated sperm m6A level and decreased sperm concentration is observed in overweight/obesity donors.
Reporting on race, ethnicity, or other socially relevant groupings	The study did not address race, ethnicity, or other socially relevant grouping.
Population characteristics	To investigate the effect of overweight/obesity on sperm m6A levels, a case-control study was established. A total of 428 human sperm were obtained from the reproductive medicine center of the First Affiliated Hospital of Anhui Medical University with the donor’s informed consent. After removal of smoking or alcohol drinking donors, 168 sperm samples were available. Finally, 30 pairs of overweight/obesity cases and corresponding controls were obtained by matching age (20-40 age).
Recruitment	A total of 428 human sperm were obtained from the reproductive Center of the First Affiliated Hospital of Anhui Medical University with the donor’s informed consent.
Ethics oversight	Approved by the Clinical Medical Research Ethics Committee of the First Affiliated Hospital of Anhui Medical University (ethical approval number: PJ2023-04-12)

Note that full information on the approval of the study protocol must also be provided in the manuscript.

Field-specific reporting

Please select the one below that is the best fit for your research. If you are not sure, read the appropriate sections before making your selection.

[x] Life sciences   [ ] Behavioural & social sciences   [ ] Ecological, evolutionary & environmental sciences

For a reference copy of the document with all sections, see nature.com/documents/nr-reporting-summary-flat.pdf

Life sciences study design

All studies must disclose on these points even when the disclosure is negative.

Sample size	No sample-size calculation was performed. Based on community standards, n=6 or more mice per group was acceptable for animal studies (Nat Commun. 2023; 14(1): 2656.). In case-control study, sample size was determined according to the previous experimental observations and guidance in the field (Hum Reprod. 2018; 33(4): 553-562.).
Data exclusions	In animal experiments, to ensure the development of the pups, the balances of sexes between pups (male: female, 3: 3) were performed on postnatal day (PND) 1. In case-control study, we removed smoking and alcohol donors because they are risk factors for low semen quality.
Replication	In this study, all results were replicated at least 4-6 times, and there was little individual difference within the group.
Randomization	In animal experiments, mice were randomly assigned to each treatment group based on body weight. In case-control study, a total of 428 human sperm were obtained from the reproductive medicine center of the First Affiliated Hospital of Anhui Medical University with the donor’s informed consent. After removal of smoking or alcohol drinking donors, 168 sperm samples were available. Finally, 30 pairs of overweight/obesity cases and corresponding controls were obtained by matching age.
Blinding	The investigators were blinded to group allocation during data collection and analysis.

Reporting for specific materials, systems and methods

We require information from authors about some types of materials, experimental systems and methods used in many studies. Here, indicate whether each material, system or method listed is relevant to your study. If you are not sure if a list item applies to your research, read the appropriate section before selecting a response.

Materials & experimental systems

n/a	Involved in the study
☐	☒ Antibodies
☐	☒ Eukaryotic cell lines
☒	☐ Palaeontology and archaeology
☐	☒ Animals and other organisms
☒	☐ Clinical data
☒	☐ Dual use research of concern
☒	☐ Plants

Methods

n/a	Involved in the study
☒	☐ ChIP-seq
☒	☐ Flow cytometry
☒	☐ MRI-based neuroimaging

Antibodies

Antibodies used

(1) Rabbit anti-DDX4, Abcam, ab13840, 1:1000;
(2) Mouse anti-PLZF, Santa Cruz Biotechnologies, sc-28319, 1:200;
(3) Mouse anti-C-KIT, Santa Cruz Biotechnologies, sc-365504, 1:200;
(4) Mouse anti- SYCP3, Santa Cruz Biotechnologies, sc-74569, 1:200;
(5) Rabbit anti-RBP4, Abcam, ab188230, 1:1000;
(6) Rabbit anti-RARα, Cell Signaling Technology, 62294S, 1:1000;
(7) Rabbit anti-STRA8, Abcam, ab49602, 1:1000;
(8) Rabbit anti-ALDH1A1, Abcam, ab52492, 1:1000;
(9) Rabbit anti-ALDH1A2, Cell Signaling Technology, 83805S, 1:1000;
(10) Rabbit anti-WT1, Abcam, ab89901, 1:1000;
(11) Rabbit anti-METTL3, Abcam, ab195352, 1:2000;
(12) Rabbit anti-METTL14, Cell Signaling Technology, 51104S, 1:1000;
(13) Rabbit anti-ALKBH5, Abcam, ab195377, 1:1000;
(14) Mouse anti-FTO, Abcam, ab92821, 1:1000;
(15) Rabbit anti-YTHDF1, Proteintech, 17479-1-AP, 1:1000;
(16) Rabbit anti-YTHDF2, Proteintech, 24744-1-AP, 1:1000;
(17) Rabbit anti-IGF2BP1, Proteintech, 22803-1-AP, 1:1000;
(18) Rabbit anti-β-Actin, Sigma-Aldrich, A1978, 1:10000;
(19) CyTM3 affinipure donkey anti-rabbit IgG (H+L), Jackson ImmunoResearch, 711-165-152, 1:500;
(20) Alexa Fluor® 488 affinipure donkey anti-rabbit IgG (H+L), Jackson ImmunoResearch, 11-545-152, 1:500.

Validation

All the primary antibodies in this paper were obtained from the manufacturer with validation and citations.
Validation of the antibodies used in this study:
(1) Rabbit anti-DDX4, Abcam, ab13840. REF: PMID: 34747116. https://www.abcam.cn/products/primary-antibodies/ddx4--mvh-antibody-ab13840.html.
(2) Mouse anti-PLZF, Santa Cruz Biotechnologies, sc-28319. REF: PMID: 10611298. https://www.scbt.com/p/plzf-antibody-d-9?requestFrom=search.
(3) Mouse anti-C-KIT, Santa Cruz Biotechnologies, sc-365504. REF: PMID: 1371359. https://www.scbt.com/p/c-kit-antibody-e-3?requestFrom=search.
(4) Mouse anti- SYCP3, Santa Cruz Biotechnologies, sc-74569. REF: PMID: 11483963. https://www.scbt.com/p/scp-3-antibody-d-1?requestFrom=search.
(5) Rabbit anti-RBP4, Abcam, ab188230. REF: PMID: 35830795. https://www.abcam.cn/products/primary-antibodies/rbp4-antibody-epr18020-115-ab188230.html.
(6) Rabbit anti-RARα, Cell Signaling Technology, 62294S. REF: PMID: 36031407. https://www.cellsignal.cn/products/primary-antibodies/rara-e6z6k-rabbit-mab/62294.
(7) Rabbit anti-STRA8, Abcam, ab49602. REF: PMID: 33603791. https://www.abcam.cn/products/primary-antibodies/stra8-antibody-ab49602.html.
(8) Rabbit anti-ALDH1A1, Abcam, ab52492. REF: PMID: 35318302. https://www.abcam.cn/products/primary-antibodies/aldh1a1-antibody-ep1933y-c-terminal-ab52492.html.
(9) Rabbit anti-ALDH1A2, Cell Signaling Technology, 83805S. REF: PMID: 34994166. https://www.cellsignal.cn/products/primary-antibodies/aldh1a2-antibody/83805.
(10) Rabbit anti-WT1, Abcam, ab89901. REF: PMID: 35396346. https://www.abcam.cn/products/primary-antibodies/wilms-tumor-protein-antibody-can-r9ihc-56-2-ab89901.html.
(11) Rabbit anti-METTL3, Abcam, ab195352. REF: PMID: 36719213. https://www.abcam.cn/products/primary-antibodies/mettl3-antibody-epr18810-ab195352.html.
(12) Rabbit anti-METTL14, Cell Signaling Technology, 51104S. REF: PMID: 36094741. https://www.cellsignal.cn/products/primary-antibodies/mettl14-d8k8w-rabbit-mab/51104.

(13) Rabbit anti-ALKBH5, Abcam, ab195377. REF: PMID: 37095108. https://www.abcam.cn/products/primary-antibodies/alkbh5-antibody-epr18958-ab195377.html.
(14) Mouse anti-FTO, Abcam, ab92821. REF: PMID: 35098923. https://www.abcam.cn/products/primary-antibodies/fto-antibody-5-2h10-ab92821.html.
(15) Rabbit anti-YTHDF1, Proteintech, 17479-1-AP. https://www.ptgcn.com/products/YTHDF1-Antibody-17479-1-AP.htm
(16) Rabbit anti-YTHDF2, Proteintech, 24744-1-AP. https://www.ptgcn.com/products/YTHDF2-Antibody-24744-1-AP.htm
(17) Rabbit anti-IGF2BP1, Proteintech, 22803-1-AP. https://www.ptgcn.com/products/IGF2BP1-Antibody-22803-1-AP.htm
(18) Rabbit anti-β-Actin, Sigma-Aldrich, A1978. https://www.sigmaaldrich.cn/CN/zh/search/a1978?focus=products&page=1&perpage=30&sort=relevance&term=a1978&type=product

Eukaryotic cell lines

Policy information about cell lines and Sex and Gender in Research

Cell line source(s)	TM4 cells, the normal mouse testicular Sertoli cell lines, were from the Cell Bank of the Chinese Academy of Sciences
Authentication	The cell line was validated based on the morphology and STR profiling before use.
Mycoplasma contamination	The cell lines were not tested for mycoplasma contamination
Commonly misidentified lines (See ICLAC register)	No misidentified cell lines

Animals and other research organisms

Policy information about studies involving animals; ARRIVE guidelines recommended for reporting animal research, and Sex and Gender in Research

Laboratory animals	The C57BL/6N mice were provided by Beijing Vital River Laboratory Animal Technology Co., Ltd (Beijing, China). All animals (4 weeks old) were accustomed to standard conditions for a week. The purified control feed (TP23302) and 60% fat high-fat feed (TP23300) were acquired from Trophic Animal Feed High-Tech Co., Ltd (Nantong, China). In the different generations, 8 to 11 pregnant mice were obtained in each group after mating with different male mice, and the balances of sexes between pups (male: female, 3: 3) were performed on postnatal day (PND) 1. At PND28, one male mouse from each litter was selected. For euthanasia, all mice were injected intraperitoneally with 2,2,2-tribromoe-thanol (250 mg/kg), and then cervical dislocation was executed under anesthesia. This study was approved by the Laboratory Animal Ethics Committee of Anhui Medical University (ethical approval number: LLSC20220640).
Wild animals	The study did not involve wild animals.
Reporting on sex	In the last 45 years (1973–2018), global semen quality has decreased by more than 62%. Globally, the number of obese and overweight people are on the rise.However, the effects of multigenerational paternal obesity on the susceptibility to spermatogenesis disorders in offspring remain unknown. We aimed to investigate this effect and its m6A-dependent mechanism using a mouse model for multigenerational paternal high-fat diet (HFD) combined with offspring cadmium (Cd, a well-known reproductive toxicant) treatment. As a result, sperm count, the expression of differentiating-spermatogonia and subsequent germ cell marker, and retinoic acid (RA) levels were gradually reduced in offspring with the increase of HFD generation.
Field-collected samples	This study did not involve samples collected from the field.
Ethics oversight	This study was approved by the Laboratory Animal Ethics Committee of Anhui Medical University (ethical approval number: LLSC20220640)

Note that full information on the approval of the study protocol must also be provided in the manuscript.
