# Supplementary material for: Multigenerational paternal obesity enhances the susceptibility to male subfertility in offspring via Wt1 N6-methyladenosine modification
Source: Nat Commun. 2024 Feb 14;15:1353. doi: 10.1038/s41467-024-45675-4 (PMC10866985; doi:10.1038/s41467-024-45675-4)

Figure 2b

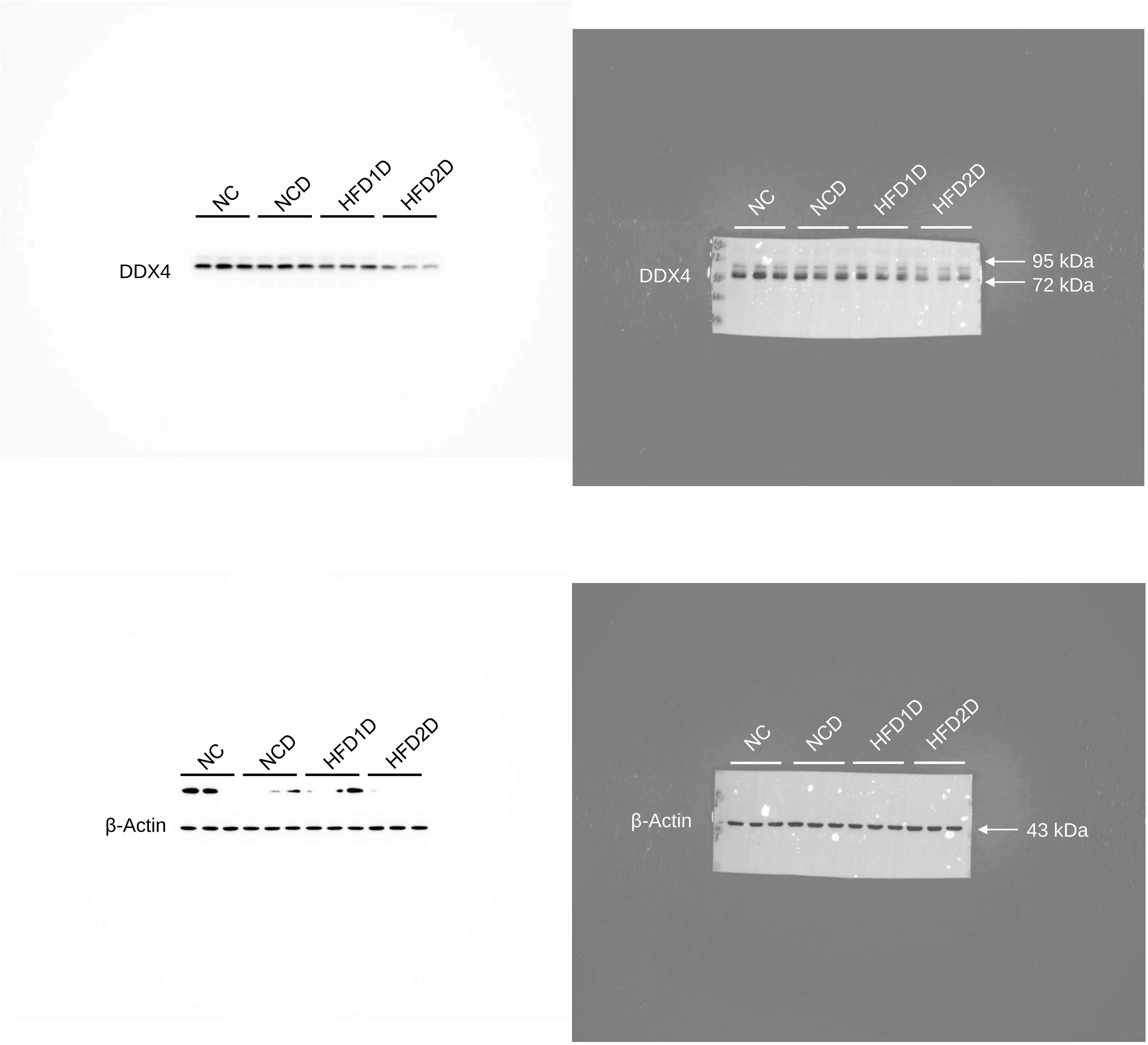
NC
NCD
HFD1D
HFD2D
DDX4
NC
NCD
HFD1D
HFD2D
DDX4
95 kDa
72 kDa
NC
NCD
HFD1D
HFD2D
β-Actin
NC
NCD
HFD1D
HFD2D
β-Actin
43 kDa

Figure 2e

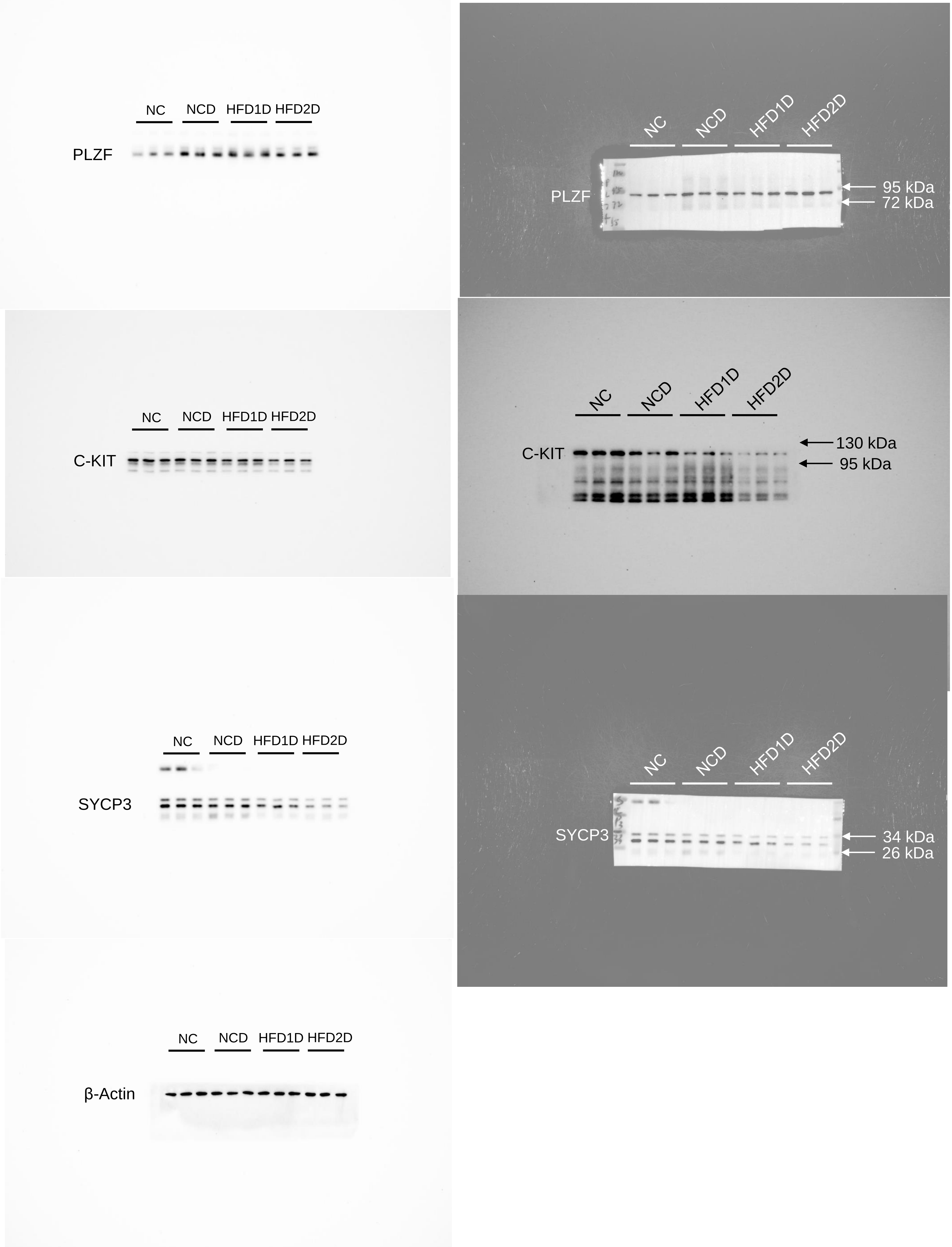
NC
NCD
HFD1D
HFD2D
PLZF
95 kDa
72 kDa
C-KIT
130 kDa
95 kDa
SYCP3
34 kDa
26 kDa
β-Actin

Figure 3d

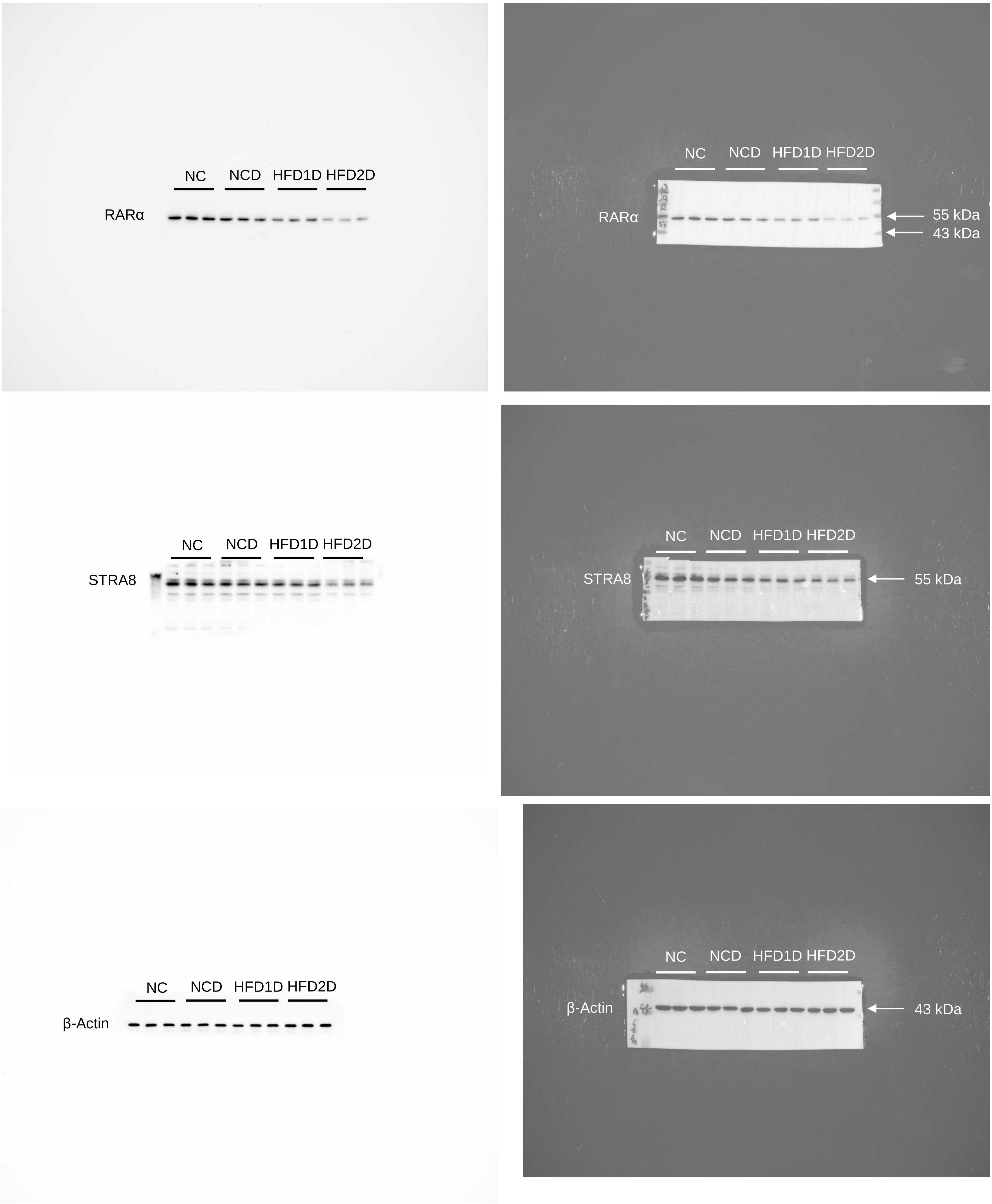
NC
NCD
HFD1D
HFD2D
RARα
55 kDa
43 kDa
STRA8
55 kDa
β-Actin
43 kDa

Figure 3g

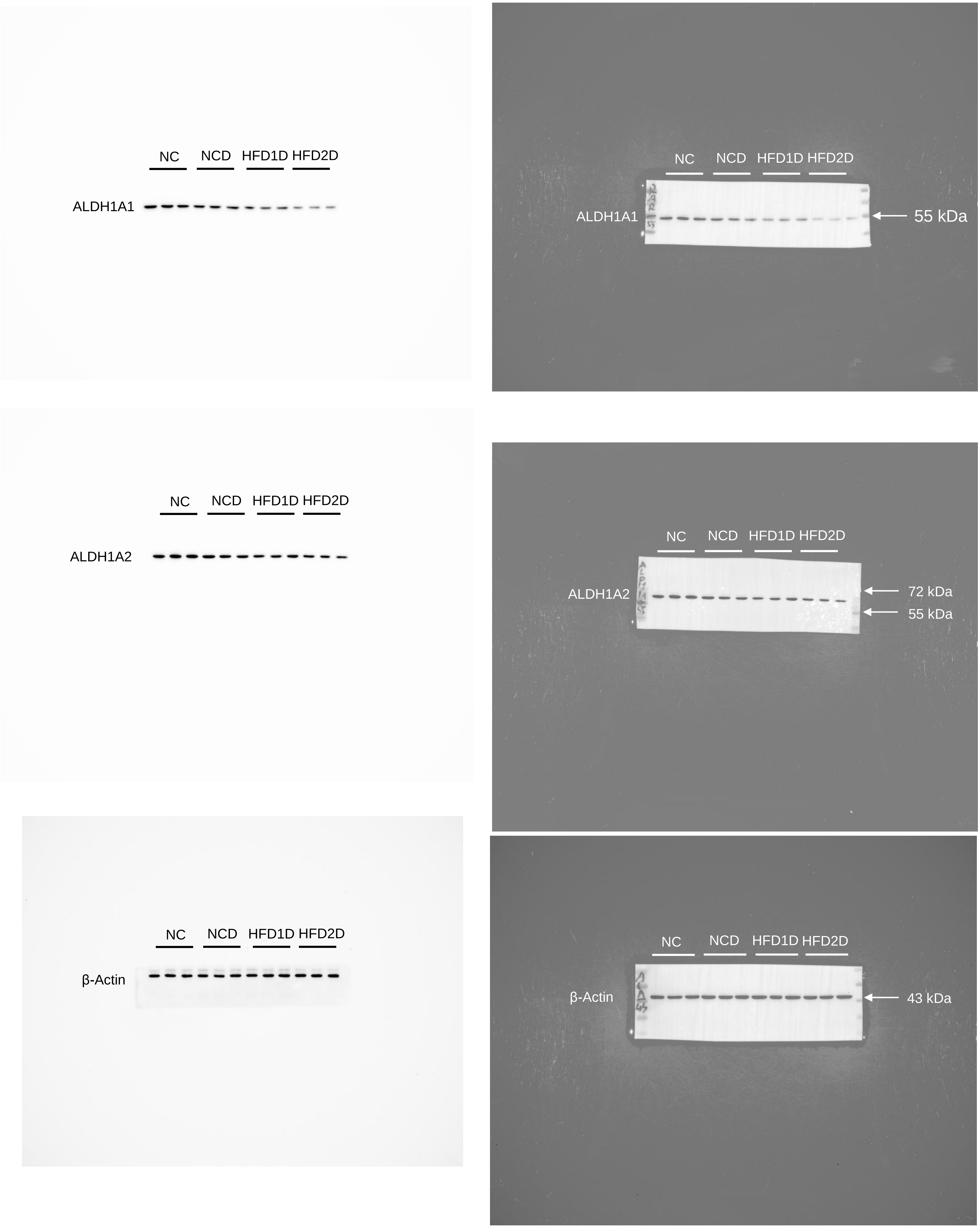

NC
NCD
HFD1D
HFD2D
ALDH1A1
NC
NCD
HFD1D
HFD2D
ALDH1A1
55 kDa
NC
NCD
HFD1D
HFD2D
ALDH1A2
NC
NCD
HFD1D
HFD2D
ALDH1A2
72 kDa
55 kDa
NC
NCD
HFD1D
HFD2D
β-Actin
NC
NCD
HFD1D
HFD2D
β-Actin
43 kDa

Figure 4c

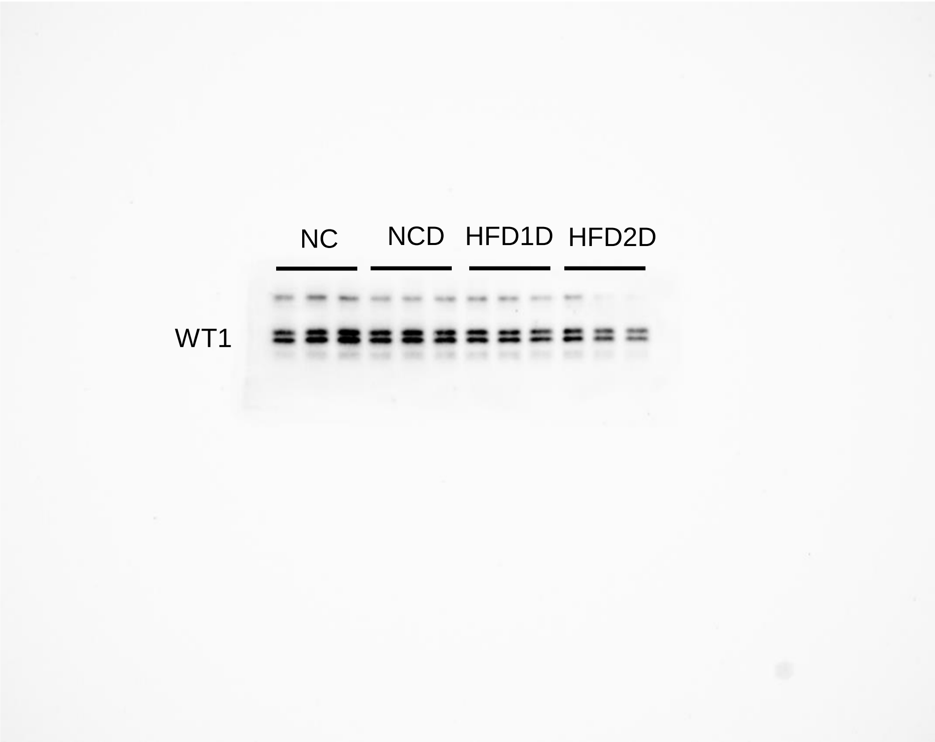

NC
NCD
HFD1D
HFD2D
WT1

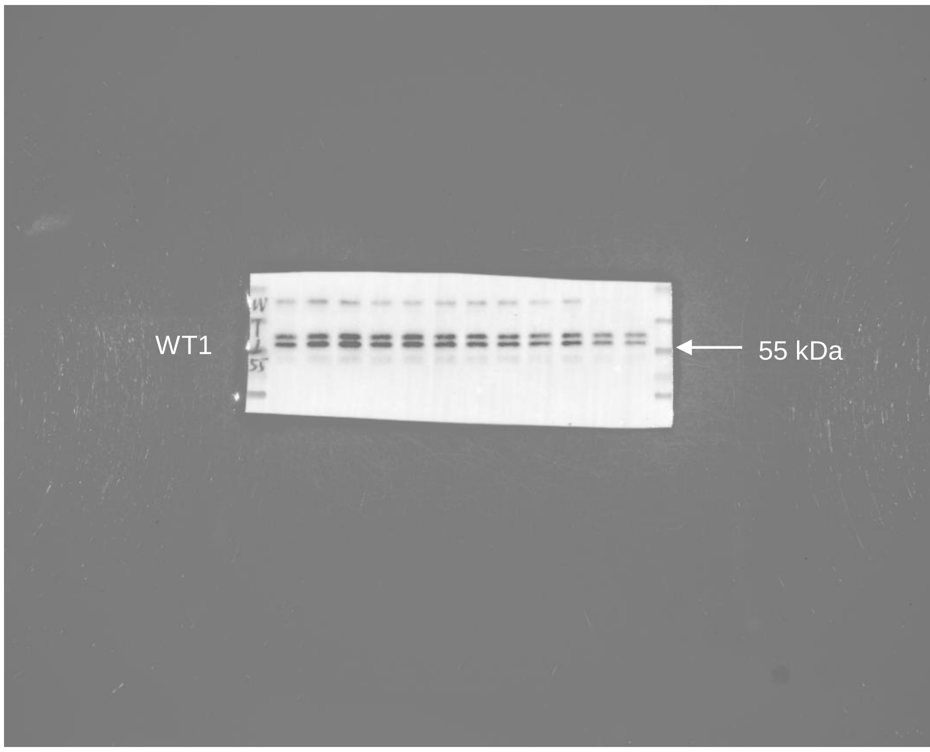

WT1
55 kDa

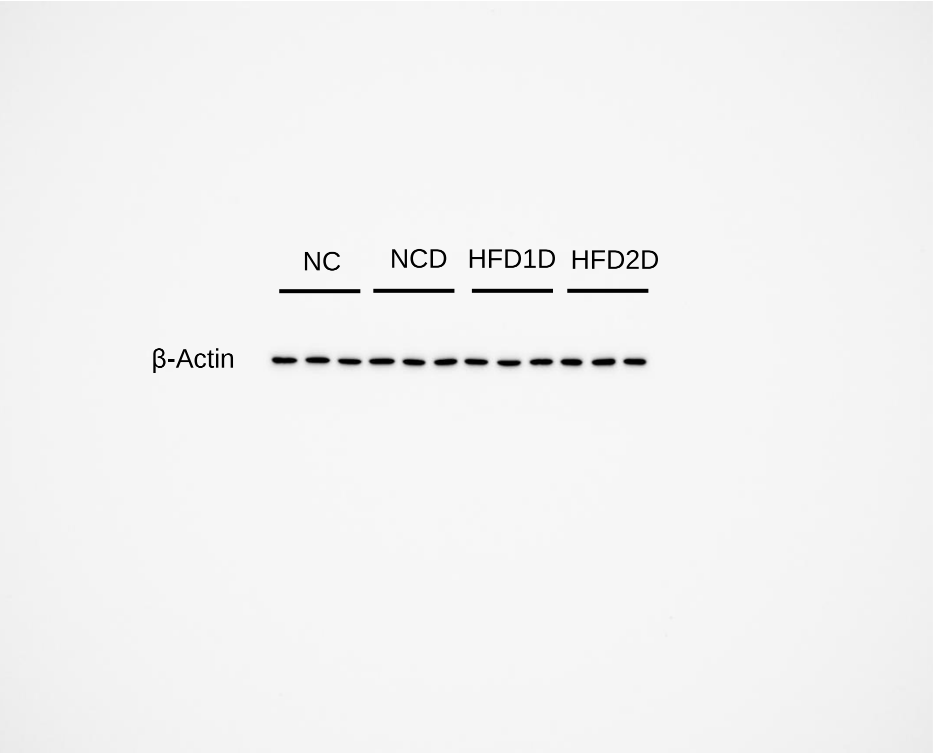

NC
NCD
HFD1D
HFD2D
β-Actin

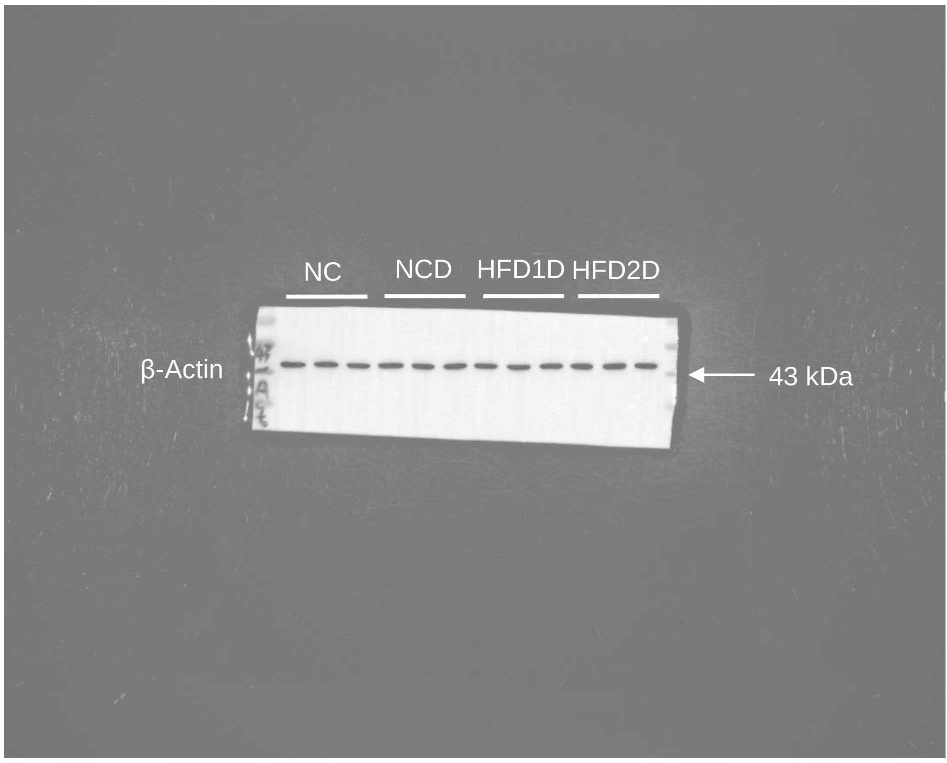

NC
NCD
HFD1D
HFD2D
β-Actin
43 kDa

Figure 5d

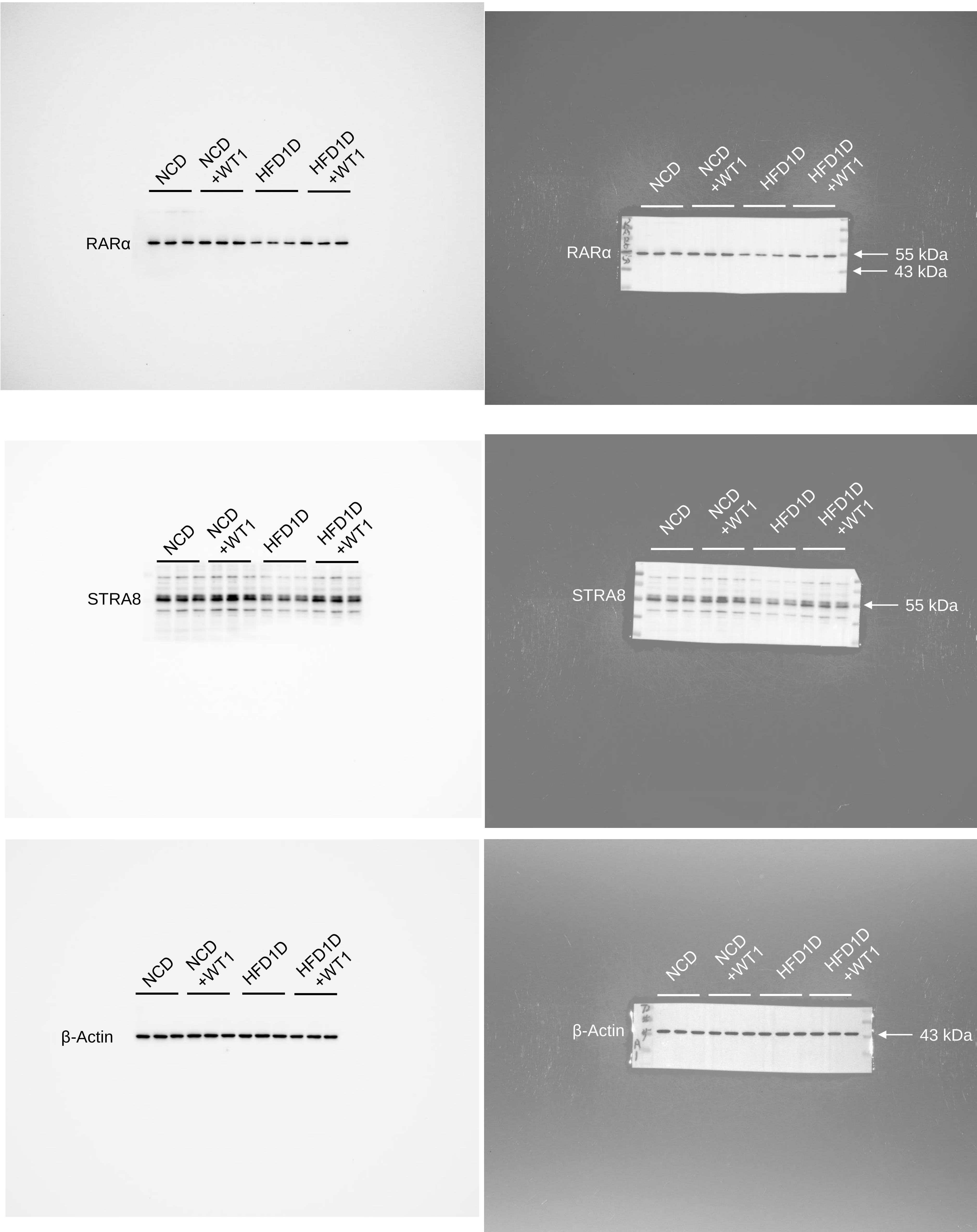
NCD
NCD +WT1
HFD1D
HFD1D +WT1
RARα
55 kDa
43 kDa
STRA8
55 kDa
β-Actin
43 kDa

Figure 5h

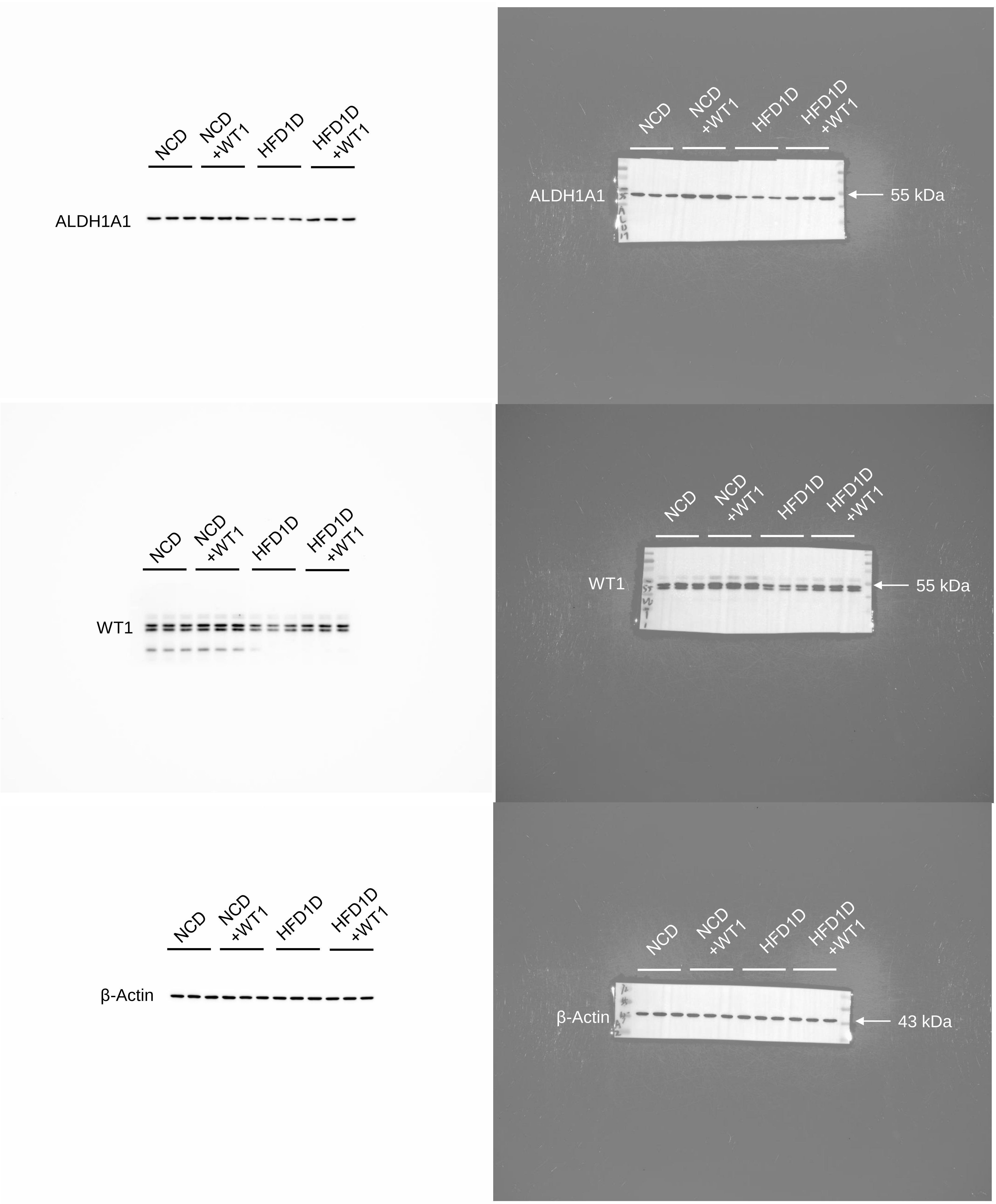
NCD
NCD +WT1
HFD1D
HFD1D +WT1
ALDH1A1
55 kDa
WT1
55 kDa
β-Actin
43 kDa

Figure 6b

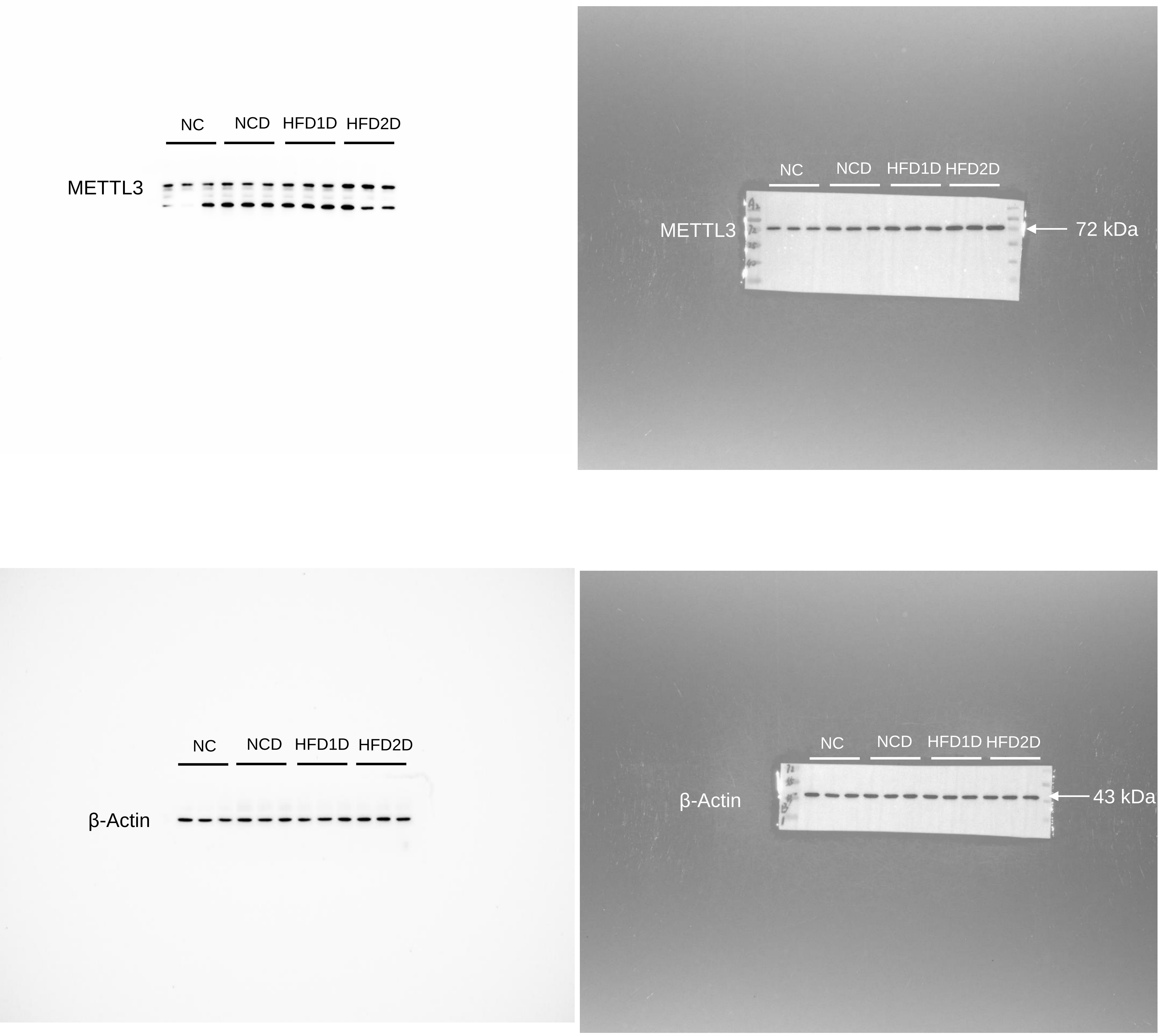
NC
NCD
HFD1D
HFD2D
METTL3
NC
NCD
HFD1D
HFD2D
METTL3
72 kDa
NC
NCD
HFD1D
HFD2D
β-Actin
NC
NCD
HFD1D
HFD2D
β-Actin
43 kDa

Figure 6e

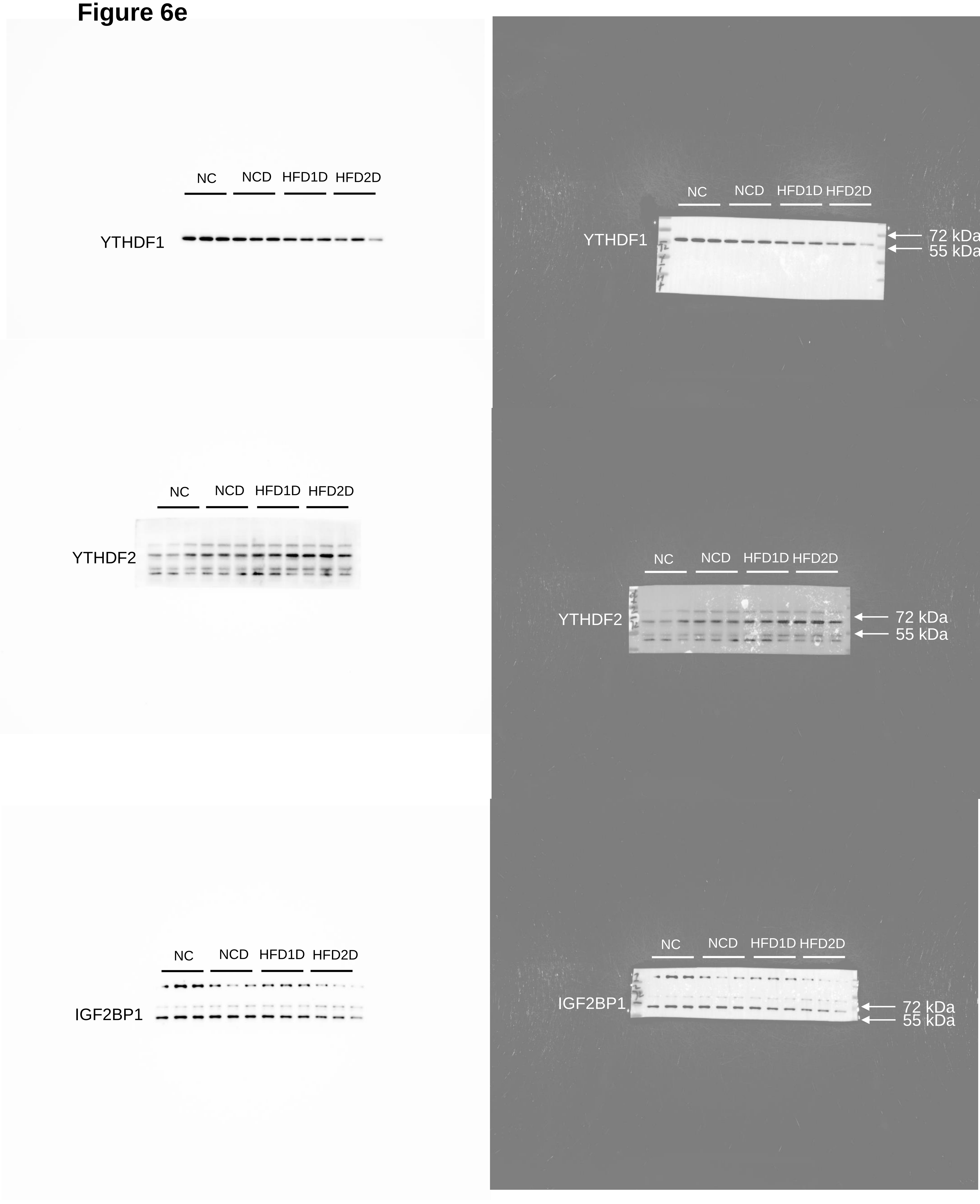
NC
NCD
HFD1D
HFD2D
YTHDF1
NC
NCD
HFD1D
HFD2D
YTHDF1
72 kDa
55 kDa
NC
NCD
HFD1D
HFD2D
YTHDF2
NC
NCD
HFD1D
HFD2D
YTHDF2
72 kDa
55 kDa
NC
NCD
HFD1D
HFD2D
IGF2BP1
NC
NCD
HFD1D
HFD2D
IGF2BP1
72 kDa
55 kDa

Figure. 7g

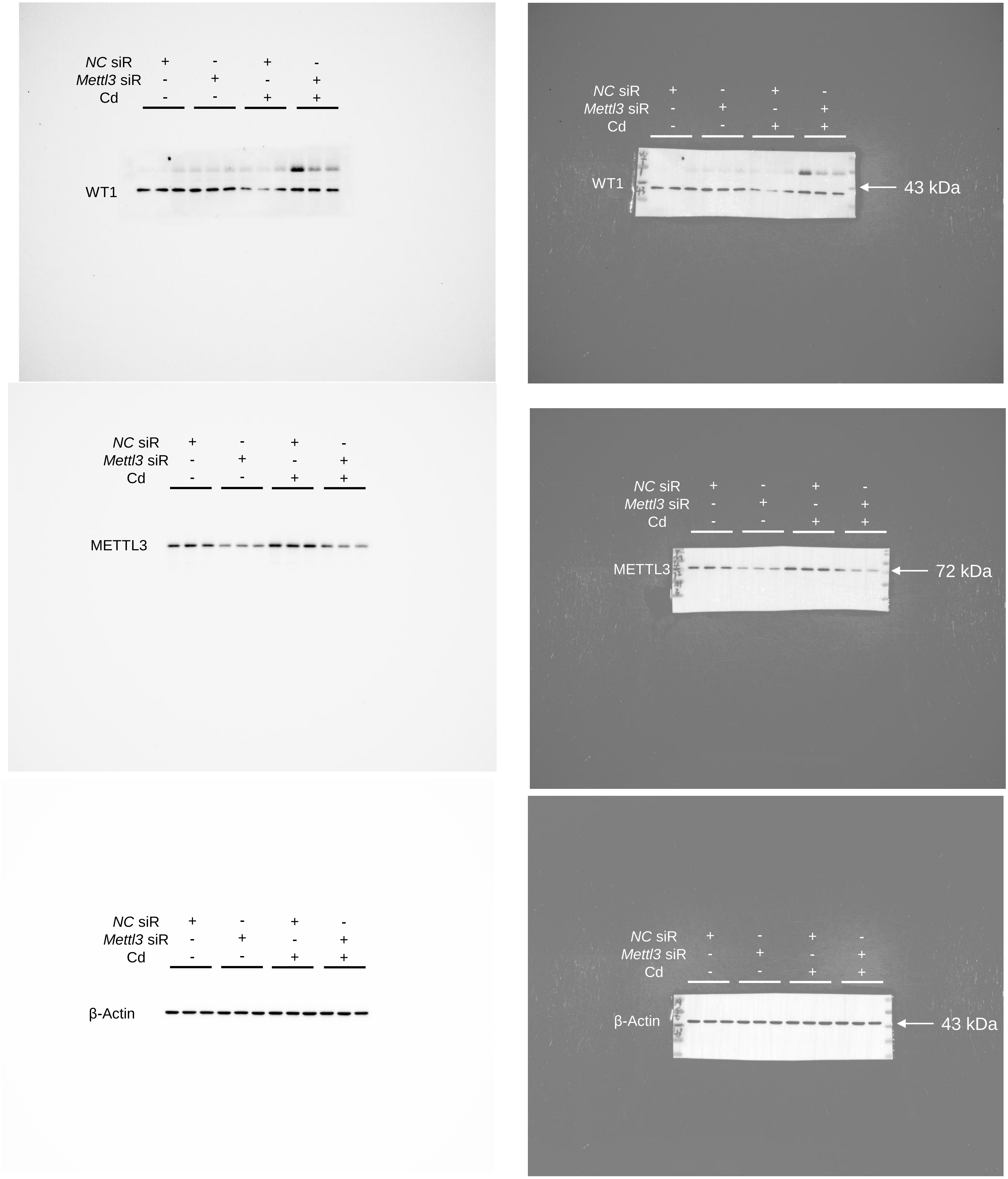

Figure. 7j

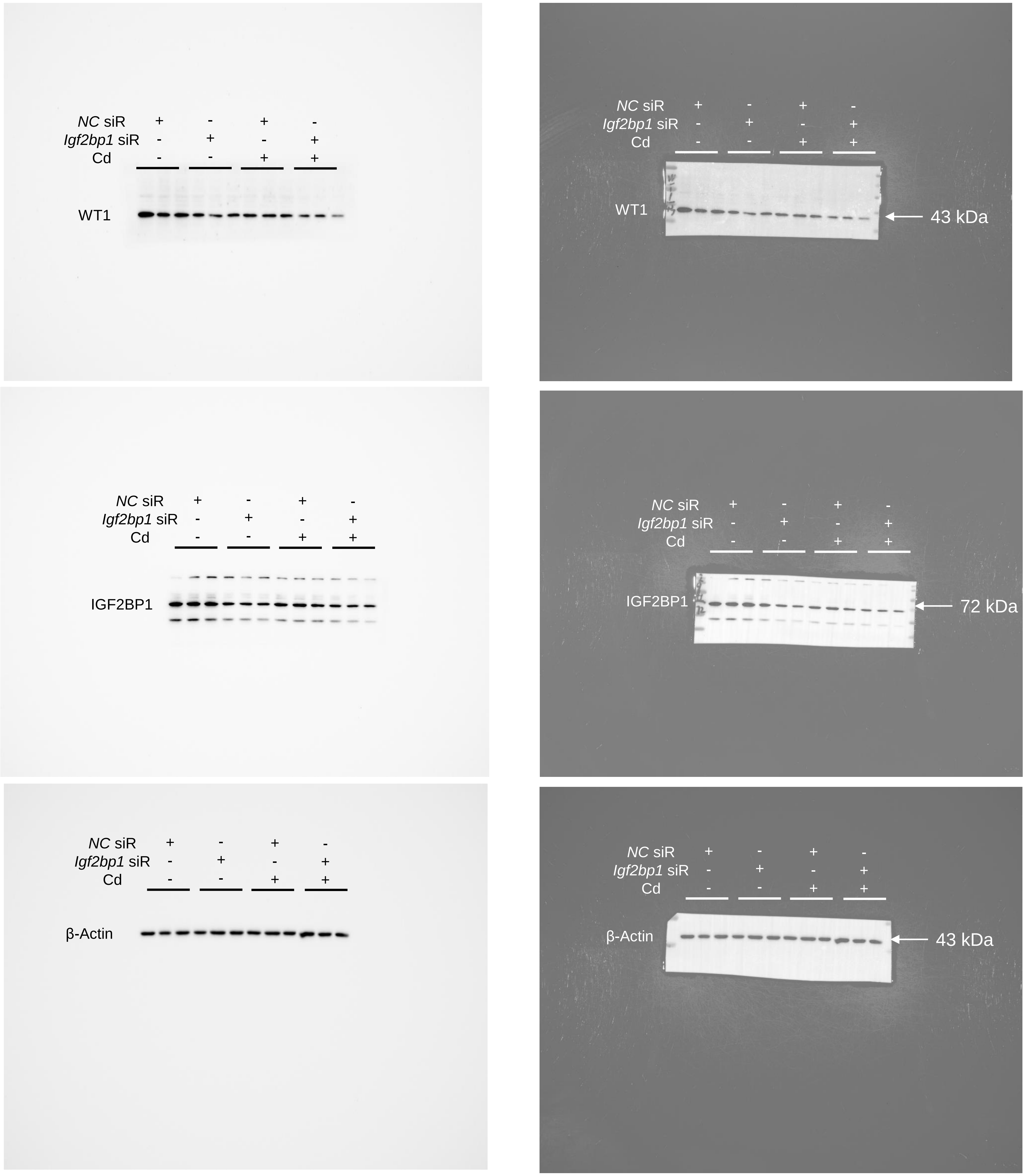
NC siR + - + -
Igf2bp1 siR - + - +
Cd - - + +
WT1
NC siR + - + -
Igf2bp1 siR - + - +
Cd - - + +
WT1
43 kDa
NC siR + - + -
Igf2bp1 siR - + - +
Cd - - + +
IGF2BP1
NC siR + - + -
Igf2bp1 siR - + - +
Cd - - + +
IGF2BP1
72 kDa
NC siR + - + -
Igf2bp1 siR - + - +
Cd - - + +
β-Actin
NC siR + - + -
Igf2bp1 siR - + - +
Cd - - + +
β-Actin
43 kDa

Figure 9d

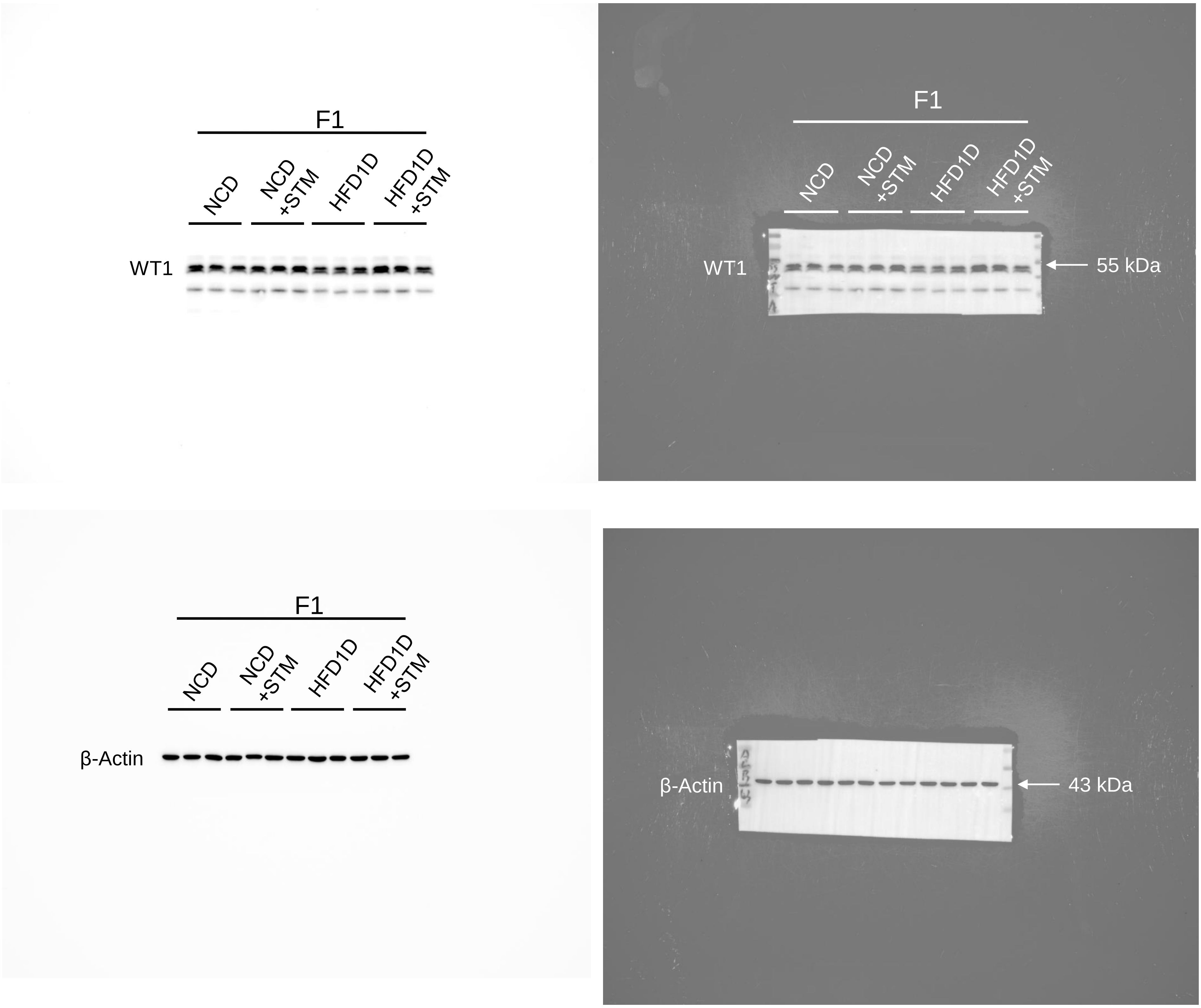
F1
NCD
NCD +STM
HFD1D
HFD1D +STM
WT1
F1
NCD
NCD +STM
HFD1D
HFD1D +STM
WT1
55 kDa
F1
NCD
NCD +STM
HFD1D
HFD1D +STM
β-Actin
β-Actin
43 kDa

Figure 9h

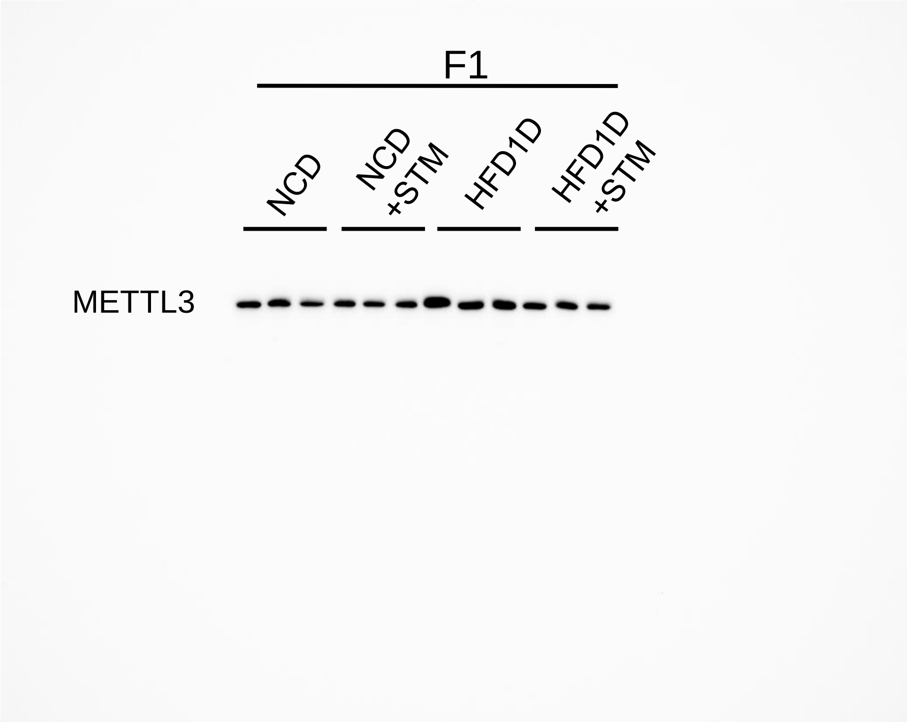
F1
NCD
NCD +STM
HFD1D
HFD1D +STM
METTL3

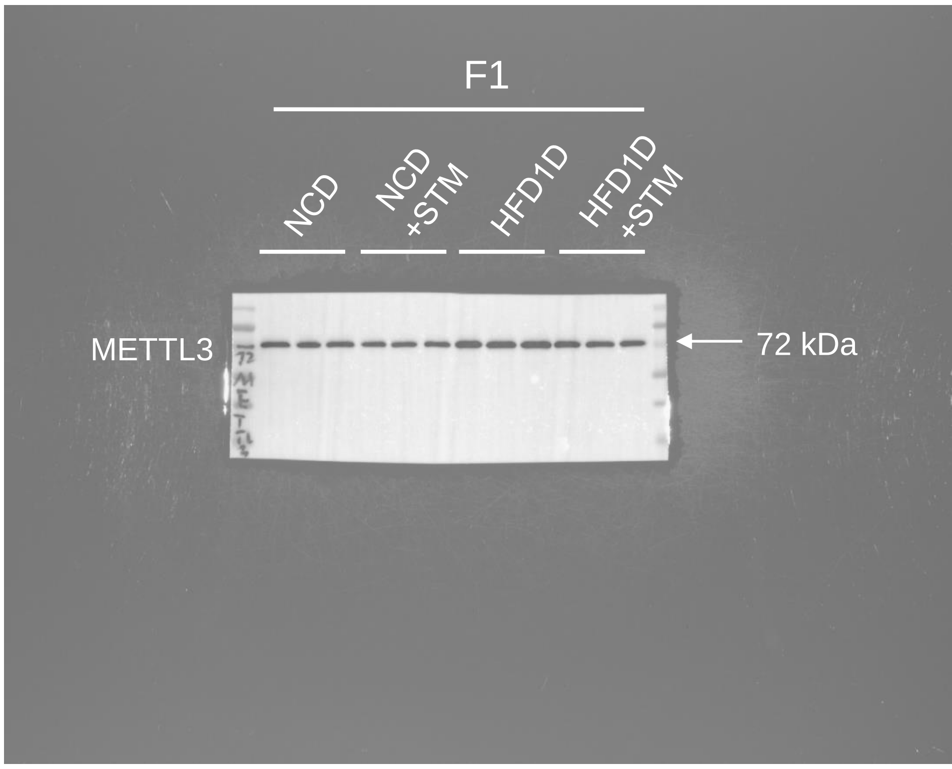
F1
NCD
NCD +STM
HFD1D
HFD1D +STM
METTL3
72 kDa

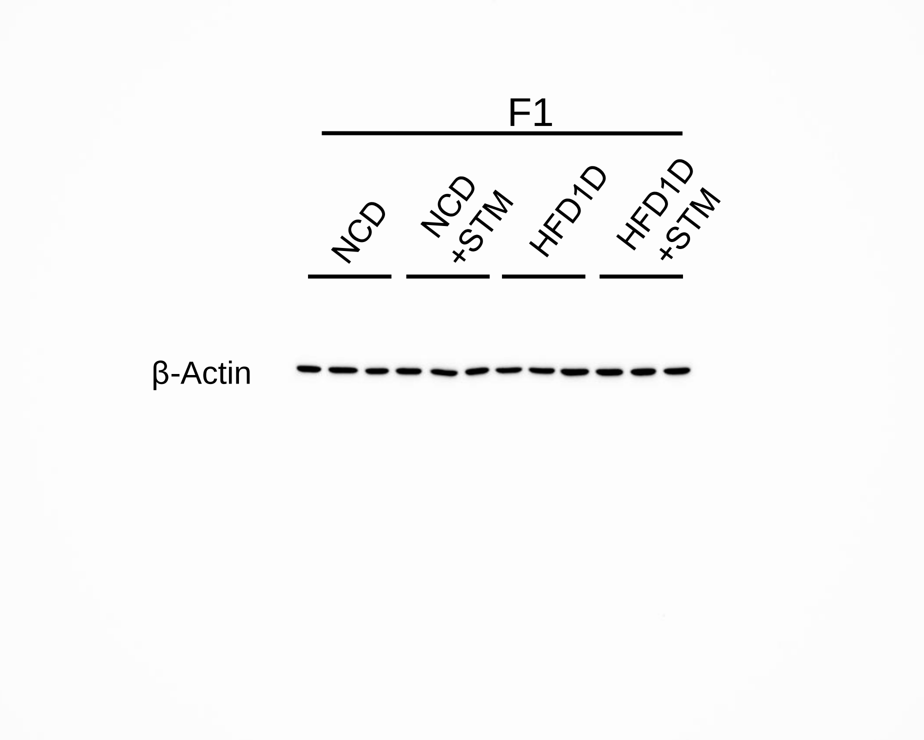
F1
NCD
NCD +STM
HFD1D
HFD1D +STM
β-Actin

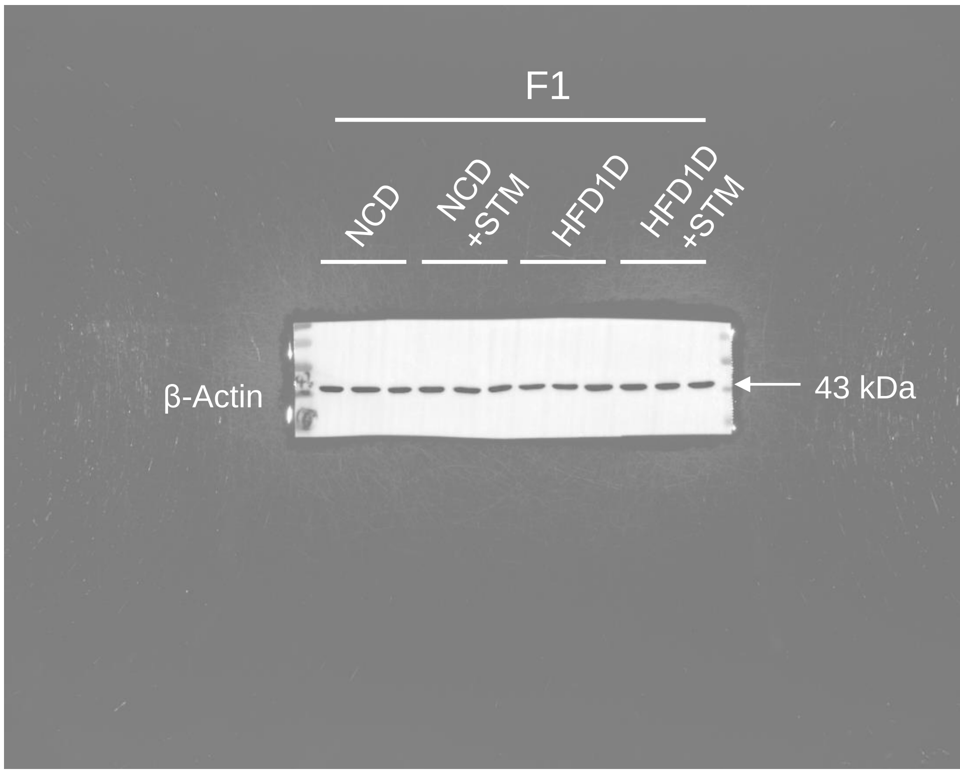
F1
NCD
NCD +STM
HFD1D
HFD1D +STM
β-Actin
43 kDa

Supplementary Figure 4b

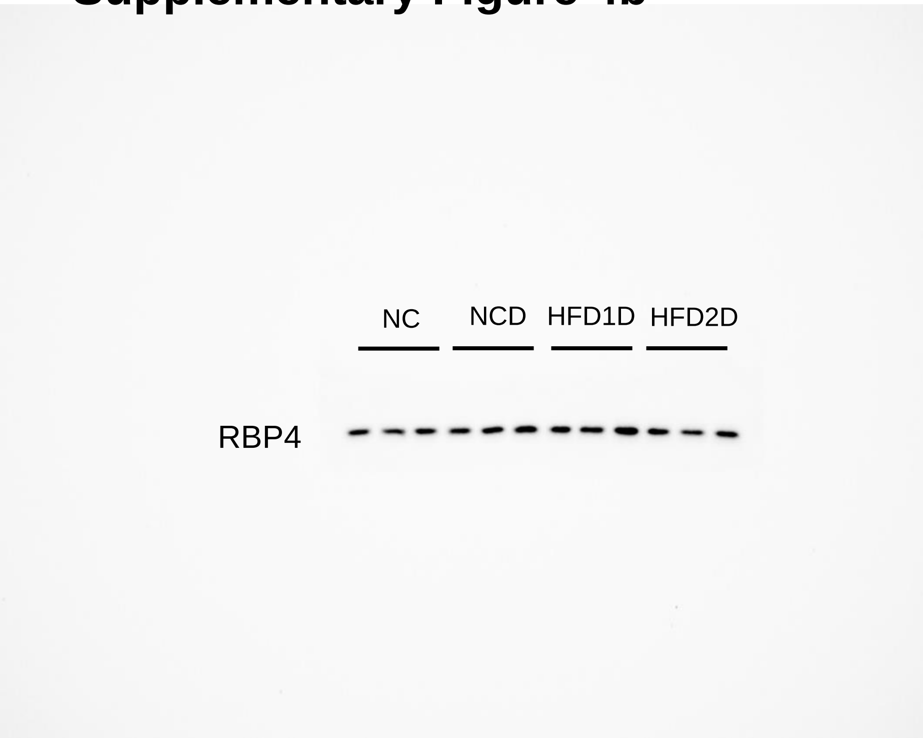

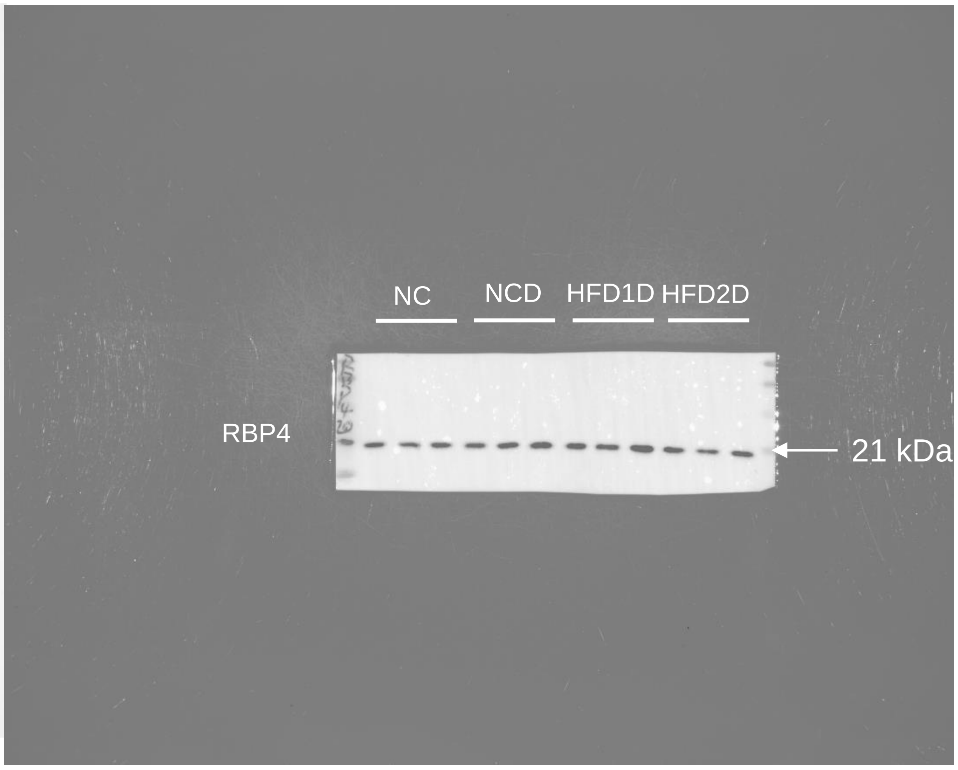

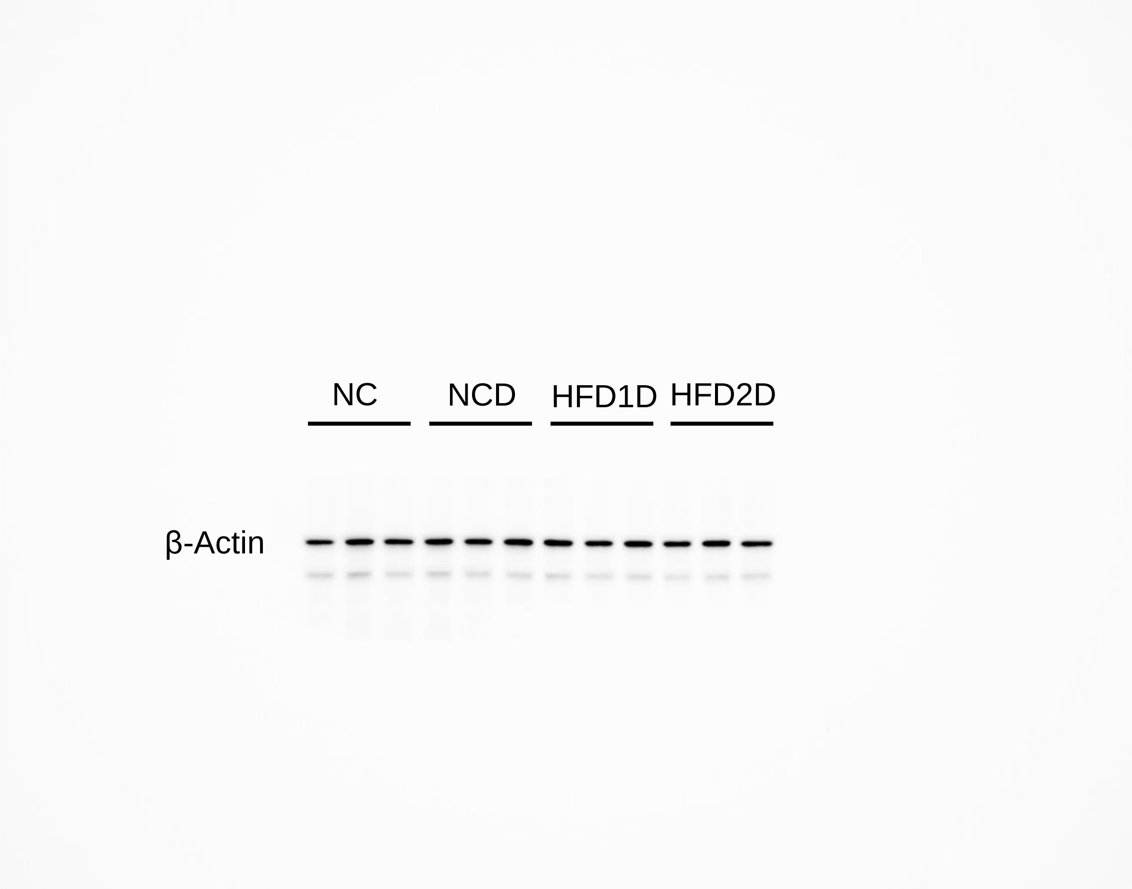

Supplementary Figure 5a

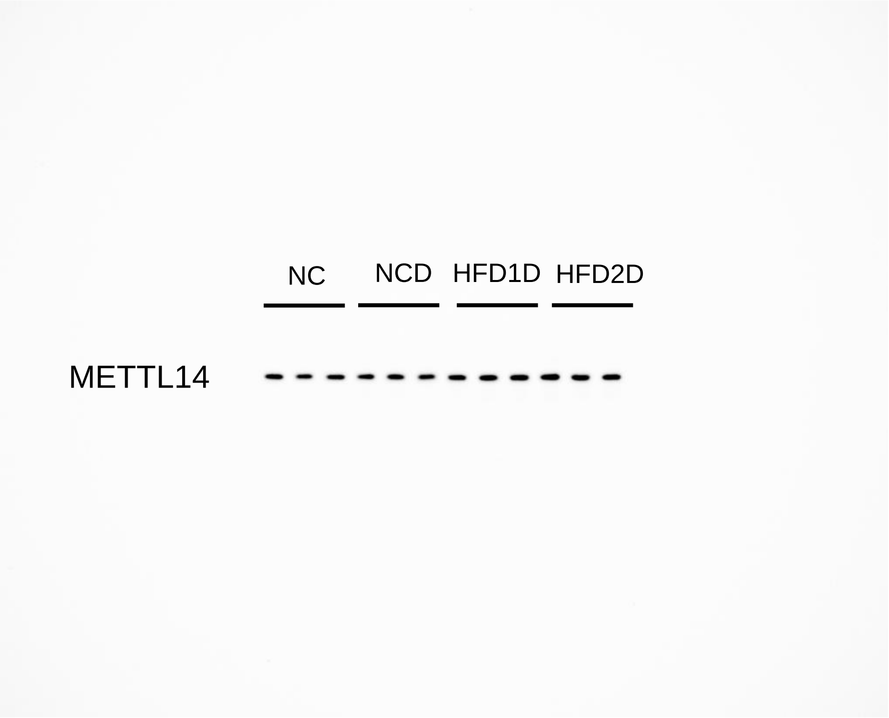

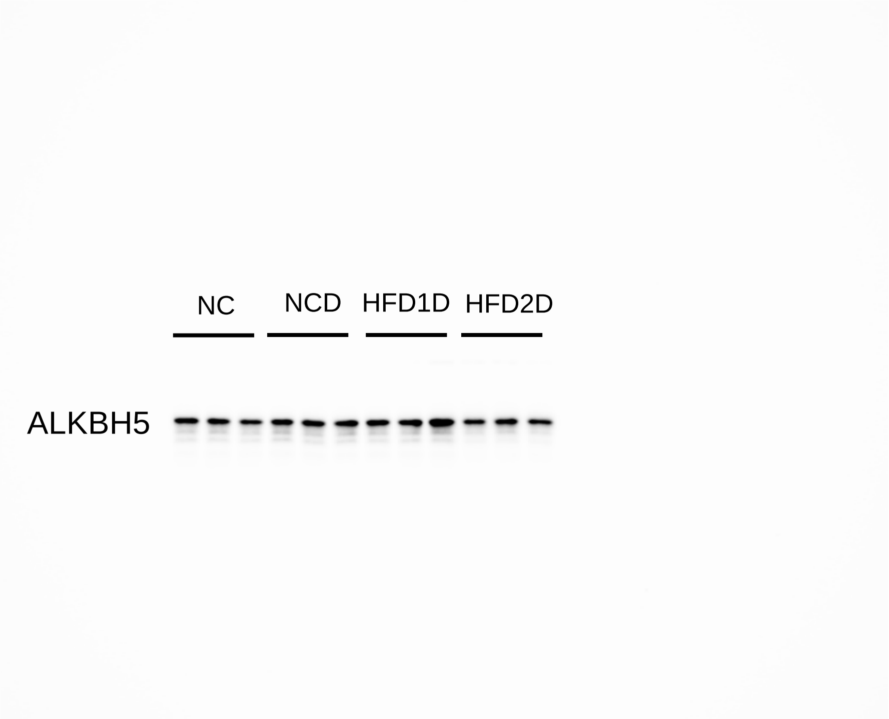

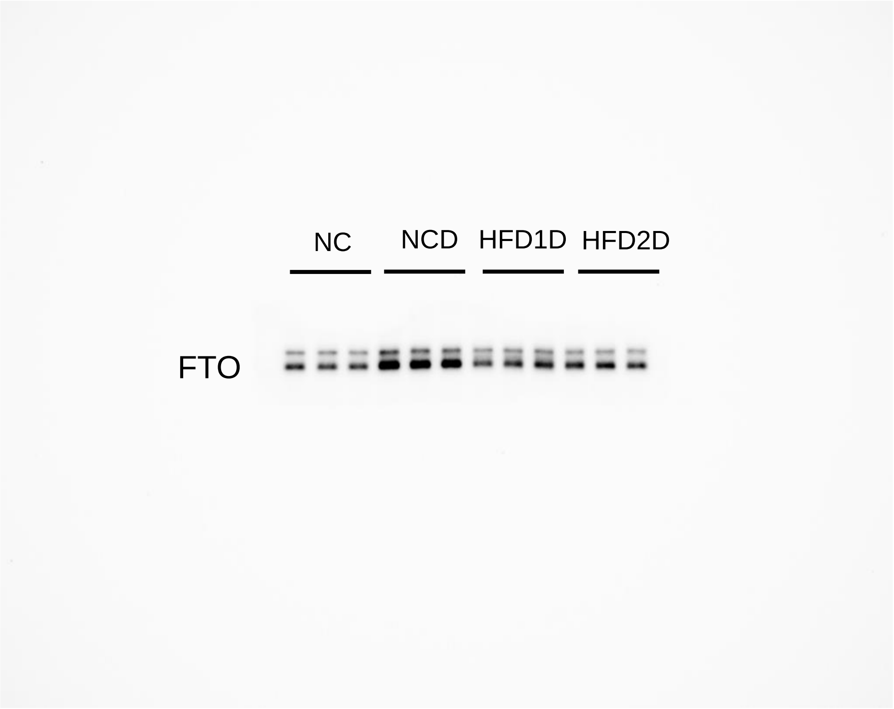

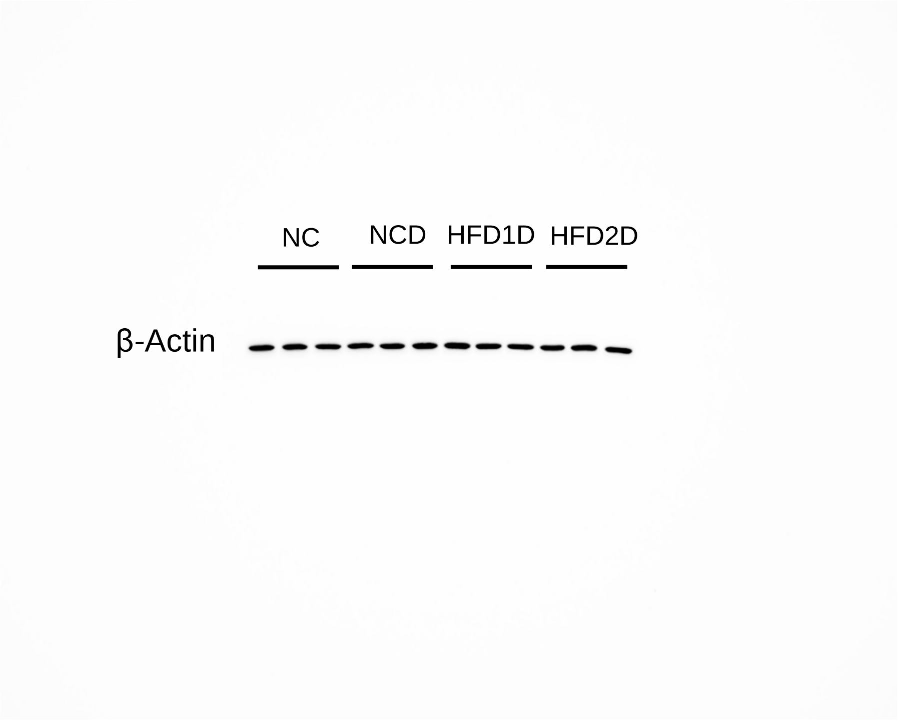

Supplementary Figure 7c

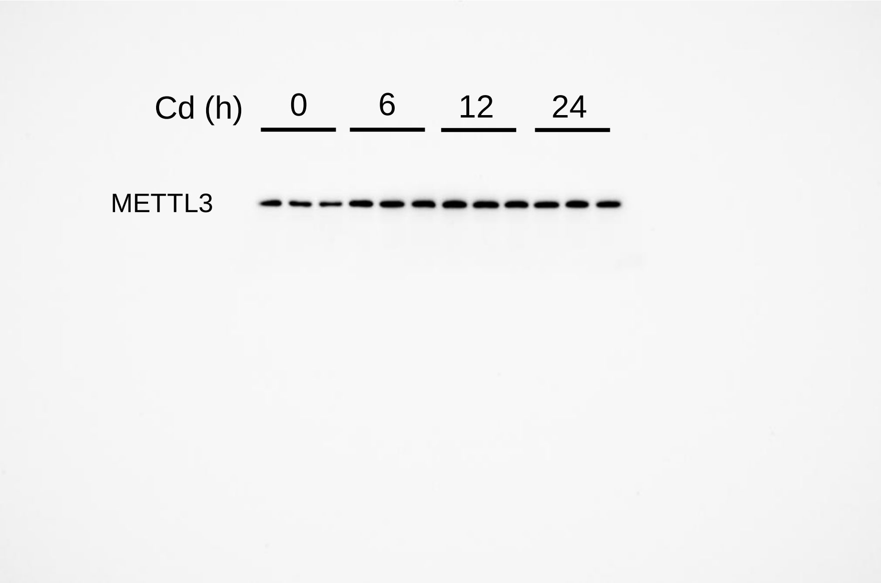

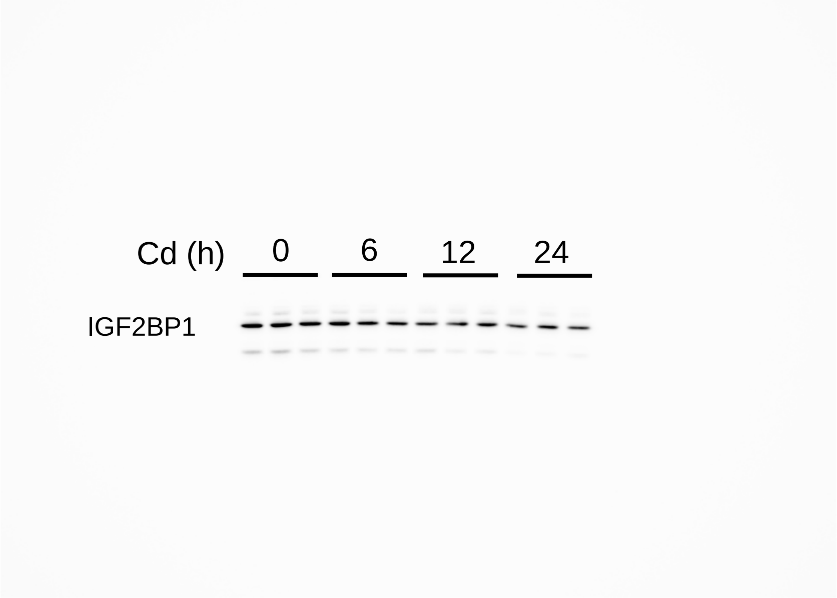

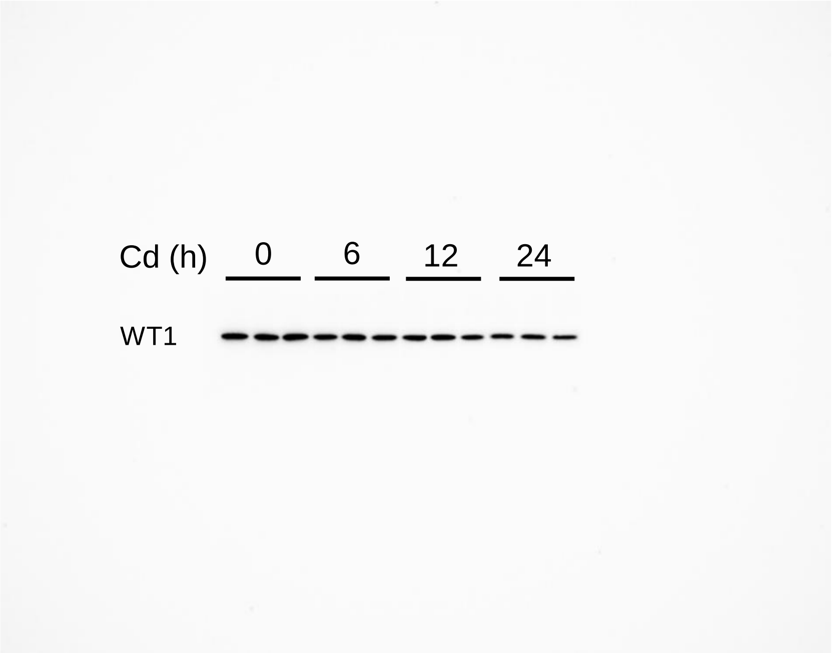

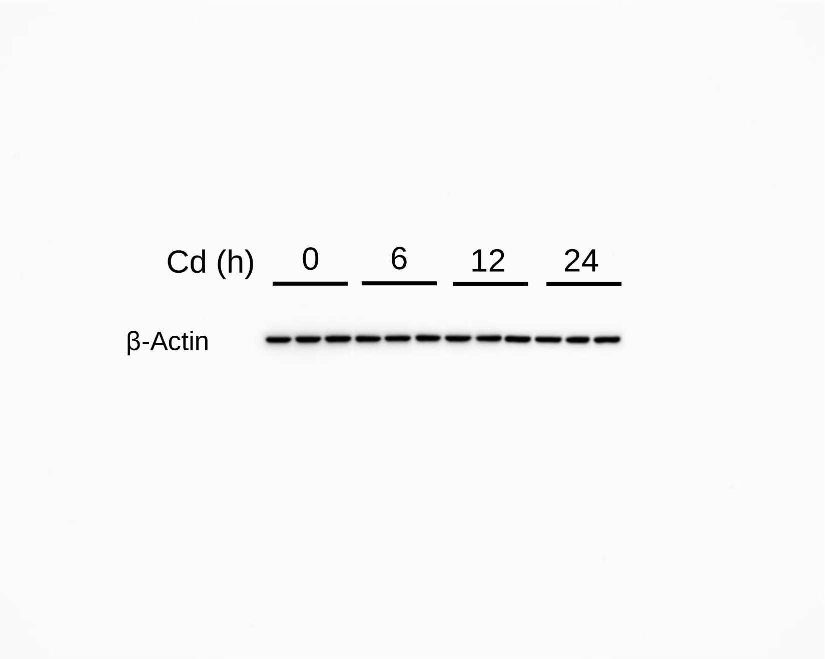

Supplementary Figure 8e

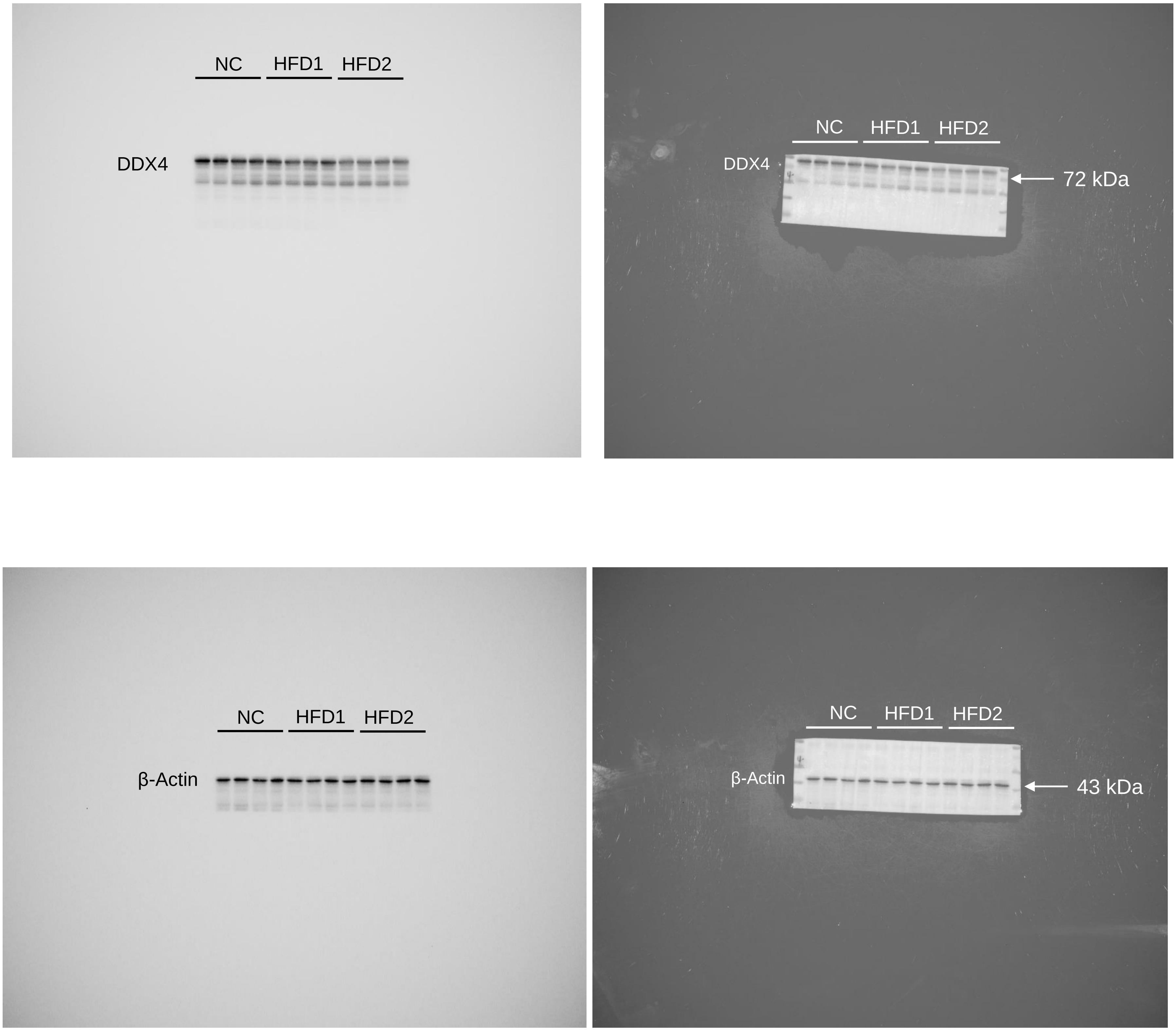

Supplementary Figure 8g

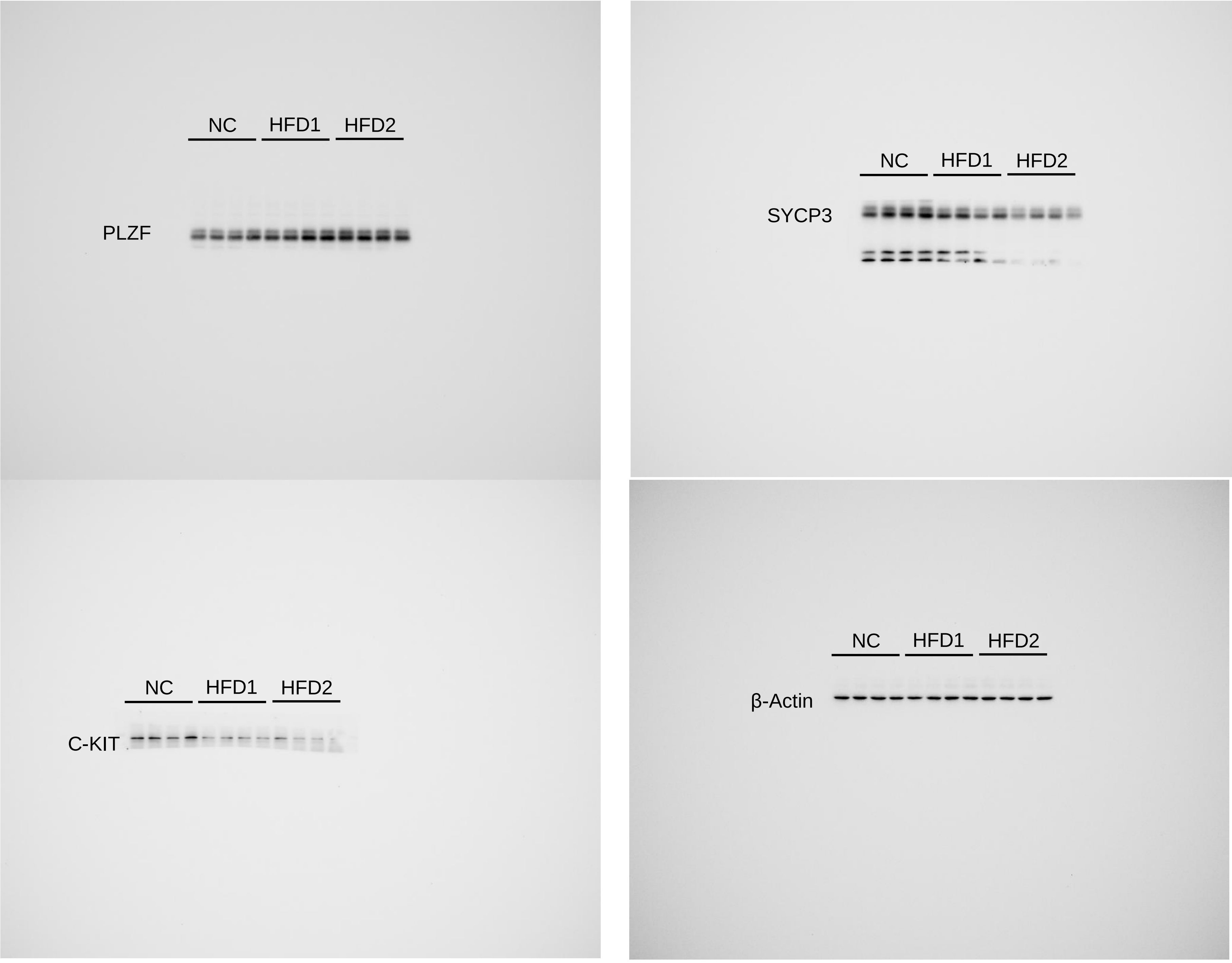

Supplementary Figure 9b

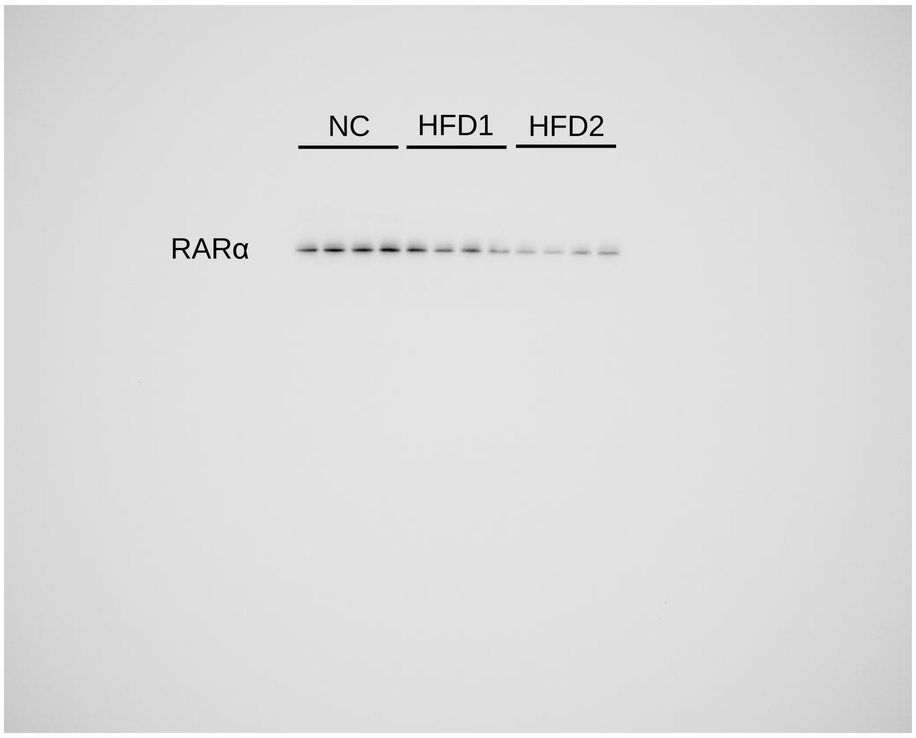

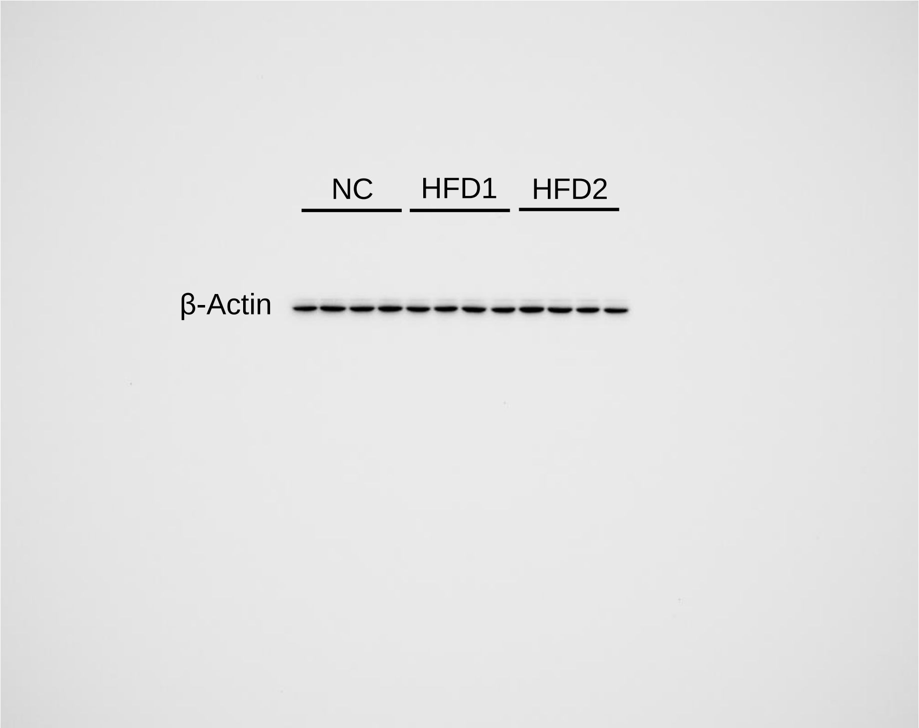

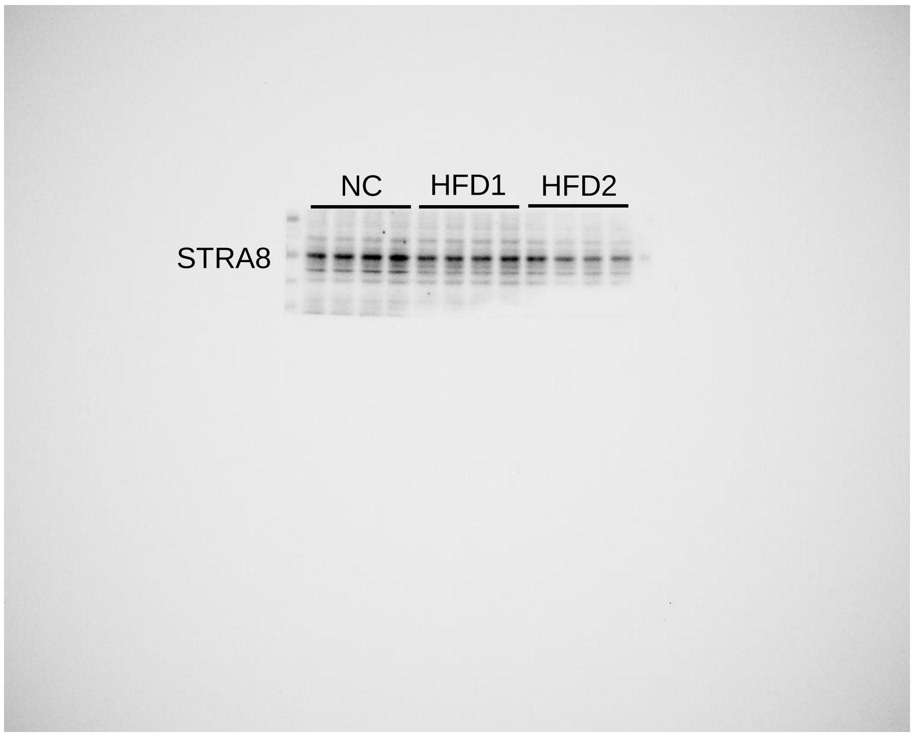

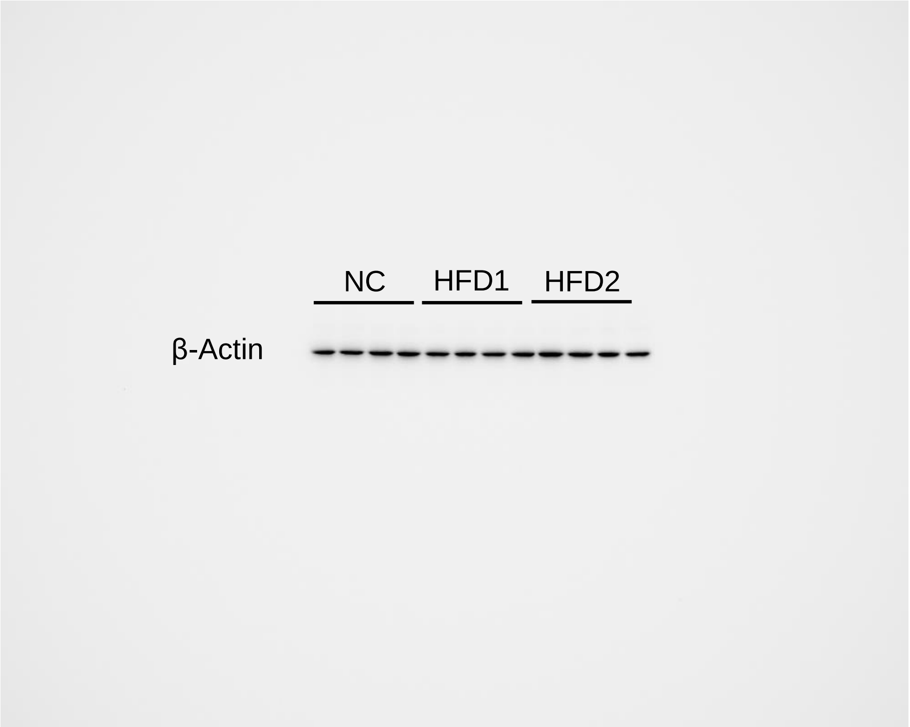

Supplementary Figure 9d

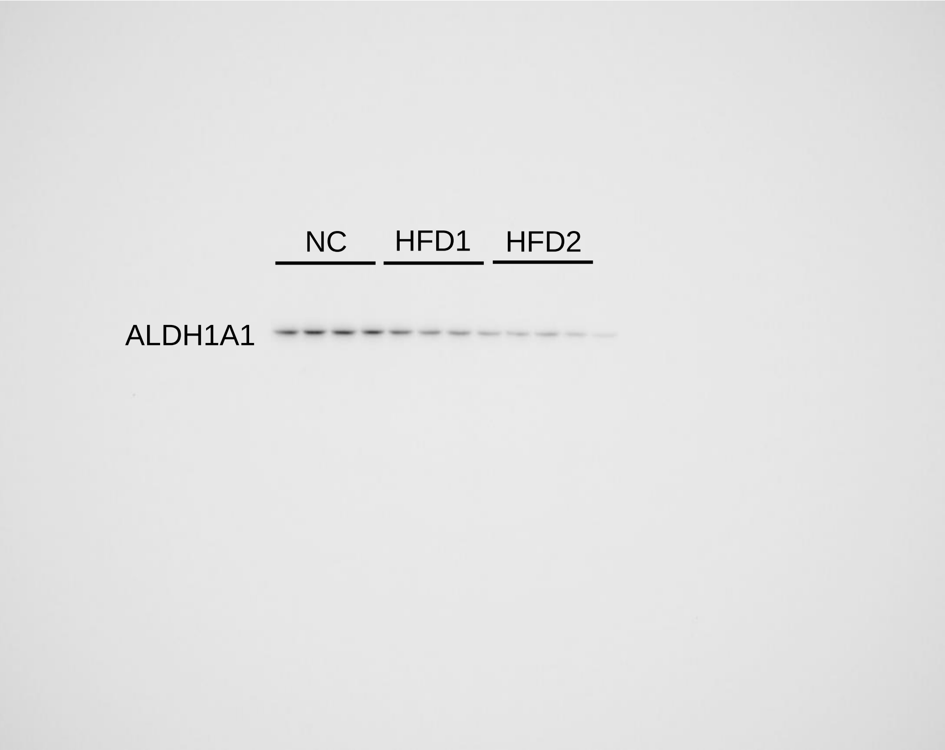

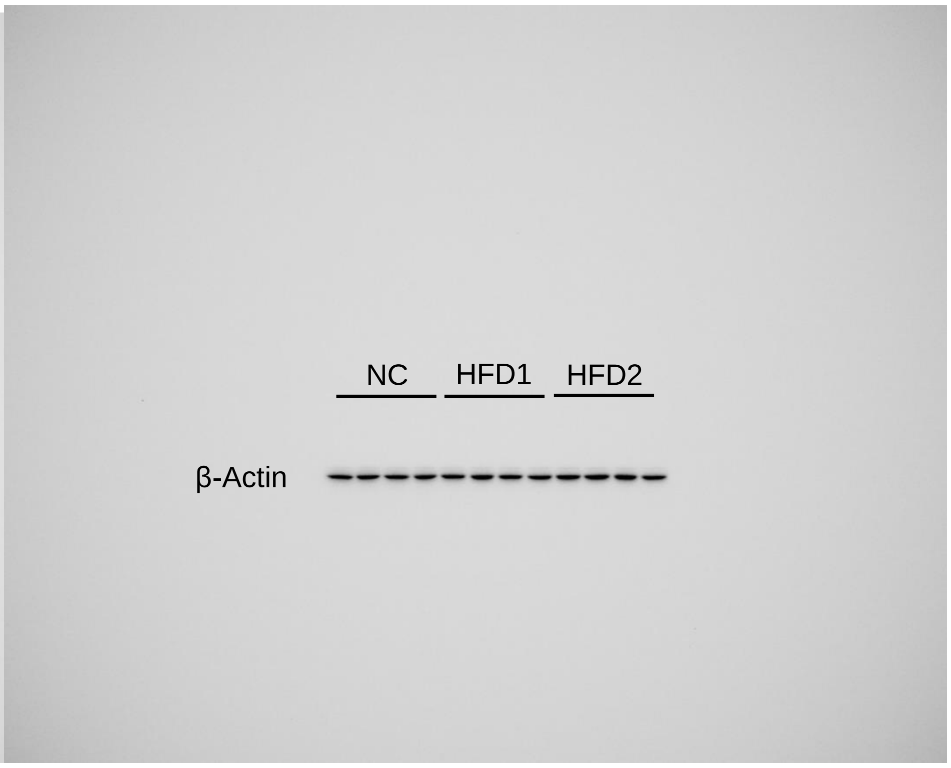

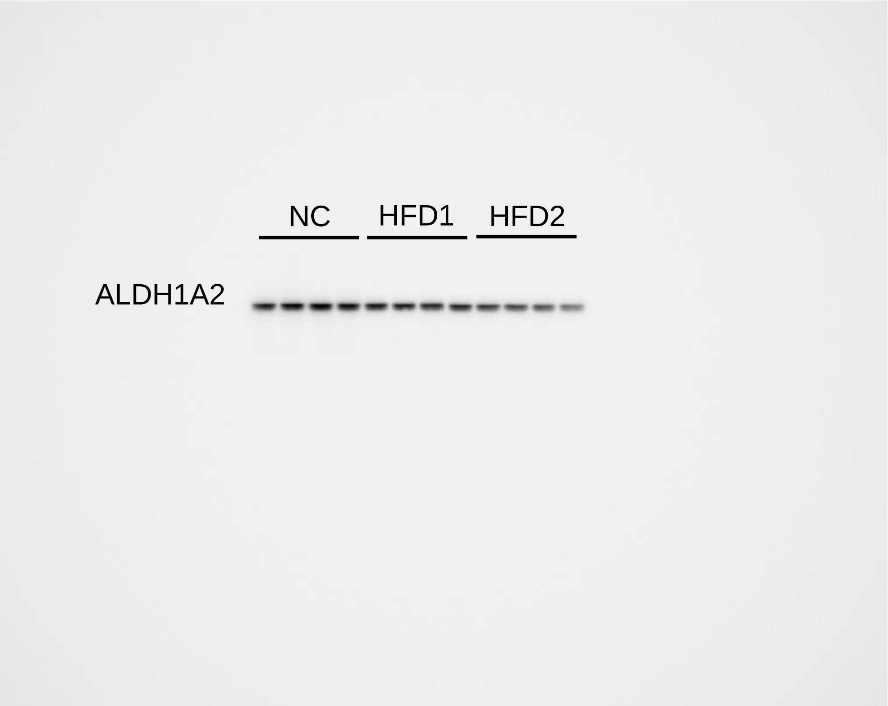

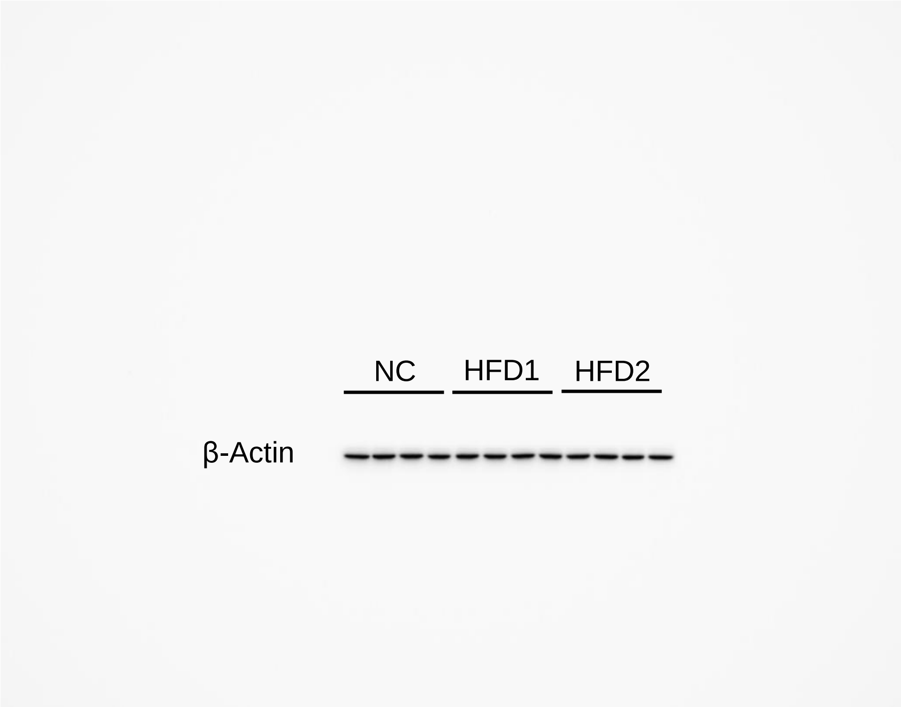

Supplementary Figure 9g

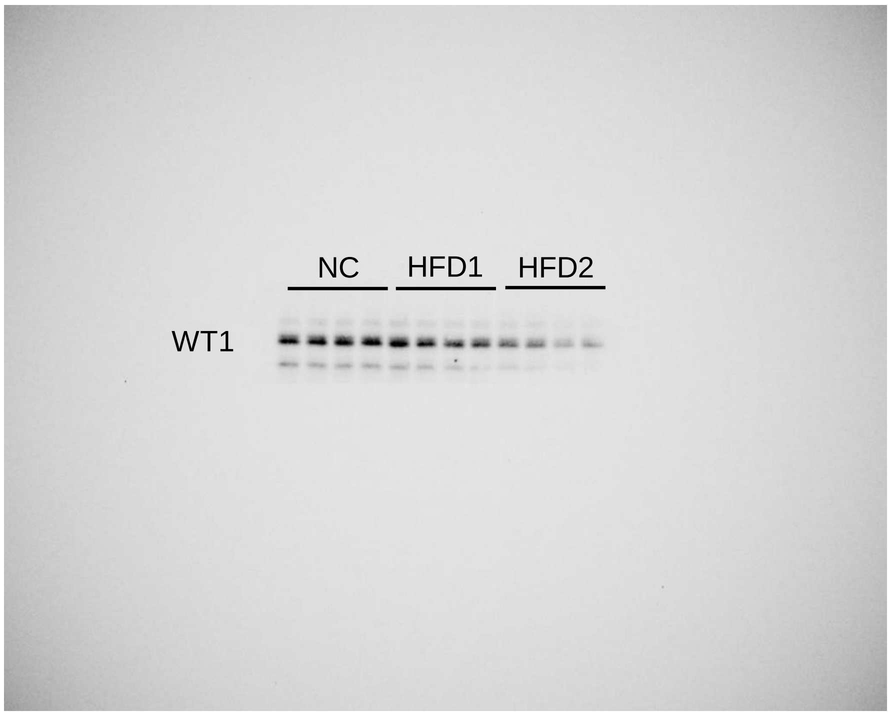

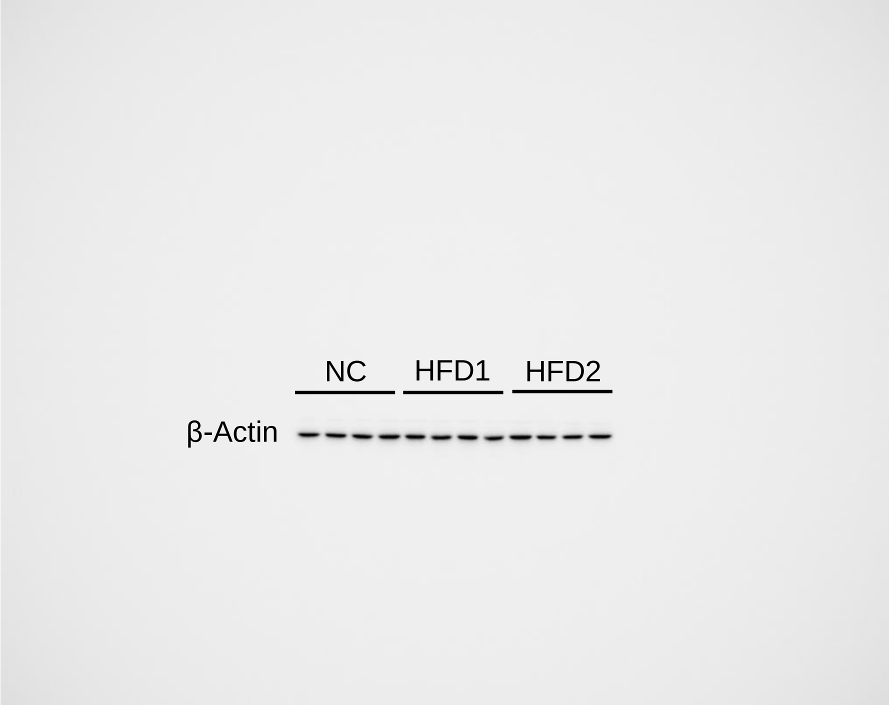

Supplementary Figure 10b

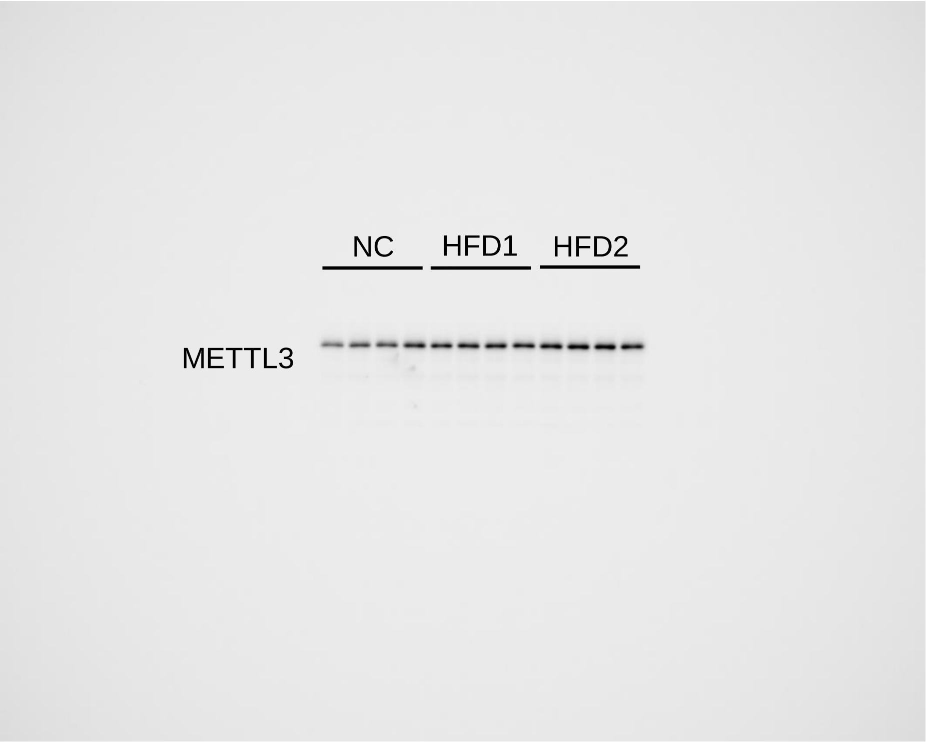

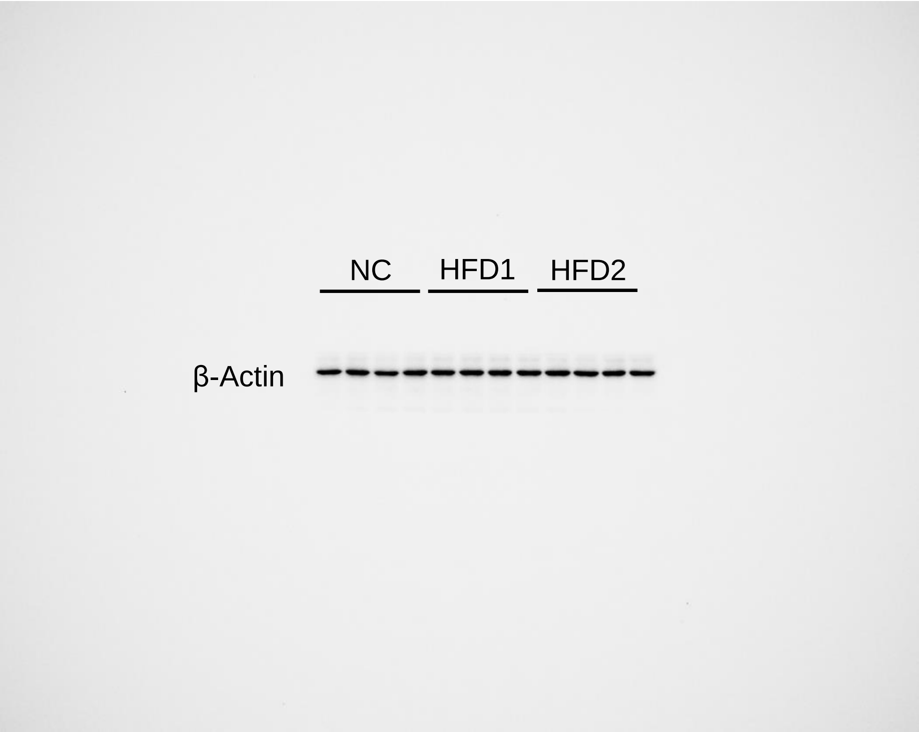

Supplementary Figure 10d

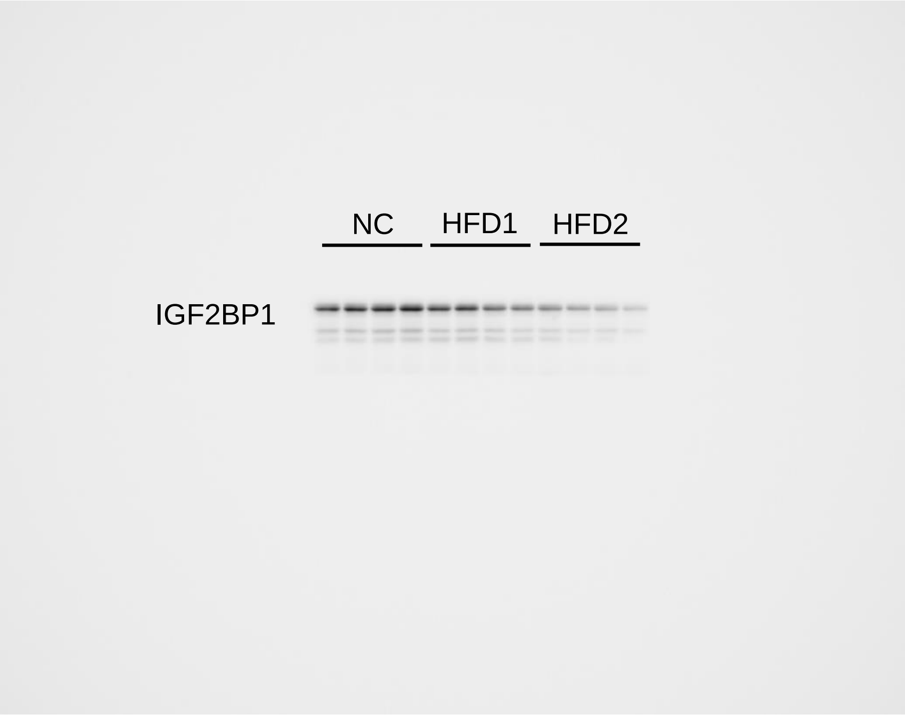

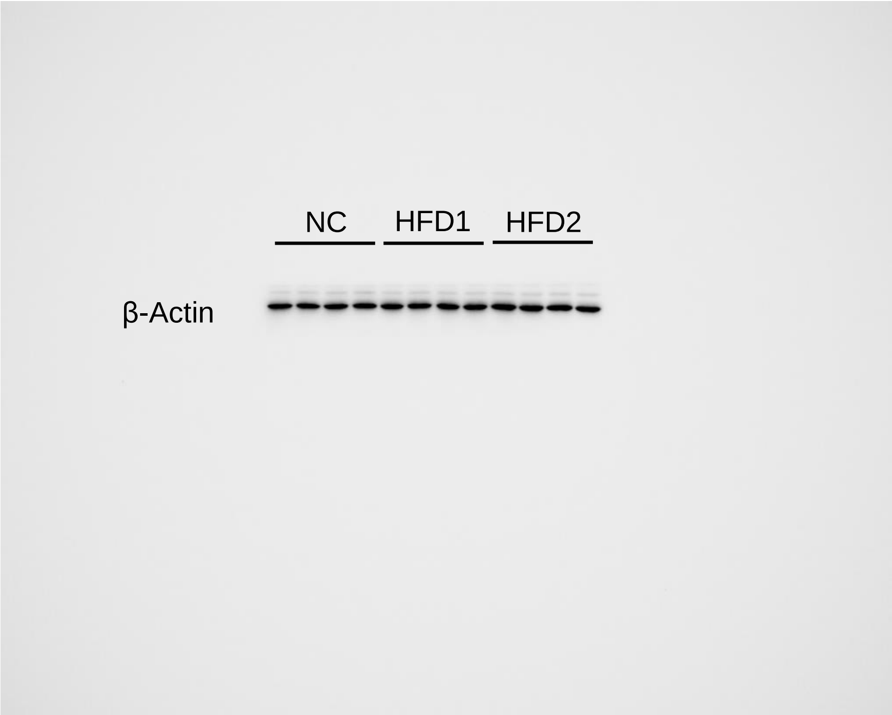

Figure 2b (sample 4-6)

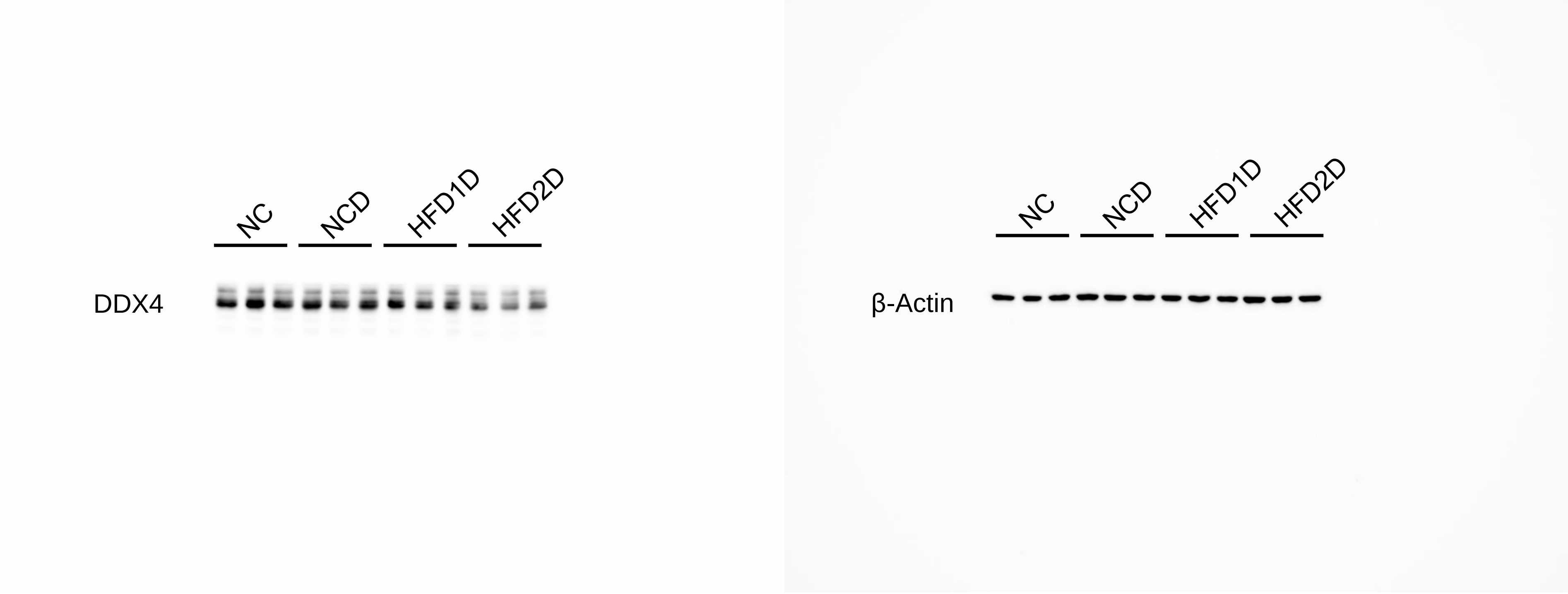

Figure 2e (sample 4-6)

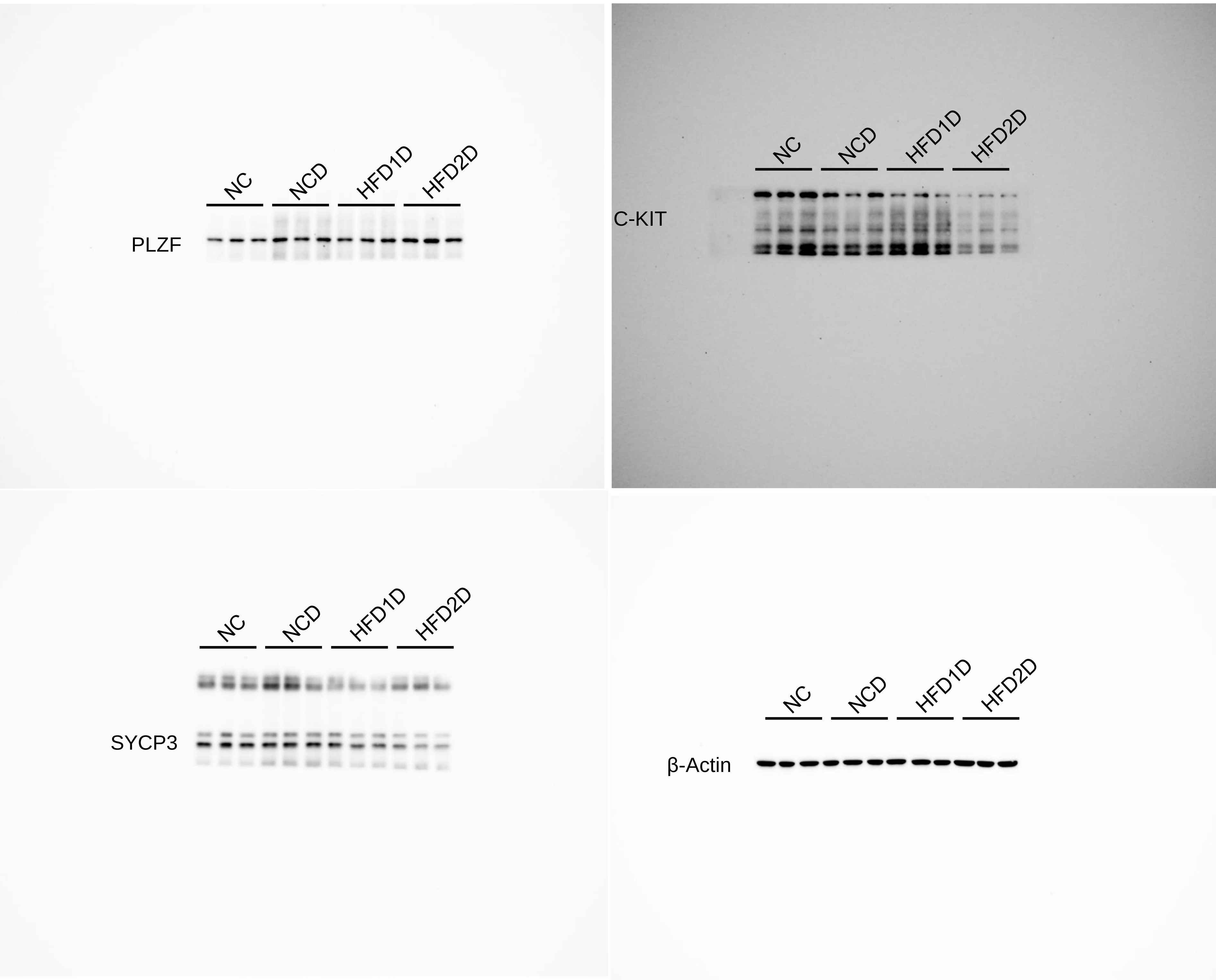

Figure. 3d (sample 4-6)

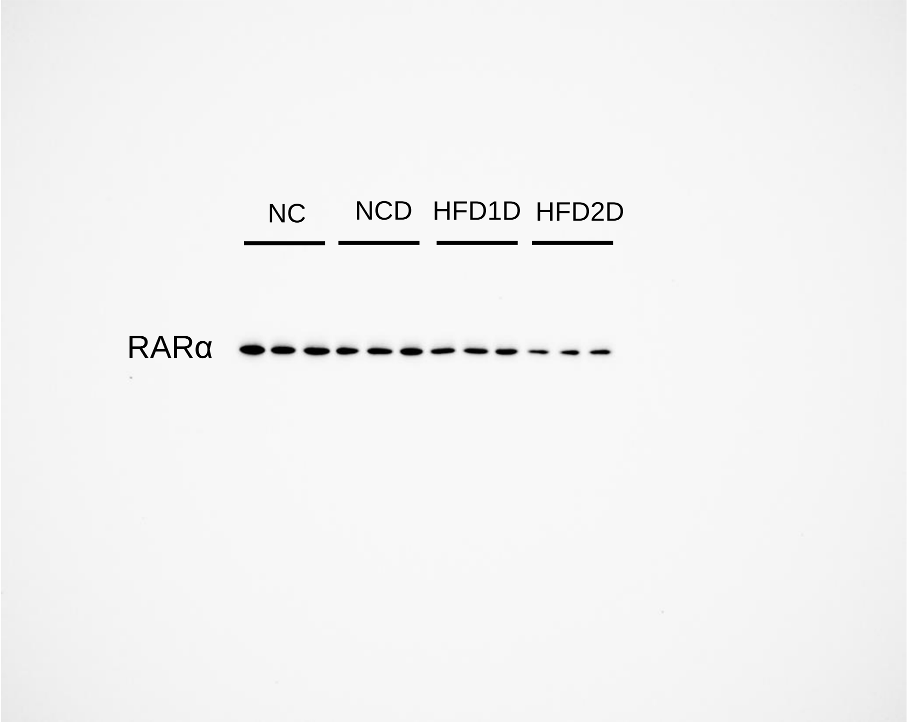

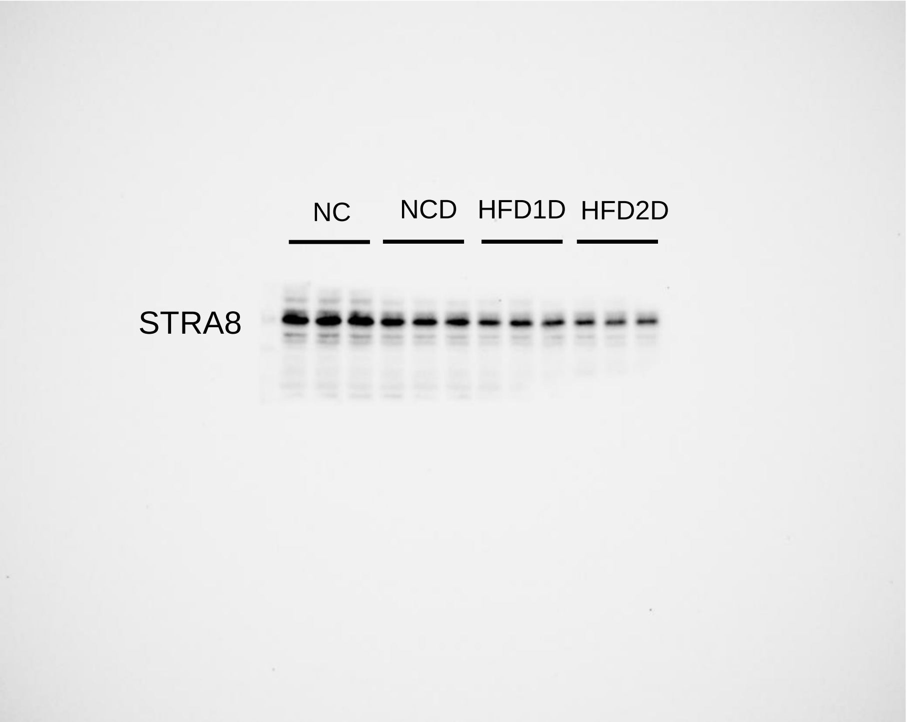

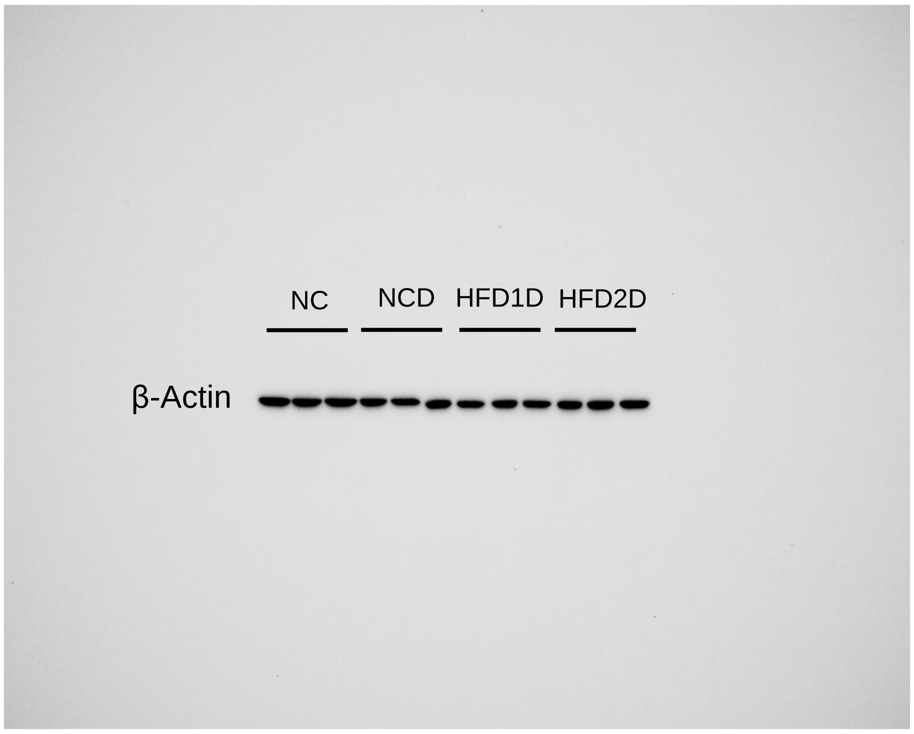

Figure 3g (sample 4-6)

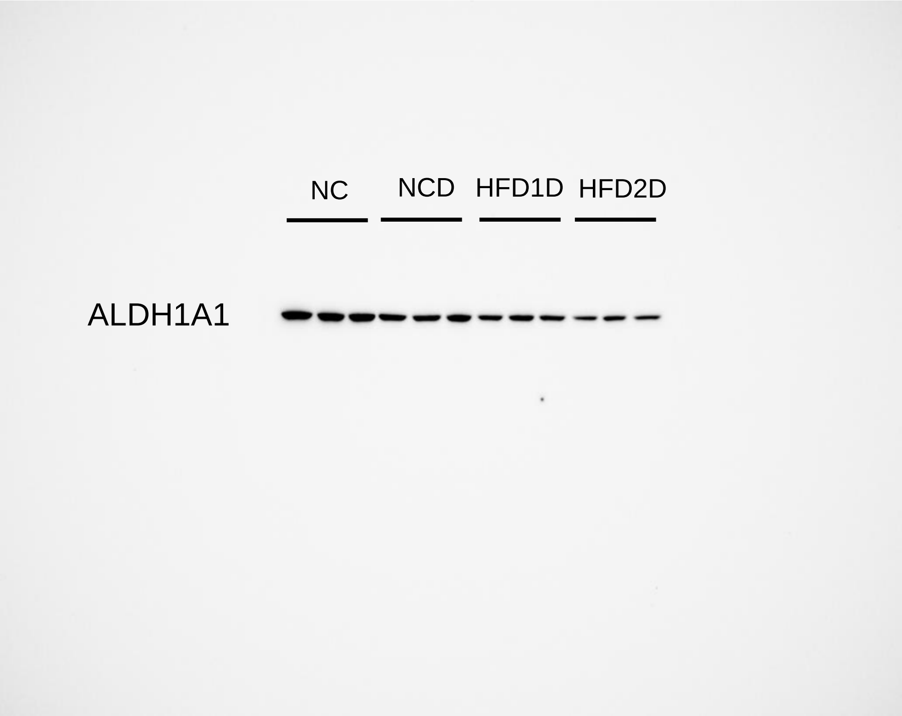

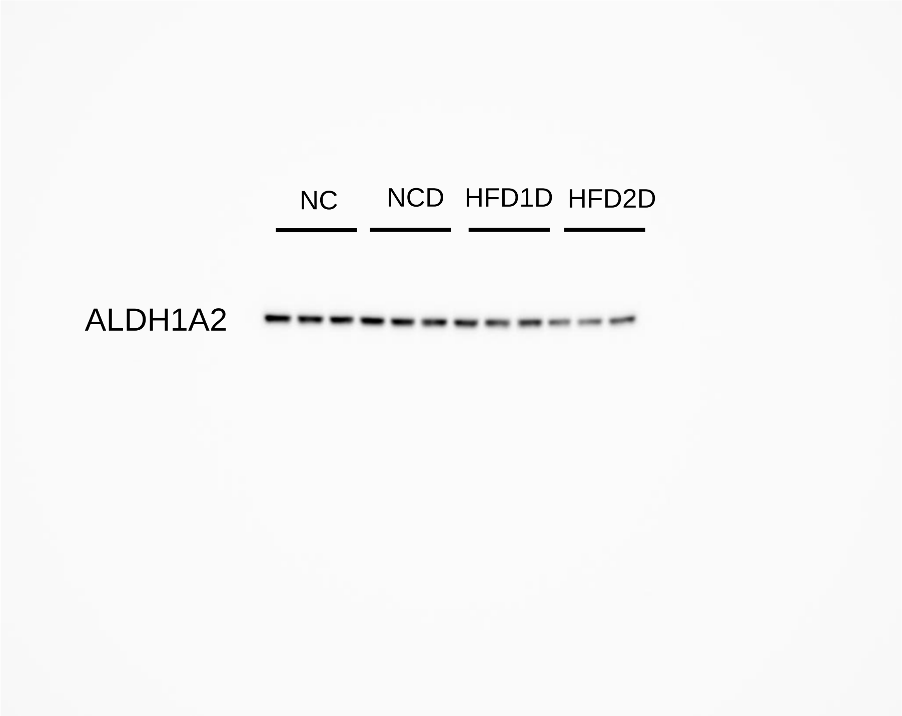

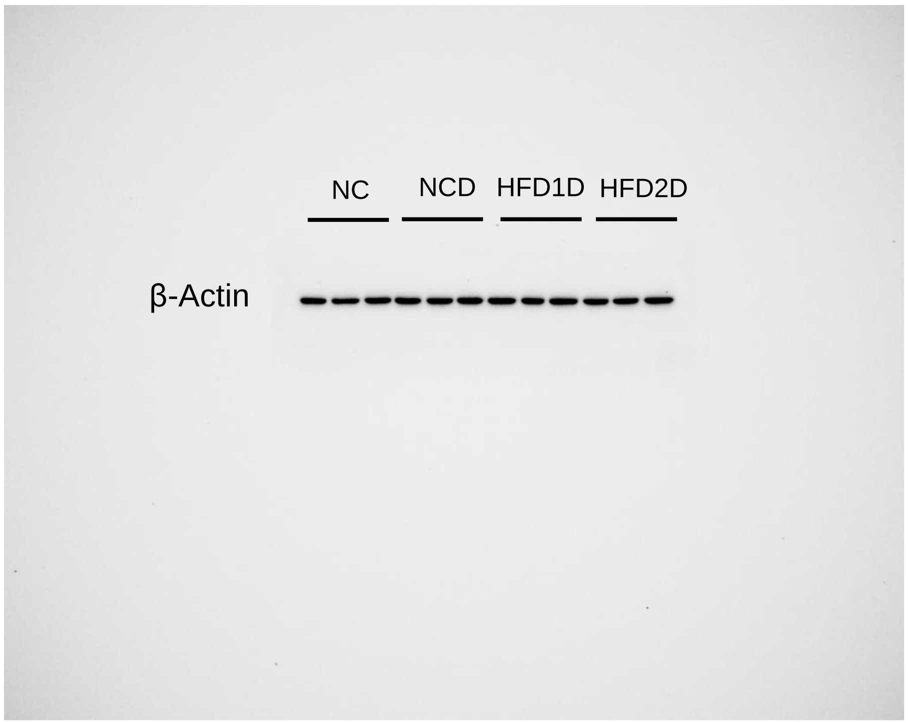

Figure 4c (sample 4-6)

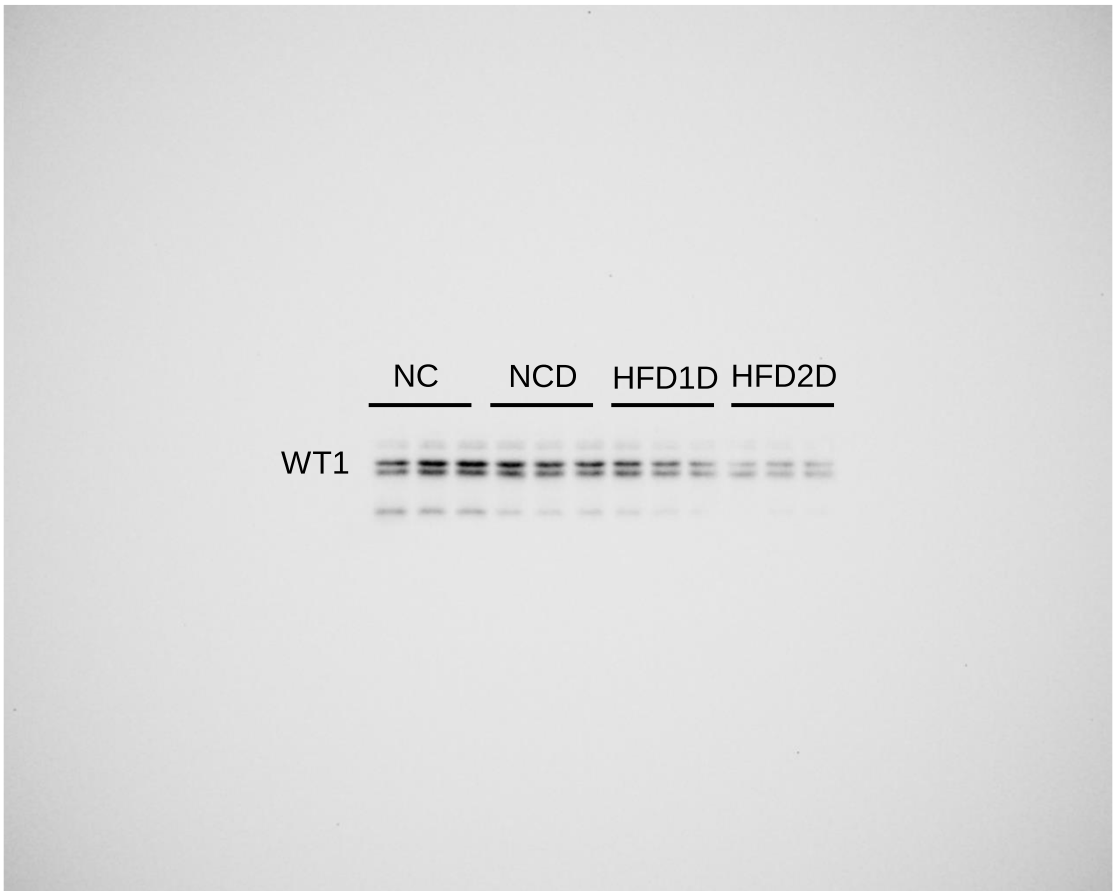

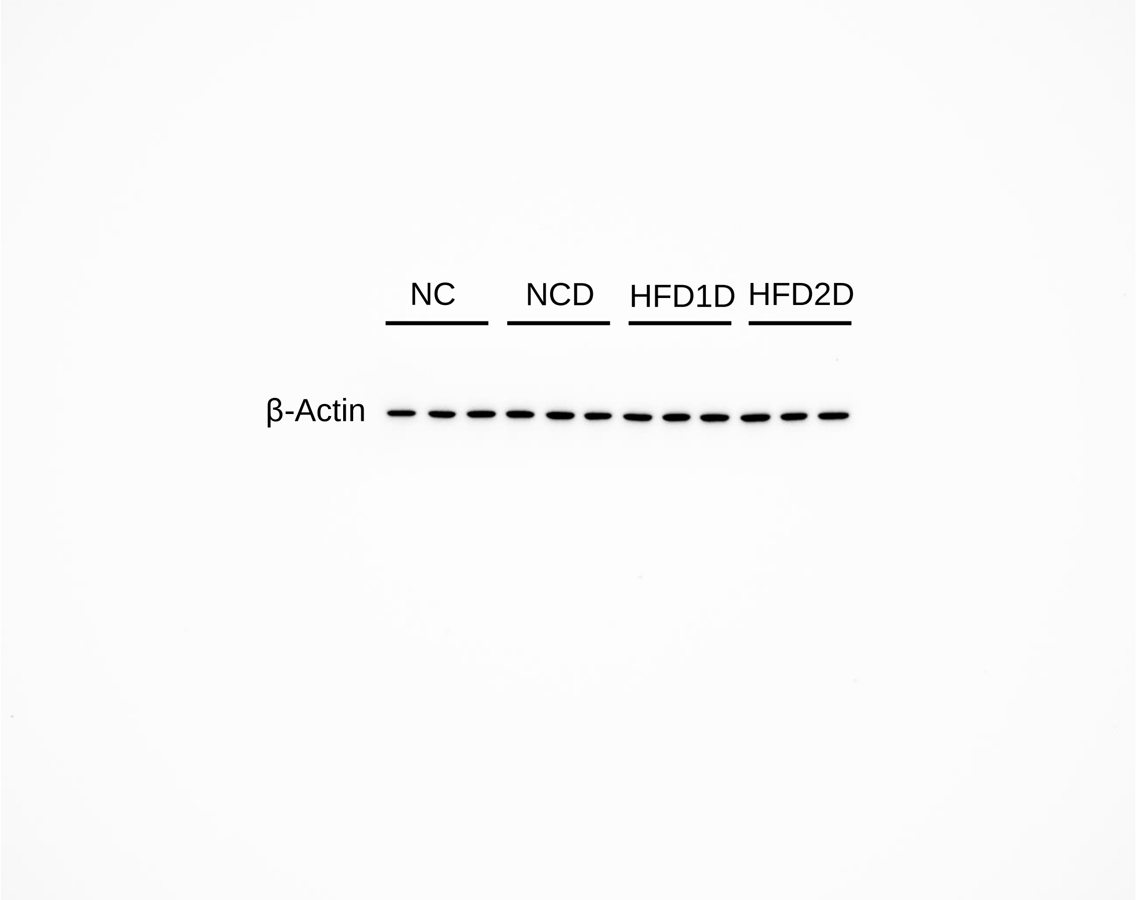

Figure 5d and h (sample 4-6)

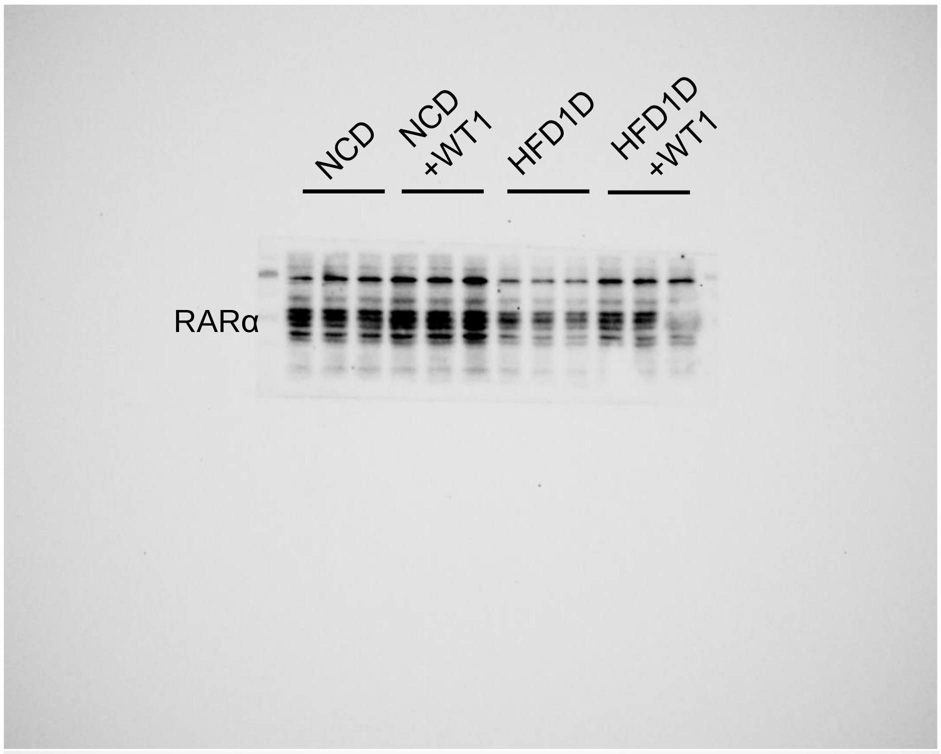

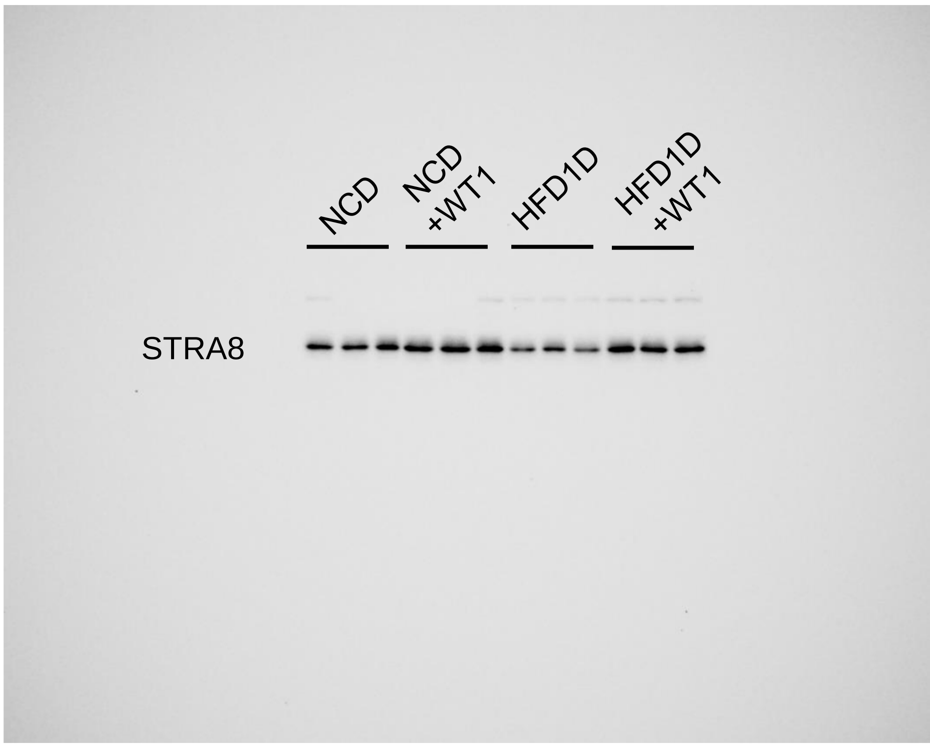

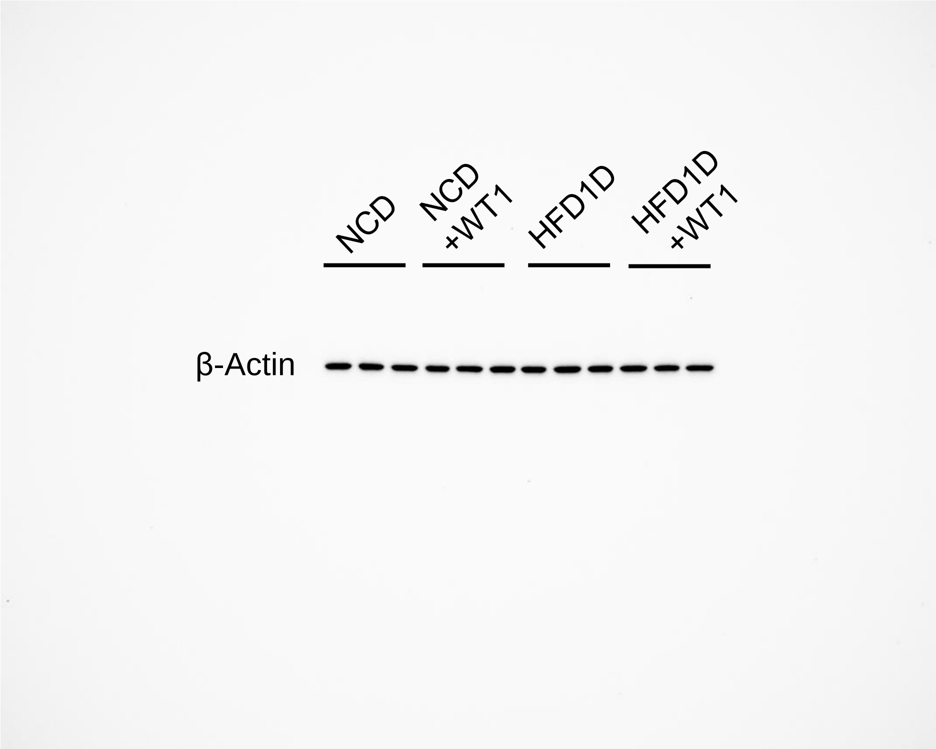

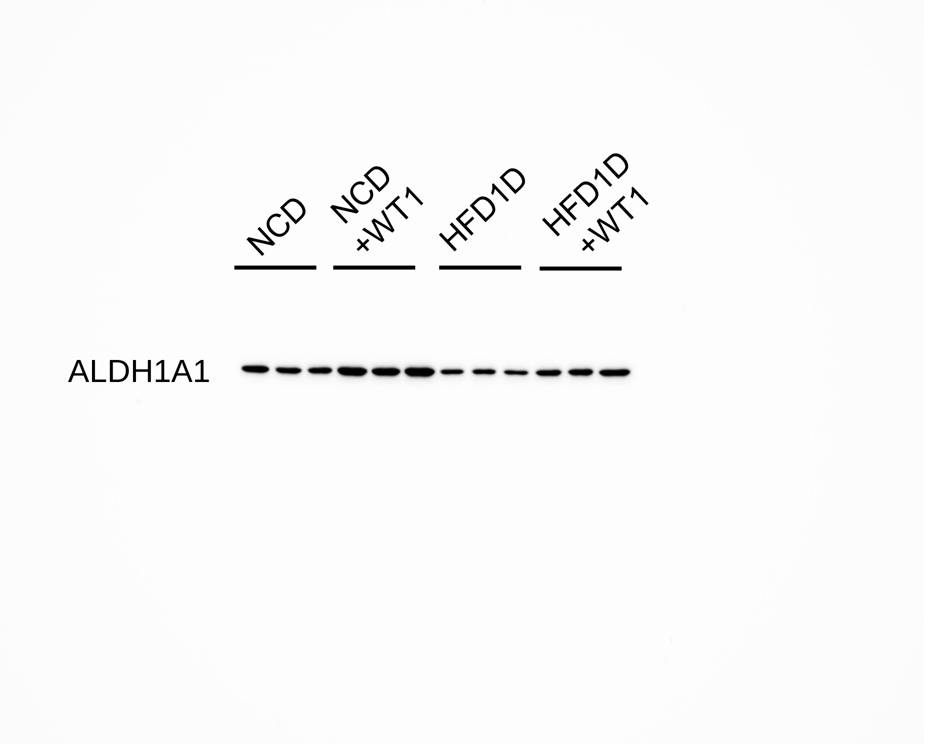

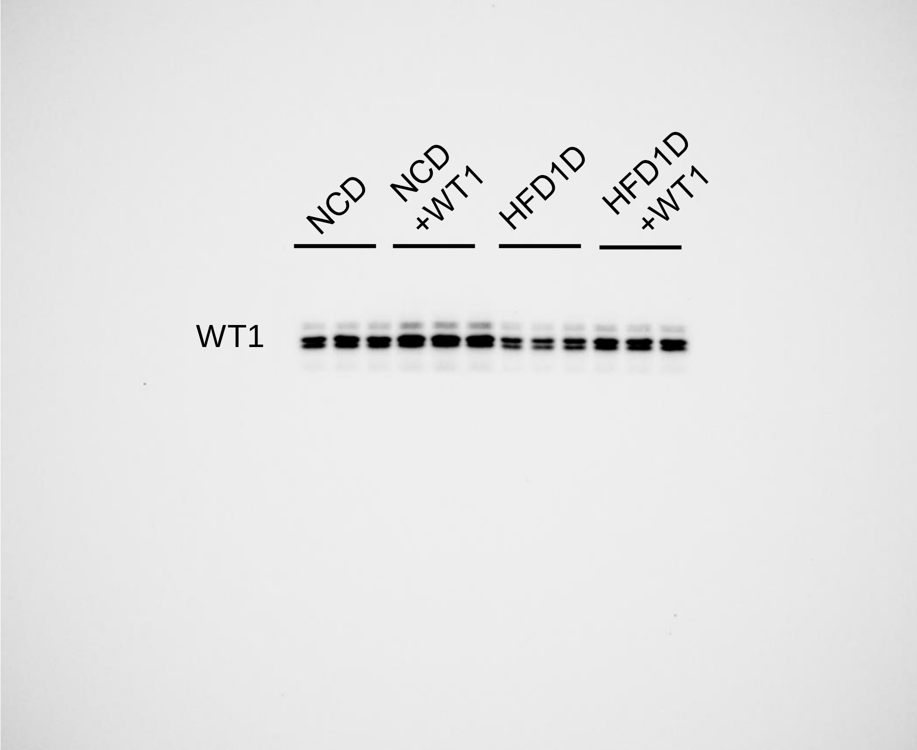

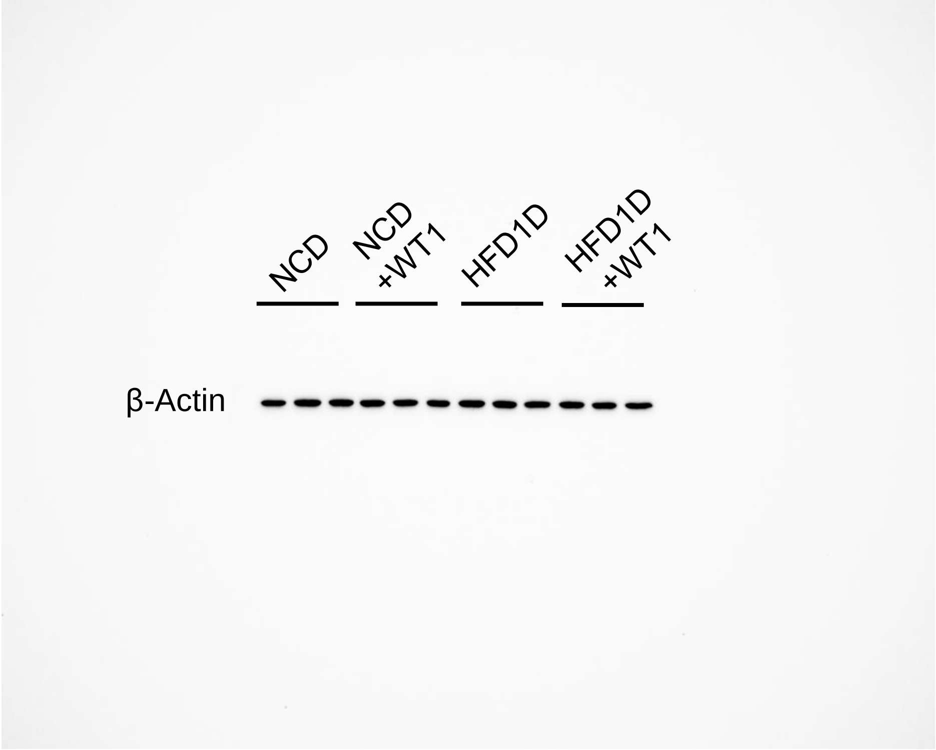

Figure. 6b and e (sample 4-6)

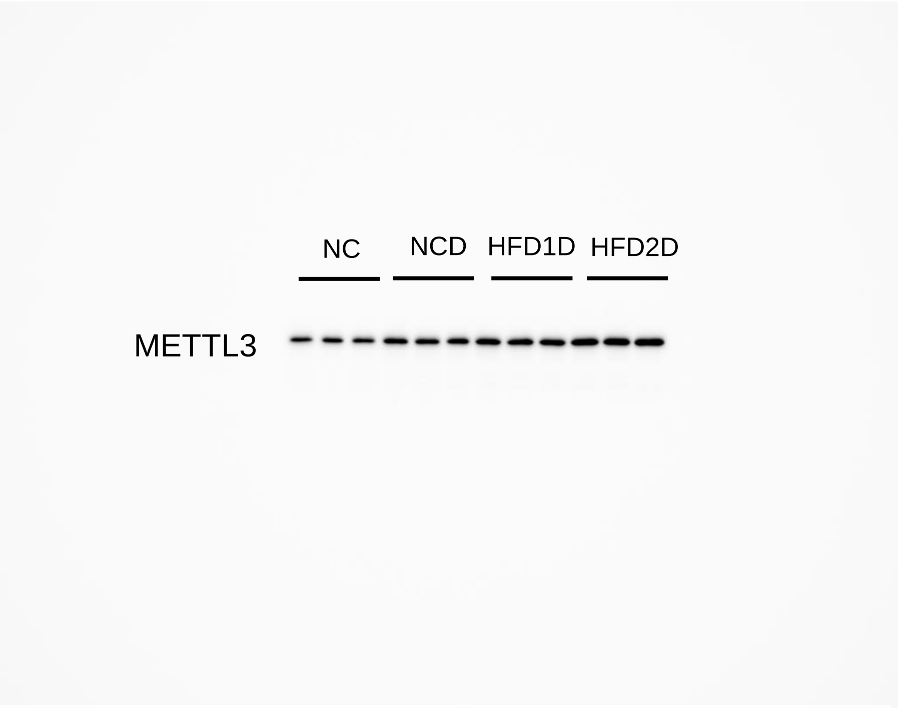

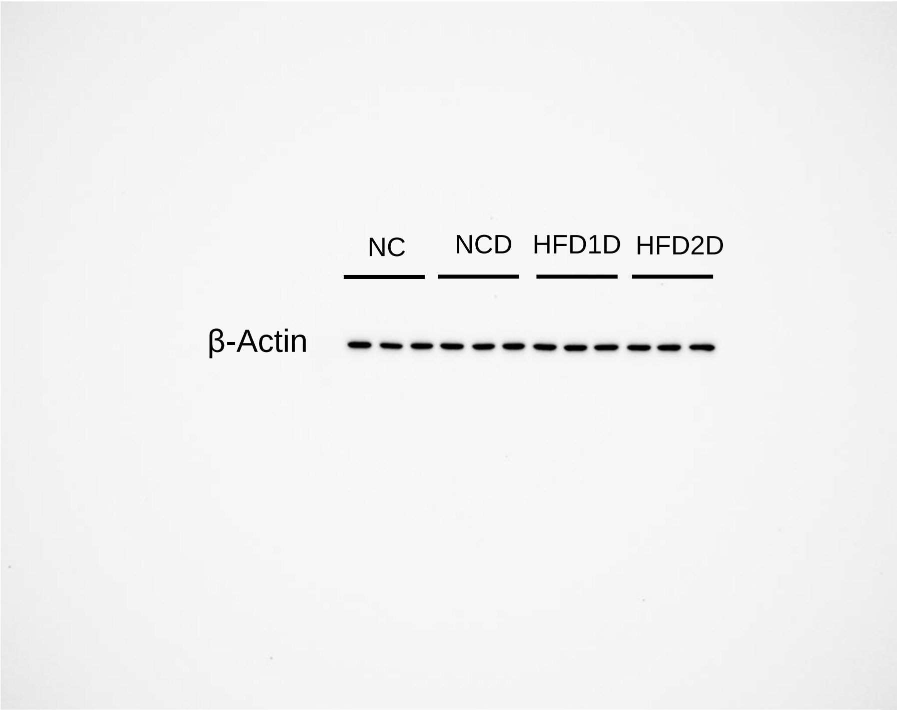

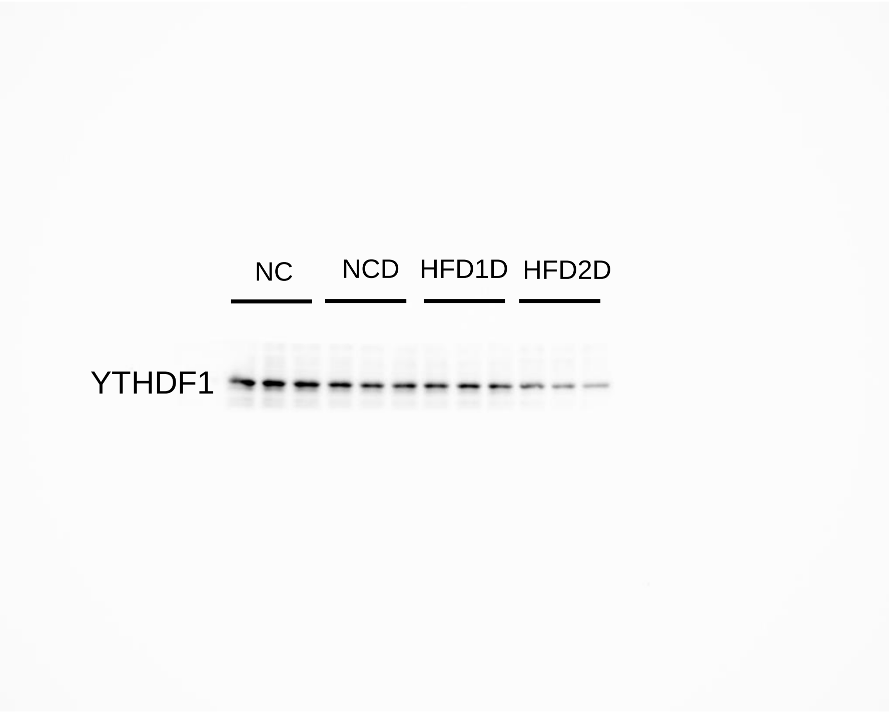

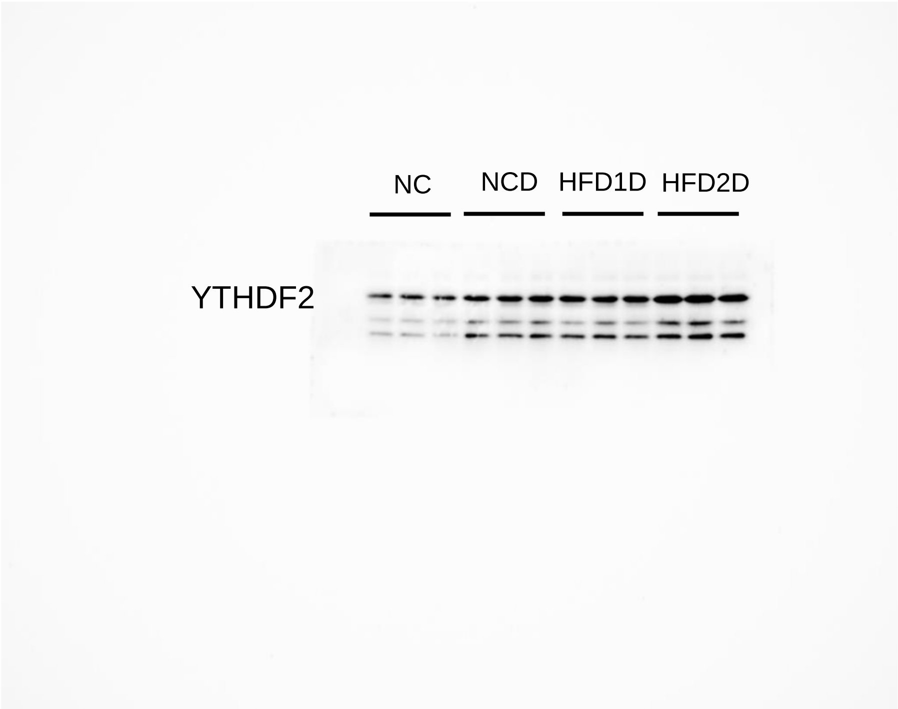

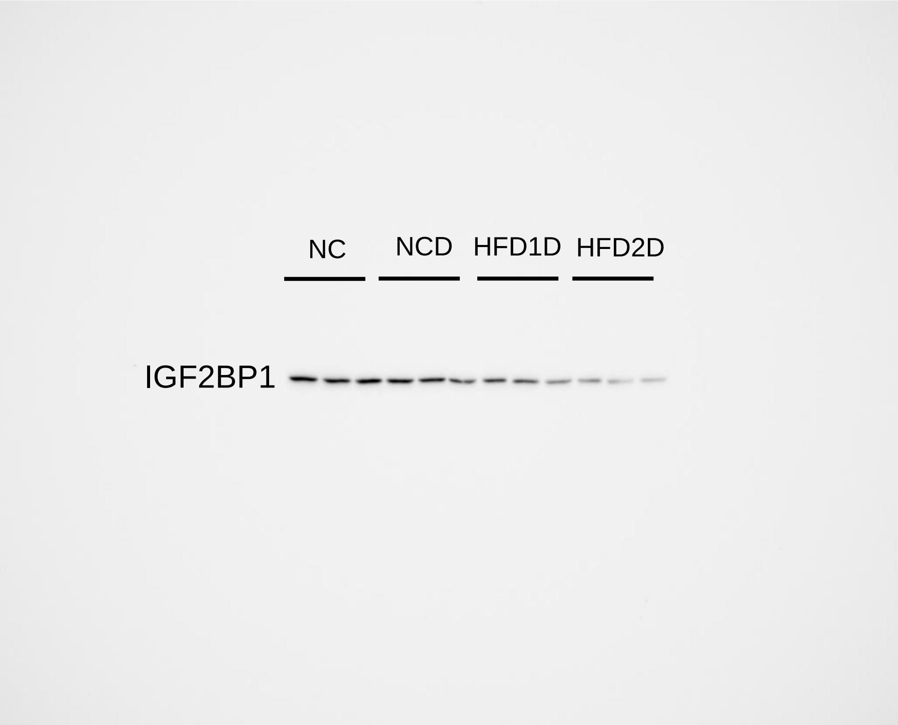

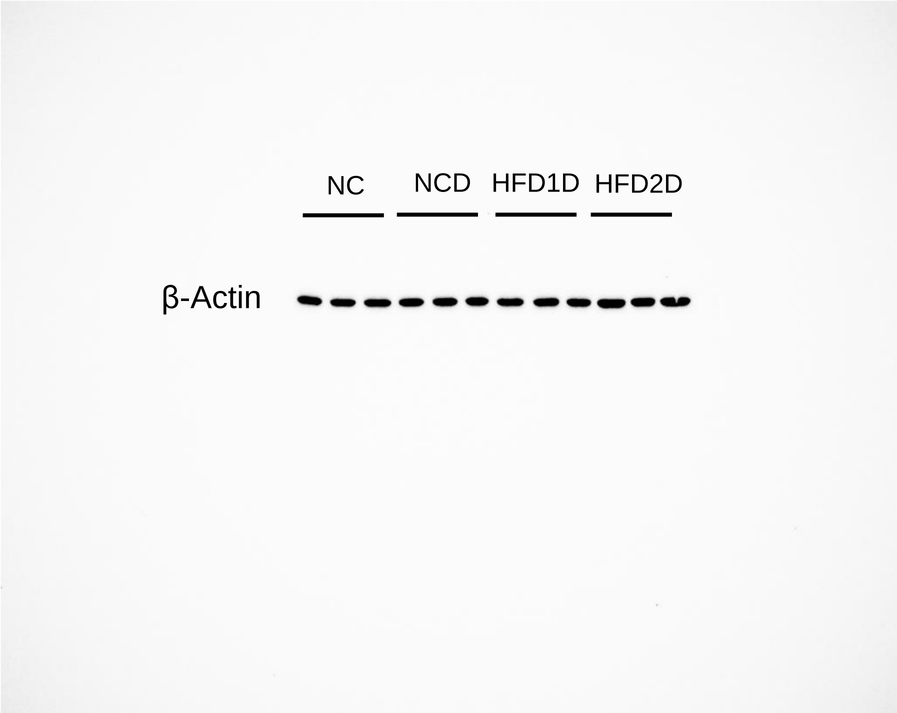

Figure 9d and h (sample 4-6)

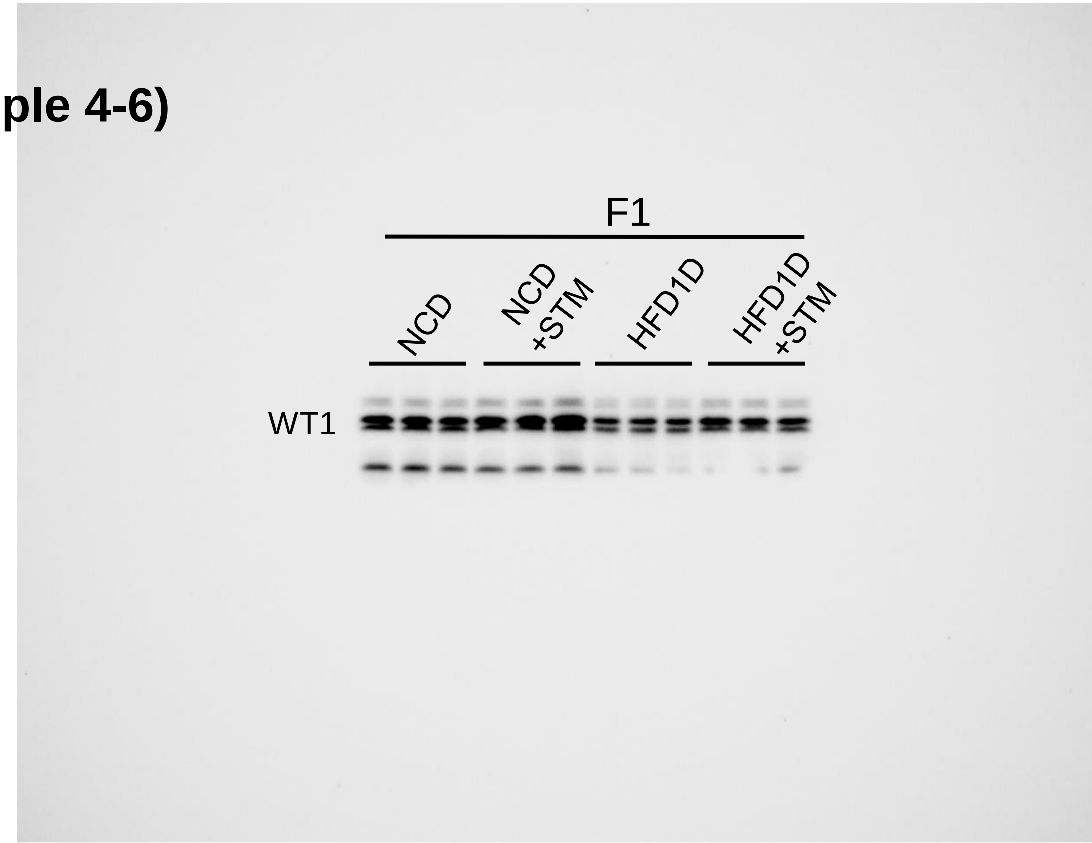

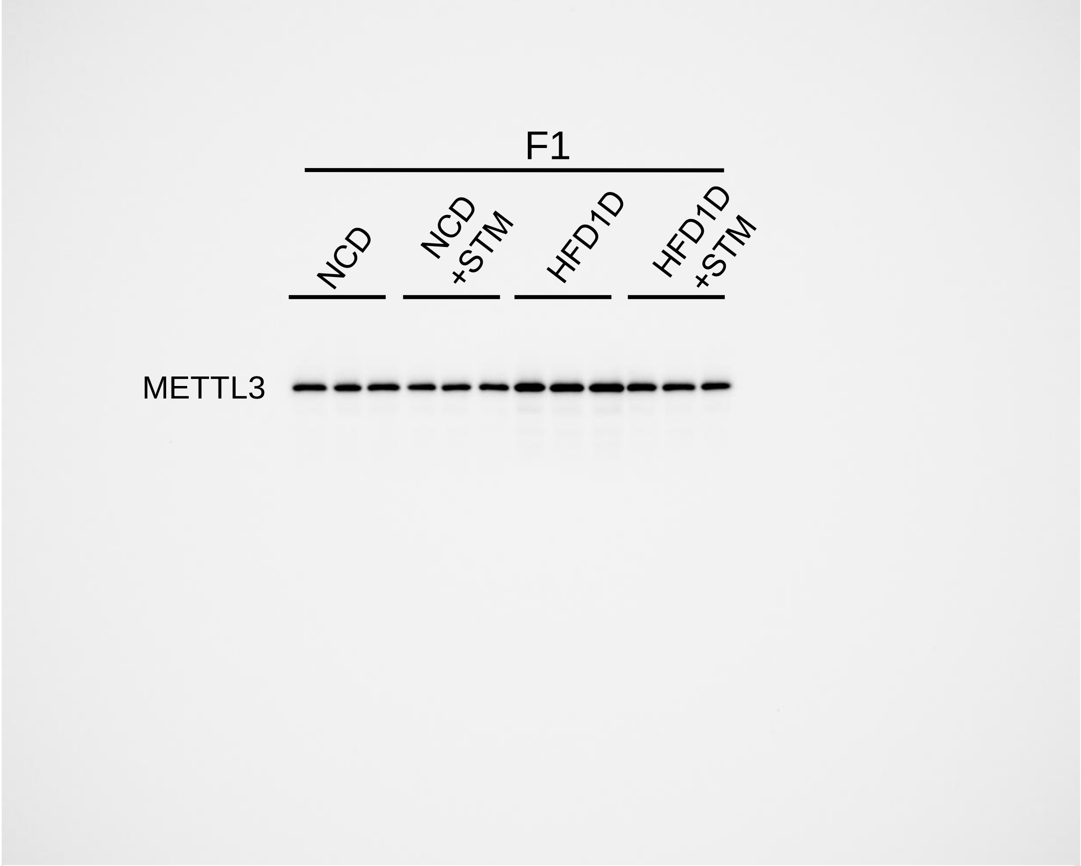

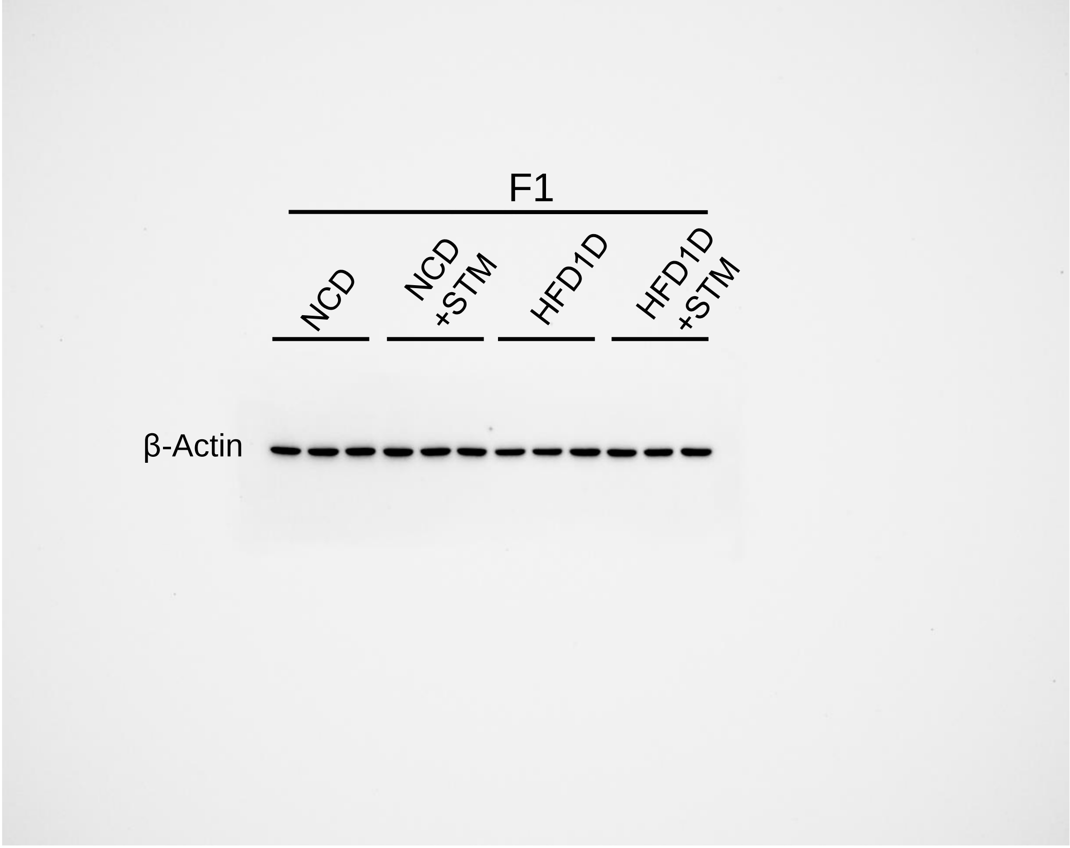

Supplement: Supplementary file 4 — Source data [file 41467_2024_45675_MOESM4_ESM.zip › Source data/Uncropped blots.pdf]
